# Global Plastic Industry Transition Addressing Key Drivers of the Triple Planetary Crisis

**DOI:** 10.1021/acs.est.5c08703

**Published:** 2025-09-02

**Authors:** Jing Huo, Zhanyun Wang, Christopher Oberschelp, Stefanie Hellweg

**Affiliations:** † Chair of Ecological Systems Design, Institute of Environmental Engineering, ETH Zürich, Zürich 8093, Switzerland; ‡ National Centre of Competence in Research (NCCR) Catalysis, ETH Zürich, Zürich 8093, Switzerland; § Empa-Swiss Federal Laboratories for Materials Science and Technology, Technology and Society Laboratory, St. Gallen 9014, Switzerland

**Keywords:** plastic industry, net-zero pathway, climate change impact, land-use-related biodiversity loss impacts, particulate matter-related health impacts, alternative feedstocks, plastic recycling, carbon capture and storage

## Abstract

The sustainable transition of the plastic industry—shifting from its fossil reliance and linear produce–use–dispose model—is imperative to minimize its contribution to the triple planetary crisis of climate change, biodiversity loss, and pollution. While previous studies assessed transition strategies in isolation, focused mainly on climate impacts, and neglected regional differences, our integrated model assesses transition strategies, globally and regionally, addressing the potential co-benefits and trade-offs across several key drivers of the triple planetary crisis. We note that other important impacts, such as microplastic leakage, remain to be quantified. Achieving a net-zero plastic industry by 2050 (1 Gt annual production) is technically feasible through lignocellulose residue-based feedstocks, recycling, and carbon capture. Meanwhile, this would require consuming all available global lignocellulose residues (2.3 Gt), early retirement of fossil infrastructure to avoid at least 0.35 Gt CO_2_-eq emissions, and ensuring grid decarbonization, presenting great challenges. Without internationally coordinated relocation of plastic production facilities or trade of biomass feedstocks or the derived intermediate chemicals, global net zero becomes unattainable. The global climate benefits through the transition come with trade-offs in higher land-use-related biodiversity loss and particulate matter-related health impacts, especially in regions with vulnerable ecosystems and dense populations, necessitating tailored regional solutions. Reducing primary plastics production could ease the transition, but unsustainable material substitutes need to be avoided.

## Graphical abstract

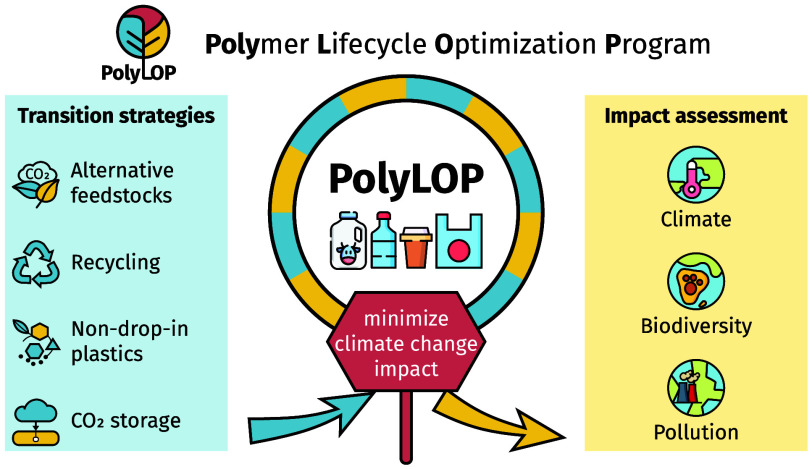

## 1 Introduction

Achieving net-zero greenhouse gas (GHG) emissions has become the cornerstone of keeping the global temperature rise below 1.5 °C.^1–3^ In addition to the climate challenge, biodiversity loss and pollution also belong to the so-called triple planetary crisis that calls for urgent global action.^4^ The global plastic industry contributes significantly to this crisis, with climate change impacts of approximately 2 gigatonnes (Gt) of CO_2_-equivalents (CO_2_-eq) (4.5% of global GHG emissions) and health impacts of 2.2 million disability-adjusted life years (DALY) due to particulate matter (PM) emissions.^5^ Over 90% of plastics production currently relies on fossil resources,^6,7^ including a growing share of coal-based production,^5,8^ while only 9% of plastic waste is recycled.^6^ With plastics production expected to more than double to 1 Gt by 2050,^9^ its contribution to the planetary crisis will only multiply without fundamental change.

Several transition strategies are being discussed to address climate change impacts; however, the interaction with and their combined effect on the triple planetary crisis remain unclear. On the production side, existing facilities can be retrofitted to directly capture CO_2_ emissions.^10^ Also, CO_2_ captured from other industries^11^ and lignocellulose biomass from agricultural and forest residues^12^ show promise as alternative carbon feedstock sources when combined with low-carbon energy sources.^9,10,13–15^ On the plastic waste management side, strategies include increasing mechanical recycling rates,^16^ supplemented by chemical recycling.^17^ Additionally, while care must be taken not to substitute plastic with more carbon-intensive materials and while rebound effects must be avoided,^18^ reducing plastic demand effectively lowers overall environmental impacts.^15,19^

While previous studies have explored various transition strategies,^9,15,19–23^ they have predominantly focused on net-zero GHG emissions and limited combination of transition strategies, leaving important gaps in the comprehensive understanding of technically feasible paths to a sustainable future (detailed in Table S25). Many analyses focus on mechanical recycling and biobased drop-in plastics (which are identical to conventional plastics but produced using renewable sources)^9,15,19–23^ but overlook CO_2_-based production and non-drop-in alternatives such as polylactic acid (PLA). Some studies assume unrealistically high recycling rates of up to 95%,^14,24^ while other studies show a practical upper limit of around 30% for mechanical recycling.^16,25^ Furthermore, existing studies often lack detailed regional analysis^14,23^ and thus fail to account for variations in the availability of alternative feedstocks, electricity carbon footprints, and production capacities. Few studies considered some regional conditions^10,21^ but separately addressed single transition pathways without a holistic analysis of combined pathways. Critically, these studies oversimplified the assessment by assuming, e.g., a uniform land use area per unit biomass output regardless of location and biomass type, thereby negating the benefits of regional differentiation.

Finally, trade-offs among different aspects of the triple planetary crisis have been insufficiently considered.^9,19,22,23^ Bachmann et al.^14^ extended environmental impacts beyond climate change using the planetary boundary framework, but human health was not covered by this work. In addition, their reliance on global average impact factors fails to capture crucial regional variations in ecoregion vulnerability and local environmental pressures—factors that significantly influence biodiversity loss impacts,^26,27^ as identical resource consumptions can have vastly different impacts across regions.^4^

We aim to close these gaps and support a sustainable transition of the global plastic industry by 2050 by providing a more holistic assessment of transition pathways toward net-zero emissions. Uniquely, we integrate spatial variations in plastic production amount, feedstock availability, and regionalized impact assessments, enabling the identification of climate-optimal scenarios globally and by region, while simultaneously assessing co-benefits and trade-offs related to land-use-related biodiversity loss and PM-related human health impacts, which represent key drivers of biodiversity loss and human health impacts globally.^28,29^ With a more comprehensive picture of transition strategies that consider regional specificities and multiple environmental impacts, this study offers a guide to policymaking and industry strategies to transform the plastic industry by addressing key drivers of the triple planetary crisis across different geographic regions.

## 2 Methods

### 2.1 Goal and Scope

The goal of the study was to evaluate (1) the technical feasibility of achieving net-zero greenhouse gas emissions for the global plastic industry and (2) the associated trade-offs or co-benefits for land-use-related biodiversity and PM-related health impacts across different geographic contexts. The functional unit was the satisfaction of the global plastic demand in 2050, a year by which many countries commit to the achievement of net-zero emissions. Plastics production was examined globally and across 26 regions as defined in the IMAGE Integrated Assessment Model (IAM)^9^ (Figure S2). Fourteen types of plastics were considered in this study (Table S1), including the nine conventional types that make up 95% of the plastics market share today^30^ and five biobased non-drop-in types that have the potential to replace the conventional ones in the market in various application sectors, identified by Nessi et al.^31^ Future plastics production by share and sector was derived from the plastic demand forecast by sector^9^ and the current share of each plastic type for each sector^16,30^ (detailed in Section S1.2.3).

The system boundaries included the acquisition and processing of carbon feedstocks and other raw materials, production of plastics and their upstream chemicals, and recycling or final disposal of waste plastics (Section S1.1 and Figure S1). Waste plastics calculations were based on 2020 plastics production volumes, projections for 2050, and the average sector-specific lifetime of plastic products from Klotz et al.^25^ (detailed in Section S1.2.4). Since the same plastic types are produced in all scenarios, the use-phase impacts are the same and can be omitted (albeit with differences due to a small production of non-drop-in plastics, which may have diverging use-phase impacts).

### 2.2 Polymer Lifecycle Optimization Program (PolyLOP)

To assess the feasibility of a net-zero transition in the global plastic industry, PolyLOP (Polymer Lifecycle Optimization Program) was developed. This open-source Python-based tool, utilizing the Gurobi solver for linear optimization, was designed to optimize process routes and quantities across the plastics production chain and end-of-life treatment. PolyLOP can perform single-objective optimization to minimize individual impact or multiobjective optimization to explore trade-offs between different impact categories by generating Pareto curves (Section S1.6.1).

PolyLOP provides gate-to-gate inventory data sets for 133 unit processes, with each process encompassing raw material and energy consumption, as well as emissions data for 1 unit of main product.

### 2.3 Process Inventories

The following paragraphs provide an overview of the process inventories in the model (the full list of processes is described in Sections S1.3 and S1.4). For processes that generate byproducts, economic allocation was employed in the main analysis. Economic allocation involves distributing all inventory flows among products and byproducts based on their relative economic values. This approach was chosen due to its ability to reflect market-driven production decisions and its widespread acceptance in life cycle assessment studies of chemical processes.^32^

#### 2.3.1 Fossil-Based Plastics Production

Conventional plastics are traditionally produced from natural gas and petroleum via refinery and steam cracking processes^7^ and were included in PolyLOP.

#### 2.3.2 Alternative Feedstock-Based Drop-In Plastics Production

This study examines plastics production from two alternative carbon feedstocks that are not in competition with food production: CO_2_ from industrial point sources and lignocellulose residues (detailed in Section S1.2.5).

The supply chain for drop-in plastics mirrors that of conventional plastics to a large extent with methanol serving as an intermediate. Methanol can be produced through CO_2_ hydrogenation^33–35^ or via gasification of lignocellulose residues into syngas, followed by catalytic conversion of this syngas to methanol.^36–39^ Subsequently, the same range of base chemicals can be produced through methanol-to-olefins^40^ and methanol-to-aromatics^14^ technologies. Additionally, ethylene can be obtained through the dehydration of ethanol,^41^ whereby the ethanol itself is produced from the fermentation of pretreated lignocellulose residues^42^ (see Figure S3).

High-purity-process CO_2_ emissions can be captured during biomass gasification and fermentation. CO_2_ capture from other emission sources, such as combustion processes for heat production, was not included in the model due to the lower CO_2_ concentrations, which result in less greenhouse gas savings and higher capture costs.^12,43^

#### 2.3.3 Biobased Non-Drop-In Plastics Production

Biobased non-drop-in plastics are those that follow distinct production pathways compared to conventional plastics, although they can perform the same functions across various applications. The majority of the non-drop-in plastics considered in this study are biodegradable and only suitable for single-use applications. Due to the need for additional additives in durable applications, some of which may result in elevated environmental and health risks,^44^ the application of biodegradable plastics in durable applications was excluded.

Substitution factors quantify how much non-drop-in biobased plastic is required to replace a unit weight of conventional plastic in specific applications. These factors were derived from the existing literature,^31^ supplemented by a density proxy approach, in which the ratio of the density of conventional plastics to that of the non-drop-in alternatives was calculated. This ratio provides a reasonable proxy for substitution in volume-based applications^31^ (detailed in Tables S1 and S8).

#### 2.3.4 Plastic Waste Treatment

Plastic waste was assumed to undergo either incineration or recycling (detailed in Section S1.3.3), assuming successful global efforts to eliminate plastic littering. Incineration was modeled without energy recovery. In the context of a net-zero 2050 scenario, where the electricity grid is expected to have a very low carbon footprint, using incineration to substitute for the electricity grid mix would offer minimal carbon credits.

Mechanical recycling was considered for all conventional plastics excluding polyurethanes, following the process inventory by Klotz et al.^16^ Mechanically recycled plastics were assumed to replace virgin plastics of the same type. Chemical recycling through gasification and pyrolysis was modeled for waste polyethylene, polypropylene, and polystyrene.

#### 2.3.5 Storage of Captured CO_2_

The captured process CO_2_ can be either utilized for chemical production (CCU), stored permanently underground (CCS), or released into the atmosphere if CCS is excluded from the system and CO_2_-based production is not chosen by the model. The process inventory of transport and storage of captured CO_2_ is modeled after Sacchi et al.’s.^45^

#### 2.3.6 Utilities

The process inventory incorporated heat sources from natural gas and lignocellulose residues. The electricity grid (excluding solid biomass-fired electricity generation) was considered as a direct input into the PolyLOP model. Solid biomass-fired electricity generation fueled by lignocellulose residues was included in the model as a separate process, allowing the model to choose the optimal use of lignocellulose residues (as fuel or chemical feedstock).

### 2.4 Regionalized Life Cycle Impact Assessment

Three key environmental impacts were evaluated: climate change, land-use-related biodiversity loss, and PM-related health impacts. These impact categories address the main drivers of the triple planetary crisis.

The life cycle impacts assessment relied on the recommended methods from the Global Life Cycle Impact Assessment Method (GLAM) initiative of the United Nations Environment Programme (UNEP).^26,27,46^ Climate change impacts were measured using the 100 year Global Warming Potentials (GWP100) from IPCC^47^ and Cherubini et al.^48^ (the latter for biogenic CO_2_-emissions), expressed in kg CO_2_ equivalents (detailed in Section S1.5.1). For land-use-related biodiversity loss (detailed in Section S1.5.2), regionalized impacts from biomass production and harvesting were considered, as these activities account for over 90% of land-use-related biodiversity loss,^49^ measured by the global Potentially Disappeared Fraction of species (PDF). Fossil fuel extraction impacts were modeled by using global average mining areas and characterization factors. Regionalized PM-related health impacts were measured using Disability-Adjusted Life Years (DALYs). Spatially explicit characterization factors of PM-related health impacts^26^ for emissions of PM2.5, SO_2_, NO_*x*_, and NH_3_ were aggregated to the regional resolution (Figure S2) that matches this study.

The cumulative environmental impacts of the plastic industry were calculated by summing the impacts caused by direct emissions within the system boundary and the cradle-to-gate impacts of all raw materials added to the system. This includes the regionalized impacts of lignocellulose residues and other minor crop inputs, quantified based on Huo et al.^12^ (detailed in Section S1.5.2). CO_2_-feedstock captured from other industries is considered burden-free for the plastic industry, counting it as a waste treatment measure for the source industries.^11^ The climate change impacts of all other raw materials in 2050 were quantified with the premise v1.4.1, a tool for prospective life cycle assessment.^45^ It projects the ecoinvent 3.8 database (cutoff by classification system model) into the future based on the Shared Socioeconomic Pathway 2 (SSP2) and Representative Concentration Pathway 1.9 (RCP1.9) scenario from IMAGE. RCP1.9 represents a pathway limiting global warming to below 1.5 °C by 2100, while SSP2 assumes moderate socioeconomic development^50^ (detailed in Section S1.2).

### 2.5 Model Constraints

PolyLOP minimizes the environmental impact of the plastic industry under several key constraints. For details and the mathematical formulation, see Section S1.6.

#### 2.5.1 Mass and Energy Balance

Conservative availability scenarios of CO_2_-feedstock and lignocellulose residues from our past studies^11,12^ were used as the supply constraint (see Section S1.2.5), but in the sensitivity analysis also, lower and higher availabilities were assessed (see below). We included only those combinations with biodiversity loss impacts below 10^–14^ PDF/kg dry mass (DM), as recommended by Huo et al.^12^ Electricity consumption was constrained by the electricity production forecast from the SSP2-RCP1.9 scenario of IMAGE.^51^

The model ensures that the exogenous plastic demand is met. For all intermediate chemicals, the quantities produced match the quantities consumed in the plastic industry. Additionally, all residual plastic waste that cannot be recycled is incinerated. For global optimization (Figures 1–3), supply and demand constraints were aggregated at the global level (assuming free trade), while for regional optimization (Figures 4 and 5), constraints were applied at the regional level to capture geographic variability in resource availability and demand (i.e., without transborder feedstock/intermediates transport).

#### 2.5.2 Maximum Substitution Rate of Non-Drop-In Plastics

The upper limit of the production volumes of non-drop-in plastics is set according to the technical substitution potential^52^ (Table S23). By considering the comparable properties and applications of conventional plastics, this potential outlines the extent to which various conventional plastics can be replaced by each non-drop-in plastic type.

#### 2.5.3 Maximum Recycling Rates and End-of-Life Treatment

Our model incorporates realistic constraints on plastic recycling rates by taking into account the practical limitations encountered in waste collection, sorting, and the utilization of recycled plastics^16,17,25^ based on Klotz et al.,^17^ which quantified the maximum recyclable plastic waste fraction for both mechanical and chemical recycling for each plastic type projected to 2040. For mechanical recycling, we additionally derive a constraint of the maximum recycled content, acknowledging the limited utilization options of mechanically recycled plastic products.^25^ These constraints were assumed to be the same for all of the regions. For details, see Tables S21 and S22. All nonrecycled plastic waste was assumed to be incinerated.

### 2.6 Scenario Description

The scenarios presented in this study were generated by applying various constraints in the optimization with the objective function of minimizing climate change impacts.

#### 2.6.1 Fossil-Linear Scenario

The fossil-linear scenario serves as an analytical reference by assuming exclusive fossil-based plastic production with waste incineration without any mitigation strategies. As such, it allows for isolating and quantifying the potential contributions of individual mitigation strategies.

#### 2.6.2 Net-Zero Scenario

The net-zero scenario allows for a combination of various strategies, including alternative feedstocks, new production routes, and circular plastics use. Considering the uncertainty of future CCS deployment, we present results both with (default case) and without CCS.

### 2.7 Sensitivity Analysis

The uncertainty of the net-zero scenario was addressed through a sensitivity analysis of key parameters.

#### 2.7.1 Biomass Availability

In the net-zero scenario, the availability of lignocellulose residues was assumed to be 2.3 Gt^12^ (=100%). To account for potential competition for biomass from other sectors as well as higher estimates of availability,^12^ this constraint was varied between 0% and 200% in the sensitivity analysis (detailed in Section S1.7.1).

#### 2.7.2 Electricity Carbon Footprint

The net-zero scenario assumed a global average electricity grid carbon footprint with a decarbonized energy system of 0.07 kg CO_2_-eq/kWh.^45^ Given that this is an ambitious target compared to the current global average, which is more than ten times higher,^53^ a sensitivity analysis was performed by varying the electricity carbon footprint from 0 to 0.4 kg CO_2_-eq/kWh to assess its impact on the climate-optimized plastic industry.

#### 2.7.3 Use of Fossil Resources

While the net-zero scenario did not impose constraints on fossil resource use, two additional scenarios were considered in the sensitivity analysis.

##### 2.7.3.1 Lock-In of Fossil Facilities

A minimum of 265 Mt of plastics must be produced from steam cracking facilities, which have not reached their technical lifetime in 2050 (Section S1.7.2).

##### 2.7.3.2 No Fossil Use

In this sensitivity analysis, natural gas and petroleum consumption was set to zero.

#### 2.7.4 Plastics Production

The net-zero scenario assumed a plastic production of 1 Gt in 2050. To account for uncertainty in future demand, a sensitivity analysis was conducted by applying a production level ranging from 10% to 150% of this baseline projection.

## 3 Results and Discussion

### 3.1 A Net-Zero Plastic Industry Is Feasible but at the Expense of Higher Biodiversity Loss and Health Impacts

In 2050, the fossil-linear scenario would emit at least 4.5 Gt CO_2_-eq at a projected annual production of 1 Gt plastics^9^ (Figure 1a, climate-optimized global result). Several strategies can reduce GHG emissions throughout the plastic industry. First, utilizing alternative feedstocks could reduce emissions by 49%. This reduction mainly comes from biobased drop-in plastics, as many non-drop-in plastics have higher climate change impacts than drop-in alternatives. Given limited biomass resources, optimization favors lower-impact, drop-in plastic production. Second, mechanical and chemical recycling can further reduce emissions by 22% and 8%, respectively. Gasification is selected by the optimization model as the preferred chemical recycling method over pyrolysis (see comparison of the two in Section S2.4). Third, capturing and storing CO_2_ emissions (CCS) from biomass gasification or fermentation is a critical step to achieving negative GHG emissions. Combined, these strategies would yield a global annual GHG emission balance of −0.26 Gt CO_2_-eq—demonstrating the potential for a paradigm shift to a net-zero plastic industry. This net-zero scenario consumes 1.2 Gt of carbon from lignocellulose residues and 0.15 Gt from fossil resources for feedstock and heat (Figure S5). Methanol-to-olefins and methanol-to-aromatics serve as the primary technologies for producing base chemicals, which are subsequently converted into plastics. Notably, the model does not favor CO_2_-based production routes because these have higher climate change impacts than fossil alternatives. These higher impacts are due primarily to the substantial electricity consumption required for hydrogen production—a key reactant in CO_2_ hydrogenation for methanol synthesis, even when the global electricity mix has an average carbon footprint as low as 0.07 kg CO_2_-eq/kWh.

**Figure 1:**
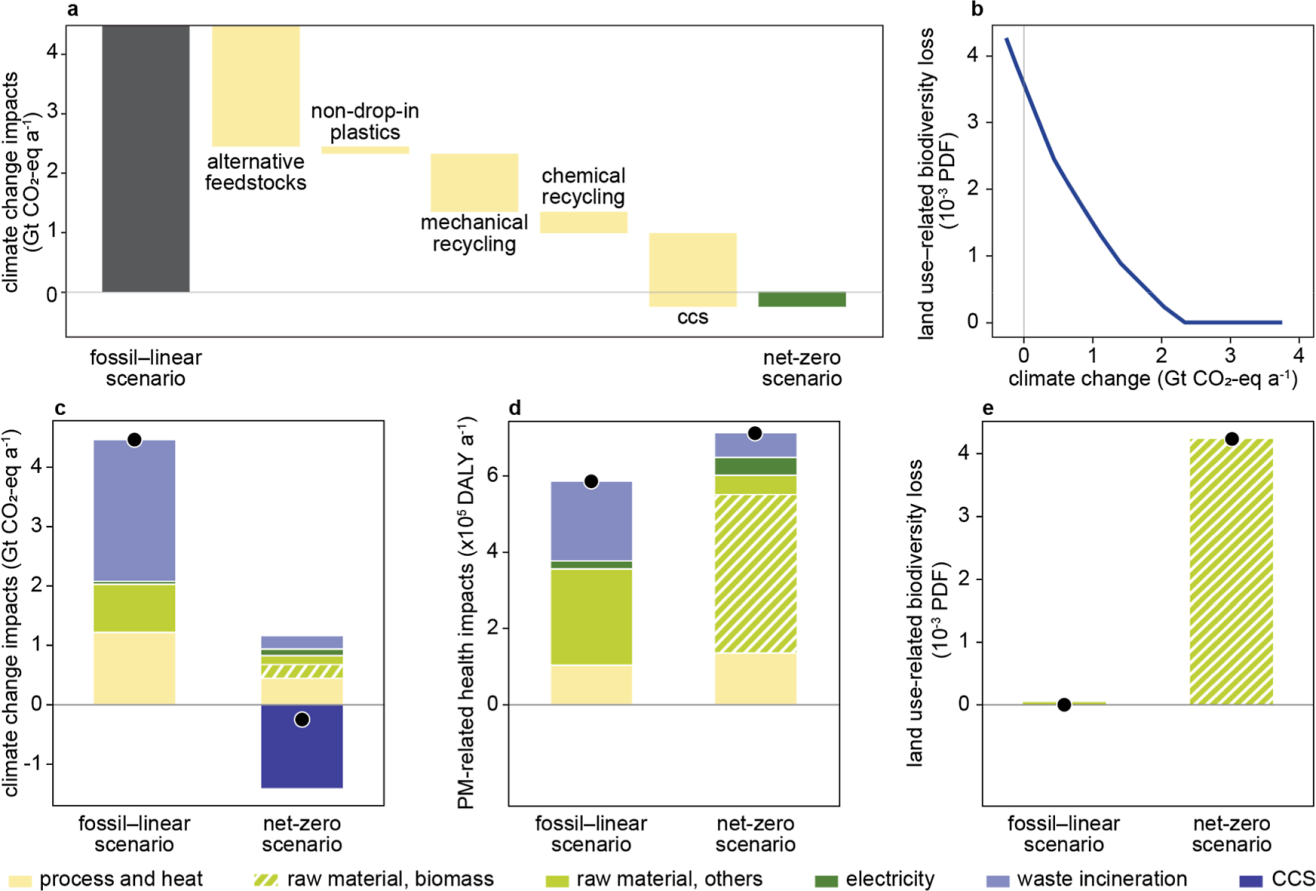
Environmental impacts of global plastics production and end-of-life treatment in 2050. (a) Climate change impacts of a fossil-linear plastic industry and its transition into net zero (climate-optimized scenario). “Alternative feedstocks” include lignocellulose residues and CO_2_ captured from other hard-to-abate sectors. For the results with a different strategy implementation sequence, see Figure S10. (b) Trade-offs between minimizing climate change impacts and land-use-related biodiversity loss of the plastic industry under transition (Pareto front of multiobjective optimization, derived by normalizing climate change impacts and land-use-related biodiversity loss to a −1 to 1 scale and varying their relative weights in the optimization, detailed in Section S1.6). (c–e) Climate change and PM-related health and land-use-related biodiversity loss impact contributions for the fossil-linear scenario and the net-zero scenario. Abbreviations: CCS, carbon capture and storage; PM, particulate matter; DALY, disability-adjusted life years; PDF, potentially disappeared fraction of species.

The climate change impacts of the fossil-linear scenario primarily stem from waste incineration (53%), heat production and process emissions (27%), and fossil extraction and acquisition (18%) (Figure 1c). This baseline scenario is a reference point rather than a typical business-as-usual (BAU) projection of the future plastic industry (for a comparison of the “fossil-linear” baseline scenario with a BAU projection, see Section S2.2). In the net-zero scenario, emissions are mainly attributed to raw material acquisition (33%, of which 61% come from lignocellulose residues), heat production and process emissions (38%), and waste incineration for nonrecyclable plastics (20%). These remaining emissions are compensated through CCS. While landfilling plastic waste might act as carbon sinks, they were excluded from the transition strategies due to the risk of leaching micro- and nanoplastics, as well as harmful monomers, additives, and other chemicals present in the plastic products into the environment.^54,55^ Future studies may be warranted to fully comprehend such trade-offs.

The transition to a net-zero plastic industry introduces trade-offs in the other two dimensions of the triple planetary crisis: pollution and biodiversity loss. The net-zero scenario results in annual PM-related health impacts of 7.1 × 10^5^ DALYs—equivalent to 0.6% of the total global DALY loss due to outdoor air pollution in 2020 ^29^—which is 20% higher than the fossil-linear scenario. While these impacts may appear modest on a global scale, PM pollution affects vulnerable local populations and requires targeted, localized solutions. In the fossil-linear scenario, plastic-waste combustion represents 36% of PM-related health impacts. In the net-zero scenario, however, its contribution diminishes, since plastic waste would be redirected from incineration to maximum possible recycling. Instead, raw materials represent two-thirds of the PM-related health impacts in the net-zero scenario (Figure 1d). These impacts primarily stem from ground-level ammonia emissions resulting from the use of manure and synthetic fertilizers during crop cultivation (Section S2.1.2). Notably, ammonia emissions from agriculture are already the leading cause of PM-related health impacts in Europe today.^56^ This emphasizes the need to reduce agricultural ammonia emissions through, for example, improved manure storage and optimized nitrogen fertilizer application using precision farming techniques.

Along with moving toward reducing climate change impacts, land-use-related biodiversity loss impacts are on the rise because of the increasing use of biomass (see the Pareto curve in Figure 1b based on multiobjective optimization). While the fossil-linear scenario would have minimal impacts on land-use-related biodiversity loss (1.1 × 10^–5^ PDF; uncertainty range: 3.7 × 10^–6^–3.5 × 10^–5^ PDF), in the net-zero scenario, plastics production would put 0.42% of global species at a risk of extinction (4.2 × 10^–3^ PDF, >3800 times more than the fossil-linear scenario, see Figure 1e). The cause of this estimated increase is the increased demand and thus economic value of lignocellulose residues in the net-zero scenario.^12^ Consequently, together with the main products, they would become market drivers of agricultural and forestry practices. Therefore, environmental impacts of biomass cultivation and harvesting were allocated to all coproducts based on their economic values.^12^

### 3.2 Impediments by Limited Sustainable Biomass and Decarbonized Electricity

The net-zero scenario in Figure 1 consumes 2.3 Gt DM lignocellulose residues annually (37 exajoule, or EJ), fully exploiting the available potential under our lower-end estimation^12^—and this number is at the higher end of the range of previous studies (4–43 EJ).^9,14,19,22,23^ Competition from other sectors, such as biofuel production for mobility and energy,^57,58^ may further reduce the availability for plastics production and increase climate change impacts (Figure 2a). At a biomass availability below 2.25 Gt DM, achieving net-zero emissions would become unfeasible (unless CCS is applied beyond high-purity-process CO_2_ emissions). In the net-zero scenario, only 14% of biomass carbon ends up in plastic products, while 32% is captured for storage, and the remainder is emitted from heat production and other processes (Figure S7a).

**Figure 2:**
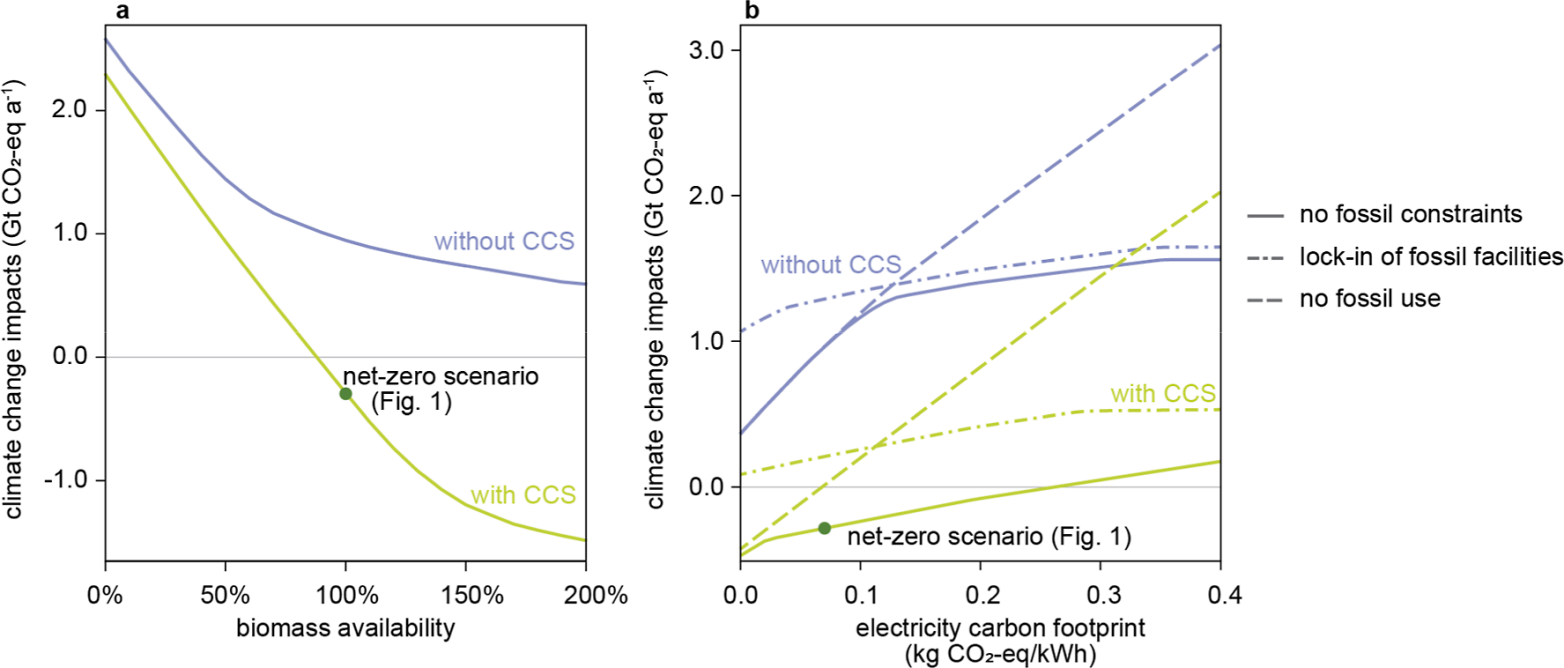
Impact of biomass availability, electricity carbon footprint, and use of fossil resources on the climate change impacts of the optimized future plastic industry. (a) Optimized climate change impacts as a function of lignocellulose-residue biomass availability for the cases without and with CCS. 100% biomass availability represents the baseline of 2.3 Gt DM lignocellulose residues. (b) Optimized climate change impacts as a function of the electricity carbon footprint for the cases without and with CCS under scenario settings that vary in how they use fossil resources. In the scenario “no constraints on fossil use”, there are no additional constraints regarding the use of fossil-based pathways; in the scenario “lock-in of fossil facilities”, at least 265 Mt of fossil-based chemicals are produced from steam crackers; and, in the case “no fossil fuels”, all plastics are produced via alternative feedstocks.

Availability of decarbonized electricity presents another challenge. Under the given assumptions, net zero would require the carbon footprint of the global electricity grid to fall below 0.26 kg CO_2_-eq/kWh (36% of the current global average^53^) (Figure 2b). The net-zero scenario in Figure 1 consumes 1.6 petawatt hour (PWh) annually, 20% higher than the current electricity consumption of the entire chemical industry (1.3 PWh^59^). If the electricity mix decarbonizes further, CO_2_-based production routes that require hydrogen as coreactant would be chosen in our model, dramatically increasing the electricity demand (Section S2.1.3 and Figure S7b). For example, when the electricity carbon footprint approaches zero in a scenario without CCS implementation, annual electricity consumption is projected to reach 9 PWh, which is one-third of the total global electricity production in 2023,^60^ posing a significant challenge.

Fossil resources continue to play a role, even in the optimized climate scenarios, contrary to the assumptions of many previous studies.^14,15,23^ Completely eliminating fossil-based routes is possible only when both sufficient biomass and low-carbon electricity are available. Without these prerequisites, excluding fossil-based routes would necessitate a shift to CO_2_-based production, resulting in higher climate change impacts (Figures 2b and S8).

Additionally, the industry faces a lock-in situation due to recent large investments in steam cracking capacity.^61^ This lock-in constraint of 265 Mt capacity from existing fossil-based facilities would undermine overall transition efforts by emitting an extra 0.35–0.55 Gt of CO_2_-eq annually. The persistence of these fossil-based facilities may create a tension between utilizing existing infrastructure and transitioning to more sustainable production methods. One potential way to partially address such infrastructure lock-in may be the use of bionaphtha (e.g., derived from used cooking oil^62^), which can utilize existing steam crackers to avoid stranded assets. However, further research is needed to quantify the sustainable availability potential of bionaphtha from suitable agricultural and food-processing residues.

### 3.3 Urgency of Reducing the Plastic Demand

The relationship between the volume of plastic production and its associated climate change impacts exhibits a nonlinear pattern (Figure 3a), i.e., the estimated impact per kilogram plastic production increases with production levels. This occurs because the optimization model prioritizes lignocellulose residues with the lowest impacts first. As production increases, these are exhausted, forcing shifts to higher-impact biomass and eventually fossil routes (Figure 3b). Negative emissions reach their lowest point at a global annual plastics production level of 0.65 Gt—35% less plastics than the projected amount for 2050.

**Figure 3:**
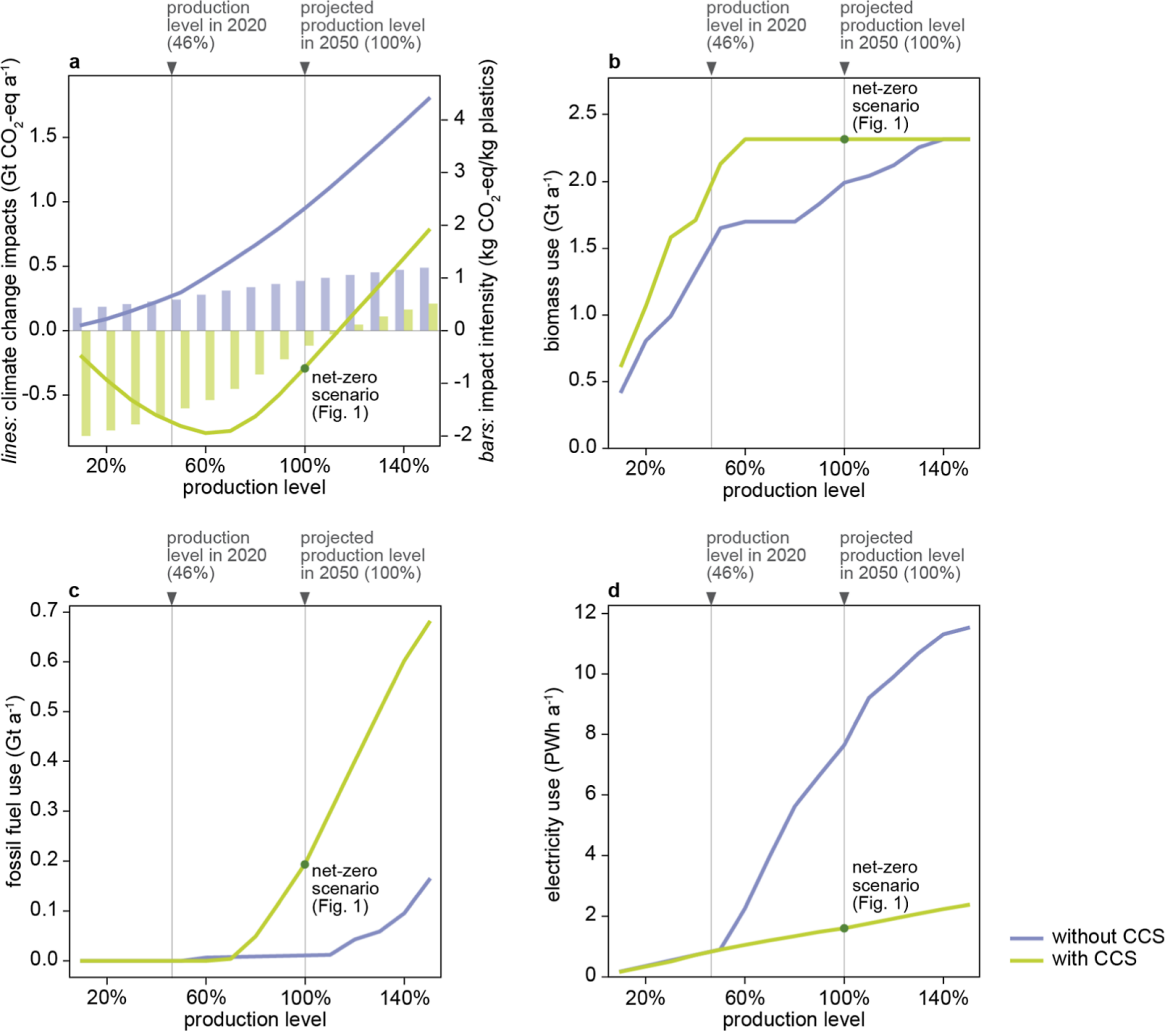
Climate change impacts and resource use as a function of plastics production. (a) Total climate change impacts (left *y*-axis, as lines) and unit climate change impacts per kilogram plastics production (right *y*-axis, as bars). (b) Biomass consumption, including agricultural and forest residues. (c) Fossil fuel consumption, including natural gas and petroleum. (d) Electricity consumption.

When the production is below 70% of the projected demand in 2050, fossil use is unnecessary (Figure 3c). However, due to limited biomass availability, fossil requirements quickly escalate to over 0.6 Gt when the production increases to 140% of the projected demand. The scenario without a CCS implementation shows different resource dynamics. Fewer fossil resources are required due to higher biomass utilization. However, electricity consumption increases significantly, especially when plastics production exceeds 0.5 Gt. As lignocellulose residues become constrained, the model chooses to capture and hydrogenate process CO_2_ (Figure S5), leading to high electricity demand for hydrogen production (Figure 3d). In contrast, when CCS is implemented instead of CCU, the captured CO_2_ is stored rather than hydrogenated, avoiding the sharp increase in electricity use for hydrogen production seen in the scenario without CCS. These findings underscore the high potential to reduce plastic demand and production. Such reduction could be initiated through circular economy strategies focusing on refuse, reduce, and reuse. However, substituting plastic with other materials may often lead to an increase in climate impacts^18^ and therefore demands a careful prior assessment to avoid such backfiring effects.

### 3.4 Varied Regional Climate-Optimal Solutions and Benefits of Global Collaboration

Significant regional differences exist, underscoring that there is no one-size-fits-all approach to implementing sustainable solutions globally. These differences are illustrated by the diverse composition of carbon feedstocks across regions when conducting the optimization at the regional level (for examples, see Figure 4a). Regions with abundant biomass resources find it easier to eliminate their dependence on fossil resources and to reach net-zero emissions. For example, Brazil may leverage its agricultural wealth, sourcing nearly 90% of its plastic feedstocks from agricultural residues. In contrast, regions with limited biomass availability face considerable challenges. The Middle East relies on fossil resources for nearly 50% of its carbon inputs due to scarce biomass resources. Japan, where CO_2_-based routes for plastic production are chosen based on regional optimization model results (15% of its carbon inputs) because of its low-carbon electricity grid and limited biomass availability, presents a unique case. However, the high electricity demand of CO_2_-based production would require 18% of the total projected electricity production in the country in 2050.

**Figure 4:**
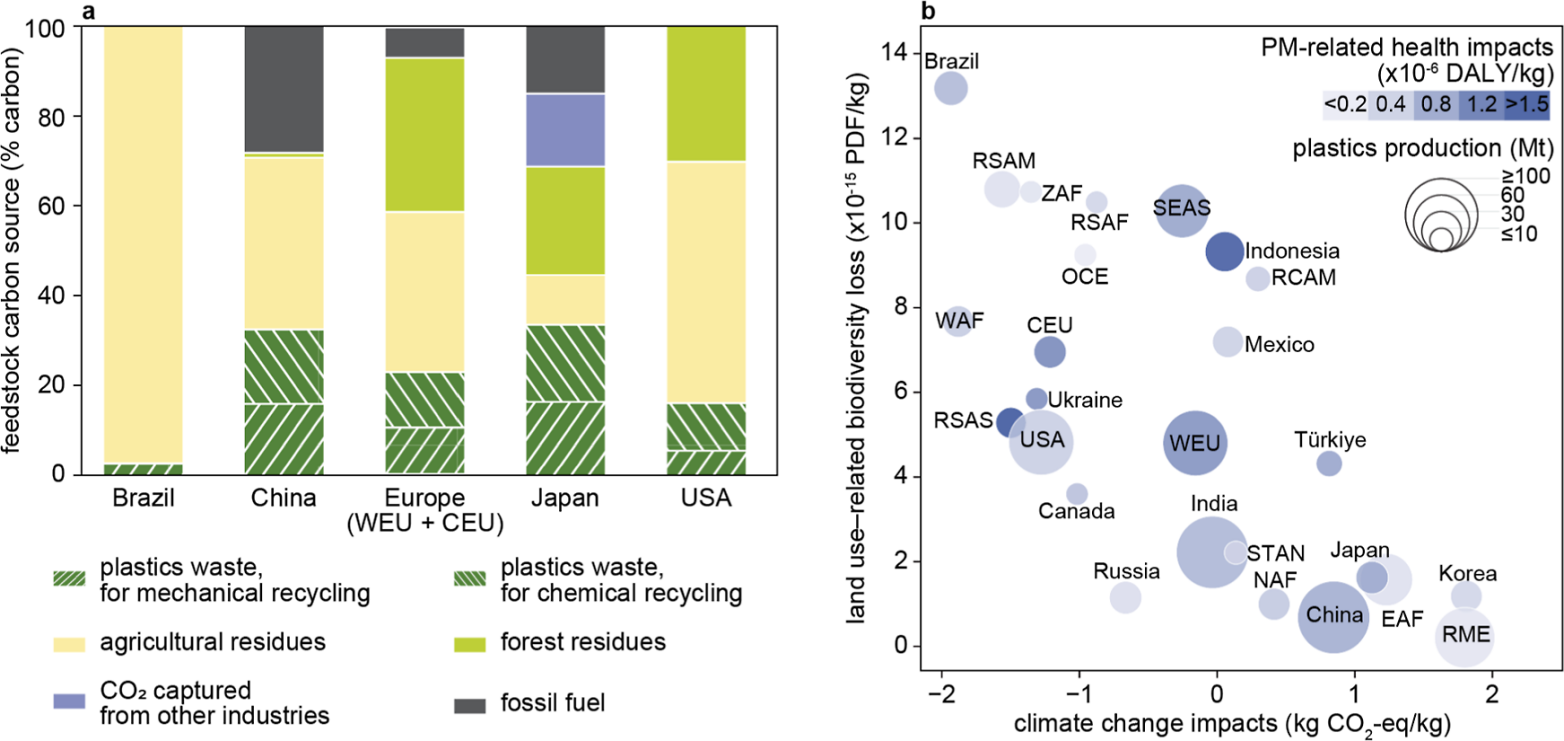
Regionalized solutions and impact intensity of the optimized future plastic industry. (a) The composition of carbon feedstocks in regionally optimized solutions in regions selected as showcases (no feedstock or intermediate chemical trade between nations). (b) Plastic production (circle size), climate change impacts (*x*-axis), land-use-related biodiversity loss impacts (*y*-axis), and PM-related health impacts (different colors) of the regionally optimized plastic industry in various regions. Abbreviations: PM, particulate matter; DALY, disability-adjusted life years; PDF, potentially disappeared fraction of species; EAF, Eastern Africa; NAF, Northern Africa; OCE, Oceania; RCAM, Rest of the Central America; RME, Region Middle East; RSAF, Rest of the Southern Africa; RSAM, Rest of the South America; RSAS, Rest of the Southern Asia; SEAS, Rest of the Southeastern Asia; STAN, Central Asia; USA, United States of America; WAF, Western Africa; WEU, Western Europe; ZAF: South Africa. See Figure S2 for the region definitions.

In a scenario without interregional trade of biomass or the derived intermediate chemicals, where regions must rely solely on locally available feedstock, almost half of the regions struggle to achieve net-zero GHG emissions (Figure 4b), falling short of the global net-zero targets with total emissions of 114 Mt CO_2_-eq annually (Figure 5a). This contrasts with the global optimization that allows for cross-border resource exchange to achieve a global net zero (as in the net-zero scenario in Figure 1). China and the Middle East are major contributors to this excess due to their continued reliance on fossil resources. The disparity between regional and global optimization arises from the imbalance between renewable resource availability and plastic production capacity across regions: some areas with high production capacities lack sufficient low-carbon resources, while others with abundant renewable resources have limited production capacities.

**Figure 5:**
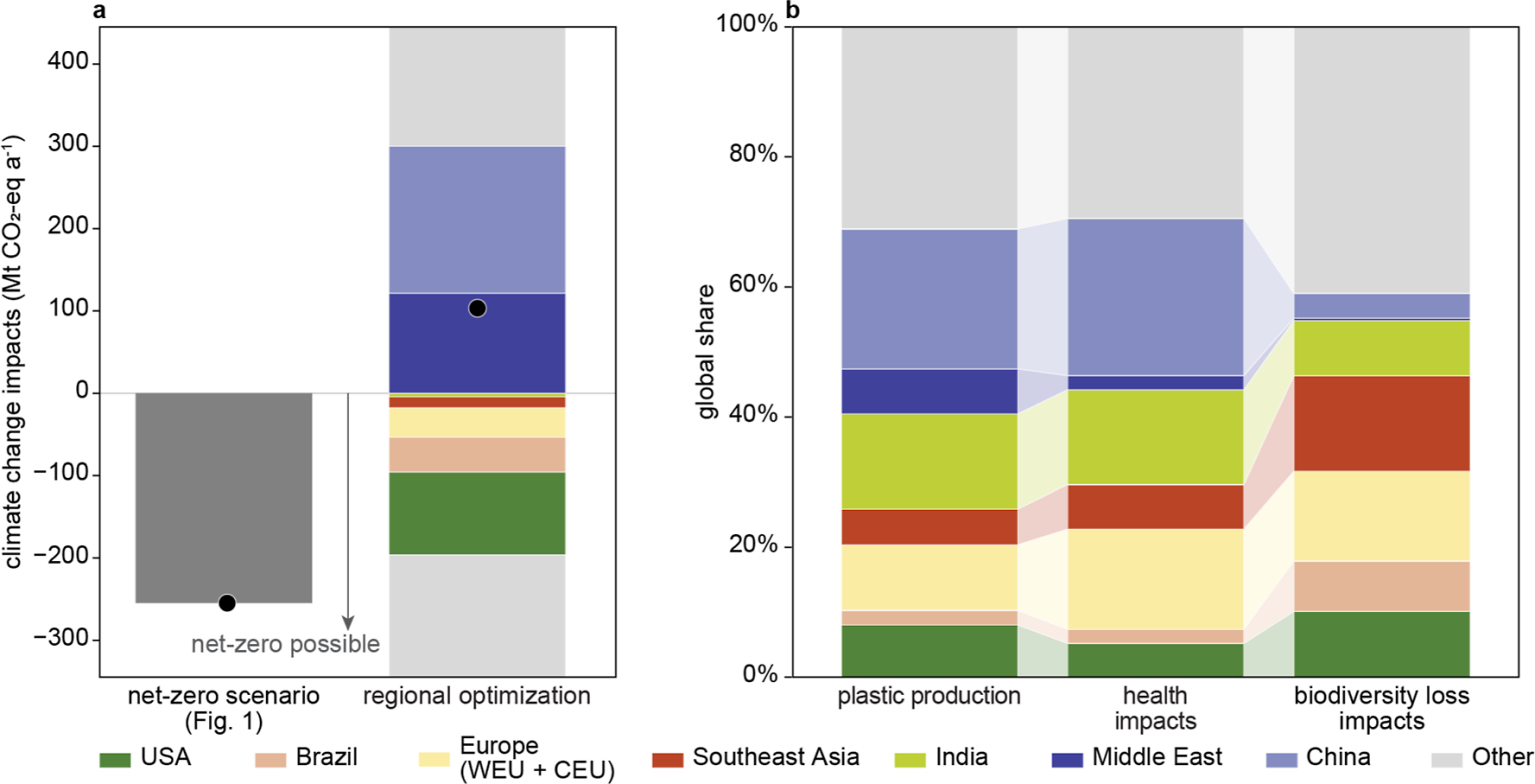
Cumulated impacts of regionally optimized plastic industry. (a) Climate change impacts of regionally optimized plastic industry in comparison to the globally optimized plastic industry. The other countries (in gray) are separated by countries with positive and negative climate change impacts. The aggregated sum is denoted as the black dot. (b) Share of plastic production, health impacts, and biodiversity loss impacts by selected regions of regionally optimized plastic industry. See Figure S2 for the region definitions.

Regional disparities in biodiversity and health impacts per unit of plastic production are profound, with biodiversity loss varying 65-fold and particulate matter-related health impacts varying 93-fold across countries. Brazil faces the highest biodiversity loss impact per unit of plastic production (Figure 4b), as a major share of its carbon feedstock comes from agricultural residues. Southeast Asia emerges as the largest overall contributor to biodiversity loss (Figure 5b), accounting for 15% of global impacts despite producing only 5% of global plastics. This is due to its extensive use of biomass feedstock sourced from vulnerable ecoregions rich in endemic species, even after excluding biomass sources with extreme biodiversity impacts as per Huo et al.^12^ While not situated in particularly vulnerable ecoregions, Europe emerges as the second-largest contributor to biodiversity loss, largely due to the high plastic production amount and relying much more on forest residues as alternative feedstocks for the plastic industry than any other region. Under the net-zero scenario projected for 2050, the intensification of forest management practices in Europe is expected to increase the biodiversity loss impacts associated with forest residues by 4 times compared to current levels.^12^ Conversely, regions such as the Middle East, which rely more on fossil resources and source biomass from less vulnerable ecoregions, exhibit minimal biodiversity loss.

The plastic industry in Indonesia and South Asia has the highest health impacts per unit of production (Figure 4b), mainly because population density is a major driver of health impact per unit of PM emissions.^26^ However, China dominates the overall health impacts with its combination of a high plastic production volume and large amount of exposed population, followed by Europe (Figure 5b). About 15% of the global health impacts are attributed to Europe while producing 10% of the global plastics. Notably, biomass feedstocks are responsible for 76% of PM-related health impacts in Europe (Table S24), primarily from ammonia emissions during crop cultivation.

To summarize, challenges and opportunities in the sustainable transition of the plastic industry vary markedly across regions. The global climate benefits are counterbalanced by regionalized environmental impacts of biodiversity and health impacts occurring especially in biomass-producing regions. The dramatic regional disparities in these two impacts underscore the need for regionally tailored solutions. This study, the first to quantify these variations, demonstrates the importance of sustainable biomass sourcing from nonvulnerable ecoregions and of improved agricultural practices, such as increasing nitrogen use efficiency, to reduce, e.g., ammonia emissions.

### 3.5 Uncertainties and Limitations

The net-zero scenario is subject to further uncertainties beyond the key factors addressed above (biomass availability, electricity carbon footprint, and infrastructure phase-out). One such uncertainty is the climate impact of biogenic CO_2_ from forest residues. Due to the lack of standardized methods, we used a worst-case approach and assumed a clear-cut forest management system and a long average time of 90 years between emission and full sequestering by biomass regrowth, during which period the released CO_2_ would contribute to global warming (i.e., GWP_bio_ = 0.38). Also, due to lack of data on the country-specific rotation period of forests, the GWP_bio_ factor was not regionalized. However, a sensitivity analysis assuming no climate impact from forest residue biogenic CO_2_ was performed (i.e., GWP_bio_ = 0). It shows a decrease in climate change impacts of the net-zero scenario to −0.57 Gt CO_2_-eq (Figure S11). For forests with shorter rotation periods or alternative management systems, the climate change impacts of forest residues would likely fall between these two extremes.

Another uncertainty concerns allocation methods in LCA. For processes with coproducts, we use economic allocation by default, distributing impacts based on economic value. In a sensitivity analysis, we applied system expansion by substitution. For example, 1 molar (MJ) of coproduced heat is assumed to offset 1 MJ of heat from biomass or natural gas, depending on model choices. While the overall conclusions remain the same, the total climate impact would decrease by 0.1 Gt CO_2_-eq compared to economic allocation, mainly due to credits from heat coproducts (Figure S12).

One key study limitation is that we were not able to account for all impact drivers associated with plastics, such as oil spill incidents that may occur during fossil-based production routes, or the effects of microplastics, additives, and other plastic chemicals on biodiversity and human health.^63–65^ Other limitations include the selection of processes and technologies, while development of improved production routes, materials, feedstocks, and plastic waste treatment facilities may further lower environmental impacts. The users of PolyLOP, however, may import user-defined inventory data for additional processes to expand the analysis to new developments. Furthermore, costly mitigation routes might be chosen by our model, as we did not consider economic constraints. Similarly, we assumed an ideal global supply chain without accounting for the economic constraints and logistical challenges associated with biomass transportation, which could affect the feasibility and environmental impacts of the proposed solutions. In addition, we used prospective life cycle inventories in projecting the impacts in 2050, but characterization factors may change as well due to future changes in ecosystems, background pollutant levels, or population density. Additionally, while we assessed biogenic carbon emissions, we neglected potential temporal carbon storage benefits in the plastic products due to missing information about product lifetimes. Finally, while our analysis addresses several key drivers of the triple planetary crisis, it primarily focuses on their direct impacts and ignores their interdependencies. Due to substantial uncertainties and methodological limitations, we refrained from quantifying the complex interactions among these drivers, including how climate impacts affect biodiversity and human health. These interlinkages merit further investigation in future research.

While this paper outlines potential pathways for more sustainable plastics production, it does not explore socioeconomic conditions for incentivizing these developments. For example, supportive regulatory frameworks are critical as stringent environmental policies can incentivize low-carbon feedstock adoption. Meanwhile, differing regulatory landscapes across regions may pose additional challenges to sustainable transitions, as regulatory arbitrage could lead to industrial relocation toward regions with weaker environmental governance. International coordination and harmonization of environmental governance are needed to facilitate efficient global resource allocation while preventing displacement of environmental burdens to less regulated areas.

More details about the study limitations are available in Section S3.

## 4 Implications

Our model advances previous research by including several key drivers of the triple planetary crisis—climate change, land-use-related biodiversity loss, and PM-related human health impacts—of the global plastic industry. By incorporating spatially explicit data on plastic production capacities, as well as alternative feedstock availability and environmental impacts, this study is the first to capture the massive regional disparities in health and biodiversity impacts of plastics by considering regional variations of ecosystem vulnerabilities, population densities, and pollutant concentrations that influence net-zero pathways and their broader environmental implications. Additionally, the model provides a realistic assessment of future challenges compared to previous studies that either compare technologies for a single product without considering constraints and upscaled implications on the global level or based on unrealistic scales of mitigation technologies without accounting for their practical constraints. We reveal the following crucial insights into the transition to a net-zero global plastic industry.

Achieving a net-zero plastic industry is possible through a combination of strategies. However, while a net-zero industry would address climate change, it would involve trade-offs in land-use-related biodiversity loss and PM-related health impacts, which should be addressed regionally by avoiding biomass sources with high biodiversity loss impacts and by mitigating ammonia emissions from agriculture (e.g., with precision agriculture).

Also, achieving this transition requires addressing several fundamental challenges, including limitations in biomass availability, low-carbon electricity, and the rapid upscaling of CCS and plastic waste recycling deployment. Additionally, the early phase-out of existing fossil-based infrastructure may add to the short-term economic burden of the net-zero transition but may bring long-term climate benefits. Based on our findings, we propose several recommendations for transitioning toward a sustainable plastic industry.

First, reducing plastic demand is crucial for mitigating climate change impacts and resource stress. Demand reduction can be achieved by extending product lifetimes, increasing product reuse, and minimizing obsolescence. However, any material substitution should be preceded by careful assessments to ensure a positive environmental outcome. Therefore, how and the extent to which plastic demand can be reduced remain important areas for future research.

Second, a net-zero transition needs careful coordination between the phase-out of fossil fuels in the plastic industry and the decarbonization of the electricity grid. A staged approach in which the reduction of fossil use in plastic production is aligned with an increasing availability of low-carbon electricity is necessary. In regions where grid decarbonization is progressing, the earlier retirement of steam crackers should be encouraged.

Third, regional variations in resource availability, technological capabilities, and environmental impacts require region-tailored approaches. In scenarios optimized for global climate benefits, some countries bear greater biodiversity losses and health burdens. This uneven distribution of regional biodiversity and health impacts, while contributing to global climate benefits, highlights the need for a just global transition that may be considered under the framework of the Global Plastics Treaty.^66^

Fourth, international collaboration is a prerequisite for reaching net zero in the plastic industry. Coordinated relocation of plastic production facilities and trade in low-impact feedstocks or the derived intermediate chemicals may be ways to optimize resource utilization and minimize environmental impacts on a global scale.

In summary, the path forward requires plastics-demand reduction, expanding recycling and alternative feedstock production, strategically phasing out fossil fuels, and deploying CCS. Global collaboration is crucial to drive this extremely challenging transition effectively. On top of the net-zero challenges come increased biodiversity and health effects. While no scenario solves all dimensions of the triple planetary crisis, our study illuminates the trade-offs between local and global environmental benefits and impacts, enabling informed decision-making and targeted mitigation strategies.

## Supplementary Material





## Data Availability

Codes for PolyLOP can be accessed in the GitHub repository: https://github.com/ecological-systems-design/polyLOP

## Notes

Details on methods and additional results and discussions (PDF)
Production pathway choices for a plastic industry with minimum climate change impacts for 26 world regions and globally (XLSX)
