# Supplementary material for: Global Plastic Industry Transition Addressing Key Drivers of the Triple Planetary Crisis
Source: Environ Sci Technol. 2025 Sep 2;59(36):19257–68. doi: 10.1021/acs.est.5c08703 (PMC12444996; doi:10.1021/acs.est.5c08703)
Supplement: Supplementary file 1 [file es5c08703_si_001.pdf]

Supporting Information

Global plastic industry transition addressing key drivers of the triple planetary crisis

Jing Huo*[a, b], Zhanyun Wang[b, c], Christopher Oberschelp[a, b], and Stefanie Hellweg[a, b]

Author Information:

[a] Chair of Ecological Systems Design, Institute of Environmental Engineering, ETH Zürich, Zürich, Switzerland

[b] National Centre of Competence in Research (NCCR) Catalysis, ETH Zürich, Zürich, Switzerland

[c] Swiss Federal Laboratories for Materials Science and Technology (Empa), Technology and Society Laboratory, St. Gallen, Switzerland

Correspondence and requests for materials should be addressed to Jing Huo: jhuo@ethz.ch

This supporting information contains 75 pages, 12 figures, and 25 tables.

Contents

S1 Supplementary methods **S5**

S1.1 Study overview . . . S5

S1.2 Overarching settings . . . S5

S1.2.1 IMAGE integrated assessment model scenario . . . S5

S1.2.2 Plastic types and applications . . . S6

S1.2.3 Share of plastic types within each subsector–data harmonization . . S8

S1.2.4 Plastic waste amount . . . S11

S1.2.5 Alternative carbon feedstock . . . S11

S1.3 Processes included in the model . . . S12

S1.3.1 Fossil-based plastics production . . . S13

S1.3.2 Alternative feedstock-based plastics production . . . S16

S1.3.3 Plastic waste treatment . . . S18

S1.3.4 Storage of captured CO_2 . . . S21

S1.3.5 Utilities . . . S21

S1.4 Life cycle inventory data . . . S21

S1.4.1 Background data . . . S21

S1.4.2 Allocation . . . S23

S1.4.3 Particulate matter-related emissions from biomass combustion and plastic waste incineration . . . S23

S1.4.4 N_2O emissions from adipic acid production . . . S23

S1.4.5 Gate-to-gate inventories based on the Process Economics Program (PEP) Yearbook from IHSMarkit . . . S23

S1.4.6 Gate-to-gate inventories based on life-cycle inventories . . . S26

S1.4.7 Gate-to-gate inventories based on other sources . . . S30

S1.5 Impact assessment . . . S39

S1.5.1 Climate change impacts of biogenic CO_2 emissions . . . S39

S1.5.2 Land use–related biodiversity loss impacts . . . S39

S1.6 Linear optimization model: mathematical formulation . . . S40

S1.6.1 Objective Function . . . S40

S1.6.2 Constraints . . . S41

S1.7 Sensitivity analysis . . . S45

S1.7.1 Biomass availability . . . S45

S1.7.2 Lock-in of fossil facilities . . . S45

S2 Supplementary results **S47**

S2.1 Supplementary results S47

S2.1.1 Carbon flow for the plastic industry S47

S2.1.2 Ammonia emissions from agricultural residues S48

S2.1.3 Resource constraints S50

S2.2 Comparison with previous studies: climate change impacts of plastics . . . S58

S2.3 Comparison with previous studies: net-zero transition of the plastics industryS59

S2.4 Sensitivity analysis: gasification vs pyrolysis for chemical recycling S61

S2.5 Sensitivity analysis: without constaints on biodiversity loss S61

S2.6 Sensitivity analysis: impact of strategy implementation sequence S63

S2.7 Sensitivity analysis: climate change impact of biogenic CO_2 . . . . S63

S2.8 Sensitivity analysis: allocation method S64

S3 Study limitations **S66**

S4 References **S69**

List of Figures

S1 Study overview. S5

S2 26 regions in the IMAGE model. S6

S3 Major plastic production processes included in the model S13

S4 Minimum fossil-based plastic production capacity projection S46

S5 Carbon flow for the plastic industry in 2050 with minimum climate change impacts. S47

S6 Cradle-to-gate ammonia emissions of lignocellulose residues under the RCP1.9 scenario in 2050 . . . . S49

S7 Impact of biomass availability and electricity carbon footprint on the climate change impacts of the optimized future plastic industry S51

S8 Minimum climate change impacts of the global plastic industry in 2050 as a function of electricity carbon footprint and biomass availability under different constraints regarding fossil use. S52

S9 Sensitivity analysis of relative impacts, for the net-zero scenario without constraint on biodiversity loss impacts S62

S10 The climate change impacts of a fossil linear plastics industry and its transition into a net-zero one, with a different sequence of applying strategiesS63

S11 Climate change impacts of the net-zero plastics industry with two different accountings of the $GWP100_{bio}$ S64
S12 Climate change impacts of the net-zero plastics industry with two allocation methods S65

List of Tables

S1 Plastic types in the model S7
S2 Plastic application sectors and subsectors S7
S3 Share of plastic types within each sector (100 % refers to each sector total) S10
S4 Global plastics production by plastic type and subsector in 2050 . . . . . S10
S5 Fossil-based plastics production processes S14
S6 Alternative feedstock-based drop-in plastics production processes S16
S7 Alternative feedstock-based non-drop-in plastics production processes . . . S17
S8 Potential replacement of conventional plastics with non-drop-in alternatives and their substitution factors by application within subsectors S17
S9 Plastic waste treatment processes S19
S10 CO_2 storage processes S21
S11 Utility processes S21
S12 System raw materials S22
S13 Gas density under normal temperature and pressure S24
S14 Lower Calorific Values for fuels S25
S15 GHG emissions of fuel combustion S25
S16 PM-related emissions of fuel combustion S25
S17 Additional emissions added to PEP-derived processes S26
S18 ecoinvent-derived processes S26
S19 Agrifootprint-derived processes S30
S20 Gate-to-gate life-cycle inventories of processes derived from other sources . S31
S21 Maximum end-of-life recycling rate and recycled content for mechanical recycling S43
S22 Maximum end-of-life recycling rate for chemical recycling S43
S23 Maximum substitution rate of non-drop-in plastics S45
S24 Environmental impacts of regionally optimized plastic industry, by region and impact source S53
S25 Comparison with previous studies S59

S1 Supplementary methods

S1.1 Study overview

The system boundary of this study encompasses the cradle-to-gate life cycle stages of plastics, including feedstock extraction/production, chemical and plastic production processes, and end-of-life treatment options (incineration, chemical recycling via gasification and pyroylisis, and mechanical recycling). The transportation is excluded from the system boundary because the locations of future biorefineries are unknown, making it impossible to accurately model transportation distances and impacts. The use phase is also excluded from the analysis. This exclusion is expected to have minimal impact on the comparison across scenarios, as use phase impacts are likely to remain consistent across all scenarios regardless of the feedstock origin or production pathway.

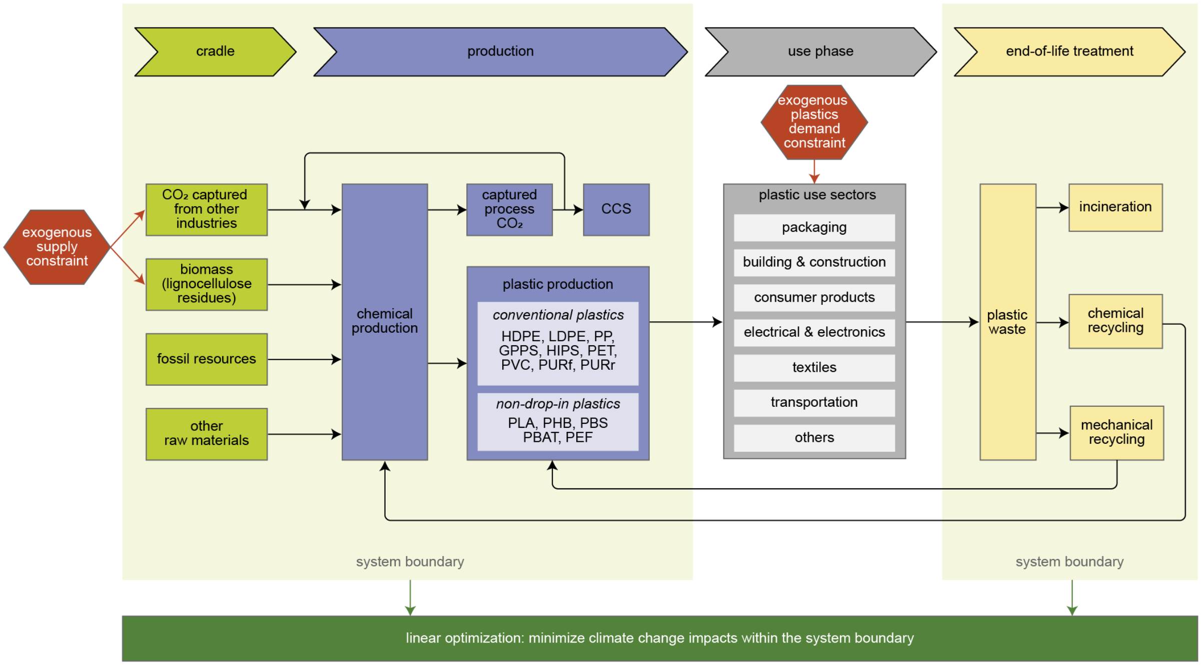

Figure S1: Study overview. For detailed process overview of chemical and plastic production, see Figure S3

S1.2 Overarching settings

S1.2.1 IMAGE integrated assessment model scenario

The following data are derived from the IMAGE integrated assessment model[1]. Particularly, the default SSP2-RCP1.9 scenario is used. It is a climate scenario that combines a "middle of the road" socioeconomic pathway (SSP2) with very stringent greenhouse gas (GHG) mitigation efforts (RCP1.9). This scenario assumes moderate challenges to mitigation and

adaptation, along with ambitious emissions reductions aimed at limiting global warming to below 1.5 °C above pre-industrial levels by the end of the century.

- Projected plastics production from 2020 to 2050 in 26 world regions (Figure S2).
- Projected electricity generation in 2050 in 26 world regions.
- The SSP2-RCP1.9 IMAGE scenario is coupled with ecoinvent 3.8 (cut-off system model) to generate prospective background life-cycle inventories for 2050 (see Section S1.4.1).

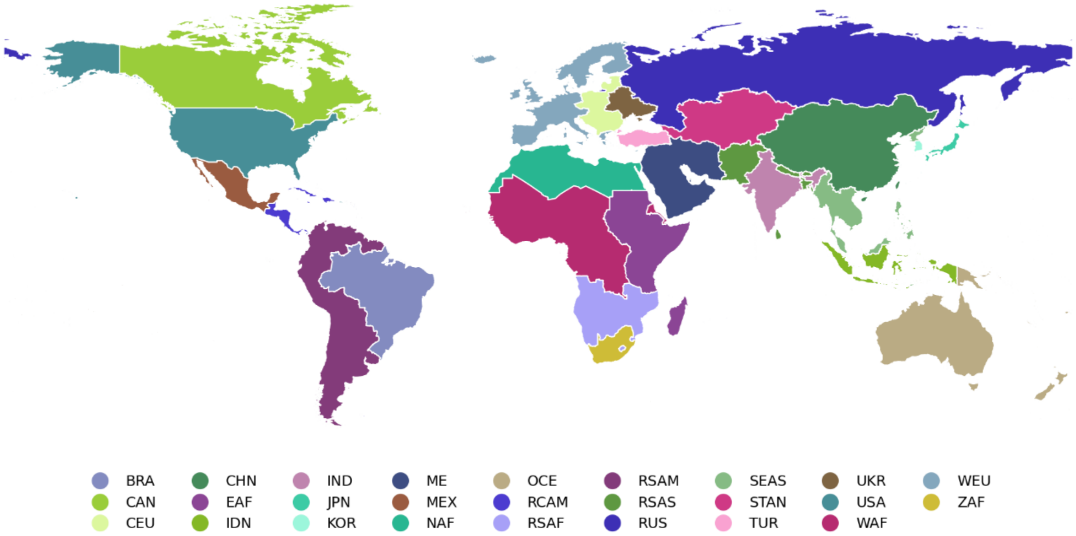

Figure S2: 26 regions in the IMAGE model. The countries allocated to each regions are documented in the model description.[2] Region abbreviations: BRA, Brazil; CAN, Canada; CEU, Central Europe; CHN, China region; EAF, Eastern Africa; IND, India; IDN, Indonesia region; JAP, Japan; KOR, Korea region; MEX, Mexico; NAF, Northern Africa; OCE, Oceania; RCAM, Rest of Central America; RME, Region Middle East; RSAF, Rest of Southern Africa; RSAM, Rest of South America; RSAS, Rest of South Asia; RUS, Russia region; SEAS, Rest of South Eastern Asia; STAN, Central Asia; TUR, Türkiye; UKR, Ukraine region; USA, United States of America; WAF, Western Africa; WEU, Western Europe; ZAF, South Africa.

S1.2.2 Plastic types and applications

Table S1: Plastic types in the model

Full name	Abbreviation	Density ($kg\,m^{-3}$)	Carbon content	Type
High-density polyethylene	HDPE	953[3]	86%	Conventional plastics
Low-density polyethylene[i]	LDPE	920[3]	86%	Conventional plastics
Polypropylene	PP	908[4]	86%	Conventional plastics
Polystyrene, general purpose	GPPS	1050[5]	92%	Conventional plastics
Polystyrene, high impact	HIPS	1045[5]	92%	Conventional plastics
Polyethylene terephthalate	PET	1370[6]	63%	Conventional plastics
Polyvinyl chloride	PVC	1380[5]	38%	Conventional plastics
Polyurethane, rigid	PURr	50–1700[7]	63%	Conventional plastics
Polyurethane, flexible	PURf	50–1700[7]	61%	Conventional plastics
Polylactic acid	PLA	1240[8]	50%	Non-drop-in plastics
Polybutylene succinate	PBS	1250[9]	56%	Non-drop-in plastics
Polyethylene furanoate	PEF	1428[10]	53%	Non-drop-in plastics
Polyhydroxybutyrate	PHB	1250[11]	56%	Non-drop-in plastics
Polybutylene adipate terephthalate	PBAT	1260[8]	63%	Non-drop-in plastics

Table S2: Plastic application sectors and subsectors

Harmonized subsectors in this study	Sectors as in Stegmann et al.[12] and Geyer et al.[13]	Sectors as in Klotz et al.[14]	Subsectors as in Klotz et al.[14]
Buildings and construction	Buildings & Construction	Building and Construction	All
Household items	Consumer Products	Household items, furniture, leisure and others	Household items
Medical and hygiene items	Consumer Products	Household items, furniture, leisure and others	Medical and hygiene items
Rest of consumer products	Consumer Products	Household items, furniture, leisure and others	All other
Electrical and electronic	Electrical & Electronic (products)	Electrical and Electronic Equipment	All
Mulch films	Other	Agriculture	Agricultural films - mulch
Rest of other	Other	Industrial Machinery	All other

Continued on next page

[i]including linear low-density polyethylene (LLDPE)

Table S2 continued

Harmonized subsectors in this study	Sectors as in Stegmann et al.[12] and Geyer et al.[13]	Sectors as in Klotz et al.[14]	Subsectors as in Klotz et al.[14]
		Agriculture	
Food films	Packaging	Packaging	Food films, Non-consumer packaging C&I - hospitality - films
Non-food films	Packaging	Packaging	Consumer non-food films
Consumer non-food bags	Packaging	Packaging	Consumer non-food bags
Beverage bottles	Packaging	Packaging	Food bottles, Non-consumer packaging C&I - hospitality - bottles
Containers for food	Packaging	Packaging	Food PTTs, Non-consumer packaging C&I - hospitality - PTTs
Containers for non-food	Packaging	Packaging	Consumer non-food PTTs
Non-consumer packaging, rigids	Packaging	Packaging	Non-consumer packaging C&I - manufacturing - rigids
Other food packaging	Packaging	Packaging	Food other
Rest of packaging	Packaging	Packaging	All other
Textiles	Textiles	Textiles	All
Transportation	Transportation	Automotive	All

S1.2.3 Share of plastic types within each subsector–data harmonization

The initial dataset is derived from Geyer et al.[13], which provides information on the global production share of each plastic type across various sectors. To combine this data with the plastics production forecast by sector from Stegmann et al.[12], the distribution of plastic types within each sector is normalized. This normalization involved recalculating the percentages of each plastic type so that their sum of share within each sector equaled 100 %. Given that Geyer's dataset did not cover the textile sector, this is supplemented with data from Klotz et al.[15]

We further detail the distribution of specific plastics by subdividing polystyrene (PS) into GPPS and HIPS. The subdivision is based on their relative proportions used in each sector, according to comprehensive use data specific to Switzerland.[15] Similarly, polyurethane is divided into flexible PUR (PURf) and rigid PUR (PURr), with differentiation based on their chemical constituents and applications: per kg flexible PUR production requires 0.39 kg of Toluene Diisocyanate (TDI), and per kg rigid PUR requires 0.64 kg of Methylene Diphenyl Diisocyanate (MDI).[16] Based on the forecasted global production of 8.7 million

tonnes (MT) of MDI and 3.3 MT of TDI for the year 2025[17], predominantly used in the PUR market[18], we calculate the production share ratio of rigid PUR to flexible PUR as 1.95 : 1. This ratio is uniformly applied across all sectors to maintain consistency in our modeling.

We further refine our analysis for sectors where non-drop-in plastics could potentially replace conventional plastics. For each of these sectors, we break them down into more specific subsectors using data from Klotz et al.[15] These subsectors are listed in Table S2 (Column "Harmonized subsectors in this study". Take the packaging sector as an example:

- Subsectors within the packaging sector where non-drop-in plastics can play a role are identified, such as "Food films", "Non-food films", "Consumer non-food bags", "Beverage bottles", "Containers for food", "Containers for non-food", and "Other food packaging".
- For each subsector, its share of the total packaging sector is determined. For instance, food films make up 8 % of the total packaging sector, according to Klotz et al.[15]
- The share of each plastic type used within each subsector is calculated, based on data from Klotz et al.[15] These shares are rescaled to match the subsector's total share of the packaging sector. For example, the row for the "Food films" subsector in Table S3 sums to 8 %.
- After accounting for all identified subsectors, the plastic type shares for the remaining "Rest of packaging" category are recalculated. For example:
  - If 21 % of plastics used in the packaging sector is HDPE (based on Geyer et al.[13]),
  - And the sum of HDPE used in all the identified subsectors is 9 %,
  - Then the share of HDPE in the "Rest of packaging" category would be 12 % (21 % − 9 % = 12 %).
- In some cases, this recalculation resulted in negative shares for certain plastic types in the "Rest of packaging" category, due to data inconsistencies between Geyer et al.[13] and Klotz et al.[15] When this occurred, 0 % is assigned to that plastic type for the "Rest of packaging" category and rescaled the shares for the whole sector to ensure they summed to 100 %.

The harmonized data can be found in Table S3. These market shares were assumed to remain constant across regions and through 2050. As some specialty plastic types are not covered in this study, 1 Gt plastics production in 2050 was used as the baseline demand and production amount–92 % of the production projection according to Stegmann et al.[12]

Table S3: Share of plastic types within each sector (100 % refers to each sector total)

Sector	Subsector	HDPE	LDPE	PET	PP	GPPS	PVC	HIPS	PURf	PURr
Buildings and Construction	Buildings and construction	18 %	6 %	0 %	6 %	12 %	43 %	0 %	4 %	8 %
Consumer Products	Medical and hygiene items	1 %	1 %	0 %	1 %	1 %	1 %	0 %	2 %	3 %
Consumer Products	Rest of consumer products	11 %	18 %	1 %	24 %	11 %	3 %	1 %	4 %	8 %
Electrical and electronic	Electrical and electronic	5 %	12 %	0 %	21 %	5 %	9 %	16 %	3 %	6 %
Other	Mulch films	0 %	5 %	0 %	0 %	0 %	0 %	0 %	0 %	0 %
Other	Rest of other	7 %	8 %	0 %	32 %	4 %	10 %	0 %	7 %	13 %
Packaging	Food films	1 %	3 %	0 %	4 %	0 %	0 %	0 %	0 %	0 %
Packaging	Non-food films	1 %	2 %	0 %	3 %	0 %	0 %	0 %	0 %	0 %
Packaging	Consumer non-food bags	0 %	0 %	0 %	0 %	0 %	0 %	0 %	0 %	0 %
Packaging	Beverage bottles	3 %	0 %	11 %	1 %	0 %	0 %	0 %	0 %	0 %
Packaging	Containers for food	0 %	0 %	2 %	3 %	2 %	1 %	0 %	0 %	0 %
Packaging	Containers for non-food	0 %	0 %	2 %	2 %	1 %	0 %	0 %	0 %	0 %
Packaging	Other food packaging	1 %	0 %	1 %	4 %	0 %	0 %	0 %	0 %	0 %
Packaging	Rest of packaging	12 %	21 %	4 %	5 %	5 %	1 %	0 %	0 %	0 %
Textiles	Textiles	2 %	0 %	53 %	26 %	0 %	0 %	0 %	0 %	0 %
Transportation	Transportation	12 %	1 %	0 %	38 %	0 %	4 %	0 %	8 %	16 %

Table S4: Global plastics production by plastic type and subsector in 2050

Unit: Mt plastics										
Subsector	HDPE	LDPE	PET	PP	GPPS	PVC	HIPS	PURf	PURr	Other[ii]
Buildings and construction	31.6	10.5	0.0	11.5	21.1	77.6	0.0	7.8	15.2	4.8
Medical and hygiene items	0.6	1.7	0.1	1.7	0.7	1.2	0.0	2.0	3.9	2.3
Rest of consumer products	12.7	21.1	0.6	28.2	12.7	3.5	0.7	5.0	9.7	7.2

Continued on next page

Table S4 continued

Unit: Mt plastics										
Subsector	HDPE	LDPE	PET	PP	GPPS	PVC	HIPS	PURf	PURr	Other[ii]
Electrical and electronic	2.2	5.5	0.0	10.0	2.5	4.4	7.8	1.5	2.9	11.1
Mulch films	0.0	7.1	0.0	0.0	0.0	0.0	0.0	0.0	0.0	0.0
Rest of other	9.8	11.6	0.0	43.2	6.5	13.7	0.4	9.3	18.2	16.7
Food films	4.5	12.8	1.9	16.6	0.7	1.4	0.0	0.0	0.0	0.0
Non-food films	3.3	7.1	1.2	11.3	0.5	1.1	0.0	0.0	0.0	0.0
Consumer non-food bags	0.8	0.6	0.0	0.0	0.0	0.0	0.0	0.0	0.0	0.0
Beverage bottles	11.1	0.0	44.2	2.4	0.0	0.0	0.0	0.0	0.0	0.0
Containers for food	1.2	0.1	9.0	12.2	9.6	2.2	0.0	0.0	0.0	0.0
Containers for non-food	0.4	0.1	6.8	8.8	6.9	1.6	0.0	0.0	0.0	0.0
Other food packaging	4.3	2.0	2.7	14.7	2.3	0.4	0.0	0.0	0.0	0.0
Rest of packaging	49.3	86.2	15.7	21.1	18.0	2.2	0.0	0.5	1.1	0.8
Textiles	2.4	0.0	71.5	35.2	0.0	0.0	0.0	0.0	0.0	26.3
Transportation	8.7	1.1	0.0	28.4	0.0	3.3	0.0	5.9	11.5	15.3

S1.2.4 Plastic waste amount

Waste plastics calculations were based on 2020 plastics production volumes, projections for 2050, and the average sector-specific lifetime of plastic products from Klotz et al.[15] Specifically, the volume of waste plastics for a sector in 2050 was calculated by determining the production year (2050 minus average lifetime) and then matching the corresponding production amount by interpolating between 2020 and 2050 data or extrapolating for pre-2020 production. For instance, for a sector where plastics had an average lifetime of 5 years, the waste plastics in 2050 was assumed to equal the production amount in 2045. The production amount for 2045 was then interpolated from the known 2020 production volume and the projected 2050 production volume.

S1.2.5 Alternative carbon feedstock

CO_2 captured from industrial point sources Four key industrial sectors—power plants, cement kilns, steel mills, and kraft pulp mills—represent main sources of CO_2

[ii] "Other" represents 8 % of total plastics production and is not included in this study.

feedstock, with global supply potential estimated at 5.2 Gt annually by 2050 under net-zero scenarios.[19] This conservative estimate accounts for the projected decarbonization pathways of these sectors, including electricity grid decarbonization and implementation of emission reduction technologies. These point sources were prioritized over direct air capture in this study due to their higher CO_2 concentrations in flue gas streams, lower energy requirements for capture, and greater near-term technological and economic feasibility.

Lignocellulose residues Agricultural and forest residues represent the key sources of lignocellulose feedstock and were considered as the alternative carbon feedstock for the plastic industry. Specifically, agricultural residues include eight major harvest residue types (barley straw, maize straw, rapeseed straw, rice straw, sorghum straw, soybean straw, sugarcane tops and leaves, and wheat straw), selected based on crop types with the highest global production volumes. Based on Huo et al.[20], the theoretical potential of lignocellulose residues was calculated by multiplying crop production volumes from GLOBIOM projections with crop-specific residue-to-product ratios. The ecological potential was then derived by applying constraints including soil protection requirements (2.5 tonnes/ha left in soil). The available potential was calculated as 70% of the ecological potential. Under the lower-end estimation and by eliminating biomass with high biodiversity impacts, the global available potential of lignocellulose residues is projected to reach 2.3 Gt/year by 2050.[20] The focus on lignocellulose residues, rather than other primary biomass types such as food crops, is to avoid concerns about food security due to potential competition for land, and potential deforestation driven by cropland expansion.

S1.3 Processes included in the model

Polymer Lifecycle Optimization Program (PolyLOP) was constructed to cover the production of each plastic type and all its precursors through both fossil-based and alternative feedstock-based routes, including CO_2-based and lignocellulose residue-based ones. Non-drop-in plastics may replace a specified fraction of conventional functional equivalents in certain applications, with these maximum replacement percentages defined based on technical substitution potentials that consider material properties and applications. We aimed to cover as comprehensive a set of processes as possible, focusing especially on those with high technology-readiness levels as indicated by previous research.[21,22] Several end-of-life treatment strategies were integrated into PolyLOP, including incineration, mechanical recycling, and plastic waste gasification (an example of chemical recycling). In addition, a process for capturing and storing process CO_2 in the plastic industry was incorporated. This process functions as a negative emission technology when the CO_2 is of biogenic origin.

Figure S3 presents an overview of the major plastic production routes and processes in the process based on fossil fuels and alternative feedstock. This subsection provides comprehensive lists of processes included in the model and their corresponding data sources for gate-to-gate inventories. In general, there are three types of data sources: lifecycle inventory databases (ecoinvent 3.10, allocation cut-off by classification system model and agri-footprint 6), the Process Economics Program (PEP) Yearbook from IHSMarkit[23],

and other literature. Lifecycle inventory databases and PEP require additional licenses, but the working procedures and key assumptions are documented in Section S1.4.5 and Section S1.4.6. The lifecycle inventories derived from other sources are provided in S20, with key assumptions documented in Section S1.4.7.

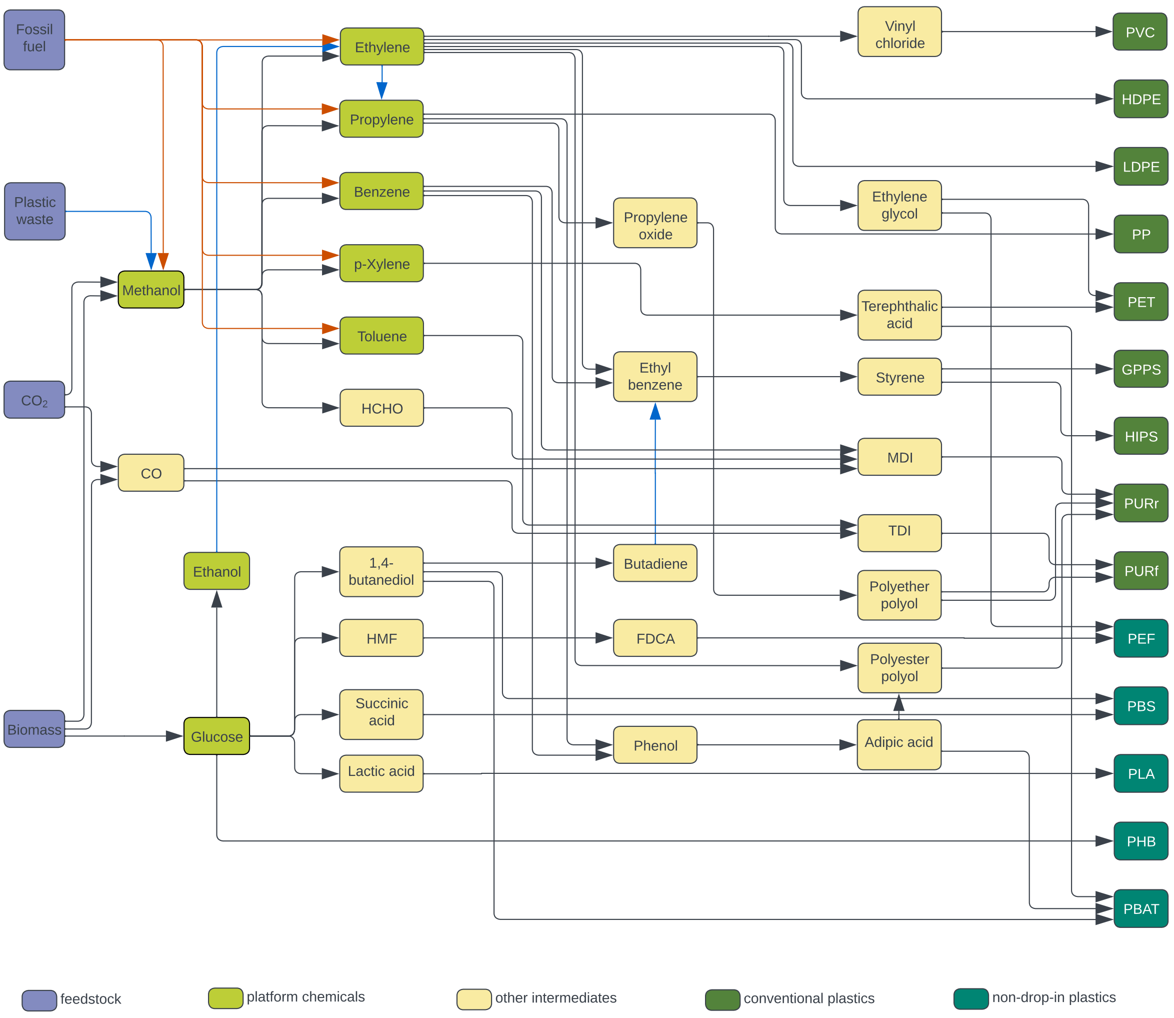

Figure S3: Major plastic production processes included in the model. Abbreviations: CO_2, carbon dioxide; CO, carbon monoxide; HCHO, Formaldehyde; HMF, 5-hydroxymethylfurfural; FDCA, 2,5-furandicarboxylic acid; MDI, methylenediphenyl diisocyanate; TDI, toluene diisocyanate. For the full names for plastics, see Table S1.

S1.3.1 Fossil-based plastics production

Table S5: Fossil-based plastics production processes

Process name	Product name	Data source
acetic_acid, from methanol carbonylation	acetic_acid	IHSMarkit
adipic_acid, from phenol via cyclohexanol	adipic_acid	IHSMarkit
ammonia, anhydrous, liquid, from ammonia production, steam reforming, liquid	ammonia	ecoinvent 3.10
aniline, from nitrobenzene	aniline	IHSMarkit
benzene, from BTX production, from pyrolysis gas, average	benzene	ecoinvent 3.10
benzene, from BTX production, from reformate, average	benzene	ecoinvent 3.10
butadiene, from butadiene purification, extractive distillation of crude butadiene	butadiene	ecoinvent 3.10
butadiene, crude, from unsaturated hydrocarbons production, steam cracking operation, average	butadiene_crude	ecoinvent 3.10
butane, from natural gas liquids fractionation	butane	ecoinvent 3.10
butene_1, from ethylene by dimerization	butene_1	IHSMarkit
butene, mixed, from unsaturated hydrocarbons production, steam cracking operation, average	butene_1	ecoinvent 3.10
carbon monoxide, from carbon monoxide production	carbon_monoxide	ecoinvent 3.10
cumene, from benzene and propylene	cumene	IHSMarkit
diesel, from diesel production, petroleum refinery operation	diesel	ecoinvent 3.10
diethylene_glycol, from ethylene via eo	diethylene_glycol	IHSMarkit
dinitrotoluene, from toluene by nitration	dinitrotoluene	IHSMarkit
ethane, from natural gas liquids fractionation	ethane	ecoinvent 3.10
ethylbenzene, from benzene via liquid phase alkylation	ethylbenzene	IHSMarkit
ethylene, from unsaturated hydrocarbons production, steam cracking operation, average	ethylene	ecoinvent 3.10
ethylene_glycol, from ethylene via eo	ethylene_glycol	IHSMarkit
ethylene_oxide, from ethylene by oxygen oxidation	ethylene_oxide	IHSMarkit
formaldehyde, from methanol	formaldehyde	IHSMarkit
gpps, by continuous bulk polymerization	gpps	IHSMarkit
hdpe, by gas phase process	hdpe	IHSMarkit
heavy fuel oil, from heavy fuel oil production, petroleum refinery operation	heavy_fuel_oil	ecoinvent 3.10
hips, by continuous bulk polymerization	hips	IHSMarkit
hydrogen, gaseous, low pressure, from hydrogen production, steam methane reforming	hydrogen	ecoinvent 3.10
ldpe, by gas phase process	ldpe	IHSMarkit
methanol, from methanol production, natural gas reforming	methanol	ecoinvent 3.10
naphtha, from naphtha production, petroleum refinery operation	naphtha	ecoinvent 3.10

Continued on next page

Table S5 continued

Process name	Product name	Data source
natural gas liquids, from natural gas liquids production	natural_gas_liquids	ecoinvent 3.10
nitric_acid, from ammonia	nitric_acid	Ecoinvent 3.10
nitrobenzene, from benzene by nitration	nitrobenzene	IHSMarkit
nitrogen, from crogenic air separation, improved	nitrogen_liquid	literature[24]
oxygen, from cryogenic air separation, improved	oxygen_liquid	literature[24]
pet, by tpa process	pet	IHSMarkit
phenol, from cumene	phenol	IHSMarkit
polybutadiene, by solution process	polybutadiene	IHSMarkit
polymeric_mdi, by phosgenation	polymeric_mdi	IHSMarkit
polyol_polyester, from adipic acid and deg	polyol_polyester	IHSMarkit
polyol_polyether, from po and eo by semi-continuous process	polyol_polyether	IHSMarkit
pp, by gas phase process	pp	IHSMarkit
propane, from natural gas liquids fractionation	propane	ecoinvent 3.10
propylene, from ethylene by dimerization and oct	propylene	IHSMarkit
propylene, from potroleum refinery openration	propylene	ecoinvent 3.10
propylene, from unsaturated hydrocarbons production, steam cracking operation, average	propylene	ecoinvent 3.10
propylene oxide, by the hppo process	propylene_oxide	IHSMarkit
pur flexible, from toluene diisocyanate and polyol	pur_flexible	IHSMarkit
pur rigid, from polymeric MDI and polyol	pur_rigid	IHSMarkit
pvc, by suspension polymerization	pvc	IHSMarkit
p-xylene, from BTX production, from pyrolysis gas, average	p-xylene	ecoinvent 3.10
p-xylene, from BTX production, from reformate, average	p-xylene	ecoinvent 3.10
pyrolysis gas, containing benzene, toluene and xylene, from unsaturated hydrocarbons production, steam cracking operation, average	pyrolysis_gas	ecoinvent 3.10
reformate, from reformate production, petroleum refinery operation	reformate	ecoinvent 3.10
styrene, from ethylbenzene by dehydrogenation	styrene	IHSMarkit
sulfur dioxide, from sulfur	sulfur_dioxide	ecoinvent
terephthalic acid, from p-xylene by air oxidation	terephthalic_acid	IHSMarkit
toluene, liquid, from BTX production, from pyrolysis gas, average	toluene	ecoinvent 3.10
toluene, liquid, from BTX production, from reformate, average	toluene	ecoinvent 3.10
toluene diisocyanate, from dinitrotoluene by phosgenation	toluene_diisocyanate	IHSMarkit
vinyl chloride, from ethylene by a balanced process	vinyl_chloride	IHSMarkit

S1.3.2 Alternative feedstock-based plastics production

We aimed to cover as comprehensive a set of processes as possible, focusing especially on those with high technology-readiness levels as indicated by previous research.[22,25]

Alternative feedstock-based drop-in plastics production The supply chain for drop-in plastics mirrors that of conventional plastics to a large extent, with methanol serving as an intermediate. Methanol can be produced through co_2 hydrogenation or via gasification of lignocellulose residues into syngas, followed by catalytic conversion of this syngas to methanol. The hydrogenation of co_2 needs hydrogen, and so its production from defossilized pathways, specifically through water electrolysis powered by renewable electricity, was also included in the process inventory.

Table S6: Alternative feedstock-based drop-in plastics production processes

Process name	Product name	Data source
ammonia, from Haber-Bosch process	ammonia	literature[25]
ammonium sulfate, from ammonia and sulfuric acid	ammonium_sulfate	ecoinvent 3.10
benzene, from MTA	benzene	literature[25]
carbon monoxide, from co2	carbon_monoxide	literature[25]
carbon monoxide, from partial condensation of syngas	carbon_monoxide	IHSMarkit
corn steep liquor, from corn	corn_steep_liquor	agri-footprint 6
enzyme, from potato starch	enzyme	ecoinvent 3.10
ethanol, from liquid hot water pre-treatment of lignocellulose biomass, followed by glucose fermentation and carbon capture	ethanol	literature[26]
ethylene, from ethanol dehydration	ethylene	IHSMarkit
ethylene, from methanol-to-olefins	ethylene	literature[27]
glucose, from organosolv pre-treatment of lignocellulose biomass	glucose	literature[28]
hydrogen, from PEM electrolysis	hydrogen	literature[29]
methanol, from lignocellulose residue gasification, with carbon capture	methanol	literature[30–33]
methanol, from co2 hydrogenation	methanol	literature[34–36]
potato starch, from potato	potato_starch	ecoinvent 3.10
propylene, from methanol-to-olefins	propylene	literature[27]
p-xylene, from MTA	p-xylene	literature[25]
syngas, from lignocellulose biomass gasification	syngas	literature[25]
toluene, from MTA	toluene	literature[25]

Non-drop-in plastics production

Table S7: Alternative feedstock-based non-drop-in plastics production processes

Process name	Product name	Data source
1,4-BDO, from glucose fermentation	bdo_14	literature[37]
cellulose, from organosolv pre-treatment of lignocellulose residues	cellulose	literature[28]
fdca, from cellulose via HMF	fdca	literature[38]
lactic acid, from steam explosion pre-treatment of lignocellulose residues	lactic_acid	literature[39]
pbat, from aliphatic aromatic copolyester process	pbat	IHSMarkit
pbs, from 1,4-BDO and succinic acid	pbs	literature[37]
pef, from fdca	pef	IHSMarkit
phb, from glucose fermentation	phb	literature[40]
pla, by ring-opening polymerization	pla	IHSMarkit
succinic acid, from glucose	succinic_acid	literature[37]
tetrahydrofuran, from 1,4-BDO dehydrogenation	tetrahydrofuran	ecoinvent 3.10
trimethylamine, from methanol and ammonia, mass balance only	trimethylamine	mass balance[iii]

Table S8: Potential replacement of conventional plastics with non-drop-in alternatives and their substitution factors by application within subsectors

Subsector	Non-drop-in plastics	Conventional plastics	Data source	Substitution factor[iv]
Beverage bottles	PLA	HDPE	literature[41]	0.80
Beverage bottles	PEF	PET	literature[42]	0.80
Beverage bottles	PLA	PET	literature[37,42–47v]	0.92
Consumer non-food bags	PLA	HDPE	literature[48]	1.77
Consumer non-food bags	PLA	LDPE	density proxy[vi]	1.35
Consumer non-food bags	PLA	PET	density proxy[vi]	0.91
Containers for food	PLA	GPPS	literature[6,49,50]	1.30
Containers for food	PBS	GPPS	density proxy[vi]	1.19
Containers for food	PLA	PET	literature[6,49,51]	0.98
Containers for food	PLA	PP	density proxy[vi]	1.37
Containers for non-food	PLA	GPPS	density proxy[vi]	1.16
Containers for non-food	PLA	PET	density proxy[vi]	0.91
Other food packaging	PLA	GPPS	literature[52–55]	1.18
Other food packaging	PLA	HDPE	density proxy[vi]	1.30

Continued on next page

[iii] Due to a lack of data, only the raw materials based on their stoichiometric relationships to the product are included.

[iv] A substitution factor describes how much non-drop-in plastics (kg) is needed to replace 1 kg of conventional plastics for the application in the given subsector.

[v] In case of multiple literature sources are found, the average across sources are used.

[vi] The density of the conventional plastics divided by the density of the non-drop-in plastics.

Table S8 continued

Subsector	Non-drop-in plastics	Conventional plastics	Data source	Substitution factor[iv]
Other food packaging	PLA	PET	literature[55–57]	0.86
Other food packaging	PLA	PP	literature[55–58]	1.14
Other food packaging	PHB	PP	density proxy[vi]	1.38
Other food packaging	PHB	GPPS	density proxy[vi]	1.17
Food films	PLA	LDPE	literature[59]	1.36
Food films	PLA	PET	density proxy[vi]	0.91
Food films	PLA	PP	literature[55]	1.00
Medical and hygiene items	PHB	HDPE	density proxy[vi]	1.31
Medical and hygiene items	PHB	PP	density proxy[vi]	1.38
Medical and hygiene items	PLA	HDPE	density proxy[vi]	1.30
Medical and hygiene items	PLA	LDPE	literature[60]	0.93
Medical and hygiene items	PLA	PP	literature[60]	0.93
Mulch films	PBAT	LDPE	literature[61,62]	0.85
Mulch films	PBS	LDPE	literature[63]	1.00
Mulch films	PLA	LDPE	literature[64]	1.00
Non-food films	PLA	PP	literature[54]	1.17

S1.3.3 Plastic waste treatment

PolyLOP includes four plastic waste treatment processes: incineration, mechanical recycling, gasification, and pyrolysis. Landfills were excluded in alignment with policies such as the European prohibition on landfill use from 2030.[65] Additionally, landfills may release harmful monomers, additives and other chemicals present in the plastic products, as well as micro- and nanoplastics, through leachates, posing risks to humans and wildlife.[66,67] Such pollution effects could not be accounted for in this study due to many knowledge and data gaps, and future work is needed to comprehensively evaluate the impacts of landfilling plastic waste.

Mechanical recycling was modeled for HDPE, LDPE, PET, PP, GPPS, HIPS, and PVC. Previous studies demonstrate a drastic drop in mechanical properties when recycling biodegradable plastics such as PLA,[68] and therefore, non-drop-in plastics were assumed to be unsuitable for mechanical recycling. We defined the "true recycling rate" as the ratio of applicable plastic waste content in recycled materials to total waste collected[14] and assumed that mechanically recycled products could replace virgin plastics with a substitution factor of 1 (e.g., 1 kg of recycled HDPE can replace 1 kg of virgin HDPE).

Chemical recycling via gasification produces methanol, modeled for HDPE, LDPE, PP, GPPS and HIPS following Prifti et al.[69] The gasification process involved plastic gasification in an entrained flow reactor at 850 °C with oxygen, steam methane reforming of light hydrocarbons, syngas conditioning with CO_2 capture, methanol synthesis, and methanol distillation to 99.85% purity.

Chemical recycling via pyrolysis was modeled for HDPE, LDPE, PP, GPPS and HIPS

based on the PEP Yearbook.[23] The process converted plastic waste into liquid hydrocarbon feedstock with 64% yield.The other gas and solid byproducts were assumed to be used as fuel for steam production. The liquid hydrocarbon feedstock were assumed to be used in steam crackers, replacing the existing fossil-based feedstock in the original steam cracking process from ecoinvent database (i.e., 65% naphtha, 16% butane, with other minor feedstock including propane, ethane, diesel, and natural gas liquids).

For non-drop-in biodegradable plastics, incineration was assumed as the primary end-of-life treatment. Recycling of biodegradable plastics is challenging due to a lack of separate collection systems and rapid degradation of their mechanical properties.[70] While these materials can theoretically biodegrade, they typically require specialized composting facilities and degrade very slowly and incompletely in natural environments.[71] Moreover, composting may emit CH_4 alongside CO_2, especially when not properly managed. CH_4 has a global warming potential 28 times higher than CO_2. Unless biodegradable plastics are properly collected and treated in industrial anaerobic digestion facilities with biogas recovery and utilization, biodegradation results in worse climate impacts than incineration due to direct CH_4 emissions to the atmosphere. Due to these limitations and the difficulty of proper disposal, incineration represents the most practical disposal route for biodegradable plastics, except for specific applications where material collection is challenging (e.g., some agricultural films).

All remaining plastic waste was assumed to be incinerated without energy recovery. Under a net-zero 2050 scenario, the electricity grid is expected to achieve very low carbon intensity, making energy recovery from incineration offer negligible carbon benefits compared to grid electricity substitution. CO_2 emissions from incineration were calculated stoichiometrically, assuming complete oxidation of all carbon content in the plastic waste to CO_2.

Table S9: Plastic waste treatment processes

Process name	Product name	Data source
hdpe, from mechanical recycling	hdpe	literature[14]
ldpe, from mechanical recycling	ldpe	literature[14]
pet, from mechanical recycling	pet	literature[14]
pp, from mechanical recycling	pp	literature[14]
gpps, from mechanical recycling	gpps	literature[14]
pvc, from mechanical recycling	pvc	literature[14]
hips, from mechanical recycling	hips	literature[14]
methanol, from pp waste gasification	methanol	literature[69]
methanol, from ldpe waste gasification	methanol	literature[69]
methanol, from hdpe waste gasification	methanol	literature[69]
methanol, from gpps waste gasification	methanol	literature[69]
methanol, from hips waste gasification	methanol	literature[69]
liquid hydrocarbon, from pp waste pyrolysis	liquid_hydrocarbon	IHSMarkit
liquid hydrocarbon, from ldpe waste pyrolysis	liquid_hydrocarbon	IHSMarkit
liquid hydrocarbon, from hdpe waste pyrolysis	liquid_hydrocarbon	IHSMarkit
liquid hydrocarbon, from gpps waste pyrolysis	liquid_hydrocarbon	IHSMarkit

Continued on next page

Table S9 continued

Process name	Product name	Data source
liquid hydrocarbon, from hips waste pyrolysis	liquid_hydrocarbon	IHSMarkit
incineration from hdpe waste	–	ecoinvent 3.10
incineration from ldpe waste	–	ecoinvent 3.10
incineration from pp waste	–	ecoinvent 3.10
incineration from gpps waste	–	ecoinvent 3.10
incineration from pvc waste	–	ecoinvent 3.10
incineration from pur flexible waste	–	ecoinvent 3.10
incineration from pur rigid waste	–	ecoinvent 3.10
incineration from pet waste	–	ecoinvent 3.10
incineration from hips waste	–	ecoinvent 3.10
incineration from pbat waste	–	ecoinvent 3.10
incineration from pbs waste	–	ecoinvent 3.10
incineration from pla waste	–	ecoinvent 3.10
incineration from phb waste	–	ecoinvent 3.10
incineration from pef waste	–	ecoinvent 3.10

S1.3.4 Storage of captured CO_2

Table S10: CO_2 storage processes

Process name	Product name	Data source
CCS	–	literature[72]

S1.3.5 Utilities

Table S11: Utility processes

Process name	Product name	Data source
cooling water	cooling_water	literature[33]
electricity, from lignocellulose residues	electricity_biogenic	literature[19,73]
heat, district or industrial, natural gas, from heat production, natural gas, at industrial furnace low-NOx >100 kW	heat	ecoinvent 3.10
heat, from lignocellulose residues	heat	literature[73,74]
steam, high temperature, from heat	steam	ecoinvent 3.10

S1.4 Life cycle inventory data

S1.4.1 Background data

In life cycle assessment (LCA), background data refer to the pre-existing environmental impact information for raw materials and energy sources that are not modeled gate-to-gate (i.e., foreground system, see Section S1.4.5 to Section S1.4.7) within this study. For example, the foreground system of this study does not include the detailed gate-to-gate modeling of natural gas from its extraction. Instead, it incorporates the integrated cradle-to-gate impacts of natural gas from the ecoinvent 3.10 database.

The study uses background data for four categories of raw materials: carbon feedstock, inorganic materials, other bio-based materials, and utilities. For our prospective LCA, the premise tool (v1.4.1)[72] is used to transform the ecoinvent 3.8 database into prospective life cycle inventories under the SSP2-RCP1.9 scenario of the IMAGE model, as the latest version (ecoinvent 3.10) is not yet compatible with premise. However, ecoinvent 3.10 is used for the oil and gas sector data. Compared to ecoinvent 3.8, this newer version incorporates crucial updates, including expanded geographical coverage and inclusion of unintended methane emissions during extraction and processing.[75] As the oil and gas sector is upstream in the supply chain, it is less affected by the decarbonization of background datasets that premise addresses, making this slight inconsistency in data source acceptable for our analysis.

Table S12: System raw materials

Raw material	Unit	Type	Impacts source
natural_gas	kg	carbon feedstock	ecoinvent 3.10
petroleum	kg	carbon feedstock	ecoinvent 3.10
co2_feedstock	kg	carbon feedstock	cut-off, zero impacts[vii]
barley_straw	kg	carbon feedstock	Huo et al.[20], regionalized data
forest_residue	kg	carbon feedstock	Huo et al.[20], regionalized data
maize_stover	kg	carbon feedstock	Huo et al.[20], regionalized data
rapeseed_straw	kg	carbon feedstock	Huo et al.[20], regionalized data
rice_straw	kg	carbon feedstock	Huo et al.[20], regionalized data
sorghum_straw	kg	carbon feedstock	Huo et al.[20], regionalized data
soybean_straw	kg	carbon feedstock	Huo et al.[20], regionalized data
sugarcane_tops _and_leaves	kg	carbon feedstock	Huo et al.[20], regionalized data
wheat_straw	kg	carbon feedstock	Huo et al.[20], regionalized data
chlorine	kg	inorganic material	Premise modified ecoinvent 3.8, global average
hydrochloric_acid	kg	inorganic material	Premise modified ecoinvent 3.8, global average
lime	kg	inorganic material	Premise modified ecoinvent 3.8, global average
magnesium_oxide	kg	inorganic material	Premise modified ecoinvent 3.8, global average
magnesium_sulfate	kg	inorganic material	Premise modified ecoinvent 3.8, global average
phosphate_rock	kg	inorganic material	Premise modified ecoinvent 3.8, global average
potassium_sulfate	kg	inorganic material	Premise modified ecoinvent 3.8, global average
sodium_hydroxide	kg	inorganic material	Premise modified ecoinvent 3.8, global average
sodium_hypochlorite	kg	inorganic material	Premise modified ecoinvent 3.8, global average
sodium_phosphate	kg	inorganic material	Premise modified ecoinvent 3.8, global average
sulfur	kg	inorganic material	Premise modified ecoinvent 3.8, global average
sulfuric_acid	kg	inorganic material	Premise modified ecoinvent 3.8, global average
maize_grain	kg	other bio-based material	Huo et al.[20], regionalized data
potato	kg	other bio-based material	Modeled with the methods descripted in Huo et al.[20], regionalized data
glycerin	kg	other bio-based material	Premise modified ecoinvent 3.8, global average
sds	kg	other bio-based material	Premise modified ecoinvent 3.8, global average
yeast	kg	other bio-based material	Premise modified ecoinvent 3.8, global average
electricity_non _biomass[viii]	kWh	utility	Premise modified ecoinvent 3.8, regionalized

[vii] CO_2-feedstock captured from other industries is considered burden-free for the plastics industry, as it is counted as a waste treatment measure for the source industries. As a result, these source industries get the credits of avoiding CO_2 emissions, but also bear the cost and impact of additional energy needed to perform carbon capture.[19]

[viii] The electricity grid excluding biomass-fired electricity production is included here. Biomass-fired electricity is modeled separately in the model (see Table S10).

S1.4.2 Allocation

For multi-output processes, allocation needs to be performed to distribute the raw material and utility consumption between the main product and the by-products. In the baseline case, economic allocation is performed when the price information is available (e.g., for IHSMarkit-derived processes). If the price information is not available, mass allocation is used instead (e.g., a lignocellulose biorefinery that coproduce ethanol and xylose[26] or the one that coproduce glucose and organosolv lignin[28]).

S1.4.3 Particulate matter-related emissions from biomass combustion and plastic waste incineration

The emission factors for the future incineration of plastics waste and biomass are based on the current, comparably strict emission limits of the European Union. For plastics waste incineration, concentration-based emission limits of NO_x at 200 $mgNm^{-3}$ flue gas, SO_2 at 50 $mgNm^{-3}$ and PM at 10 $mgNm^{-3}$ (all at 11 % oxygen content in the flue gas with of 273.15 K, a pressure of 101.3 kPa and corrected for water content) were used.[76] These emission limits were then converted to emission factors per amount of fuel input assuming that the flue gas is behaving like an ideal gas, plastics waste consists of 75 % carbon by weight, it has a lower heating value of 35 MJ kg^{-1}, the carbon is completely oxidized to CO_2, and air consists of 21 % oxygen by volume. Influences to the combustion from other constituents of the plastics waste other than carbon were neglected.

In an analogous way, biomass concentration-based emission limits of NO_x at 150 $mgNm^{-3}$ flue gas, SO_2 at 150 $mgNm^{-3}$ and PM at 20 $mgNm^{-3}$ (all at 6 % oxygen content in the flue gas) were selected from the European Union legislation[76], and converted to emission factors by assuming a 45 % carbon content by weight, and a lower heating value of 15 MJ/kg. All other assumptions in the case of biomass combustion were the same as for plastics waste incineration. Due to the low emission intensities, it was furthermore for both cases assumed that all PM was emitted in the form of PM2.5, which is a conservative, but good approximation for modern flue gas treatment.

S1.4.4 N_2O emissions from adipic acid production

The guidelines from the Intergovernmental Panel on Climate Change (IPCC)[77] for a tier 1 emissions factor of nitrous oxide emissions from adipic acid production were used with the assumption of the highest possible abatement efficiency.

S1.4.5 Gate-to-gate inventories based on the Process Economics Program (PEP) Yearbook from IHSMarkit

Each PEP data sheet contains information regarding inventory flows including the main product, raw material consumption, utilities consumption (including steam, heat, electricity and process water), and by-products, if applicable. It also contains all relevant price information. For each PEP-derived process in Table S5, Table S6 and Table S7, if there is

more than one data sheet available, the average value of all data sheets for each inventory flow is calculated.

To transform PEP data sheets into life-cycle inventories, the following simplifications and assumptions are made:

- A cut-off of 0.025 kg kg^{-1} main product is set. Raw materials and by-products below the cut-off value are assumed to have very limited impacts and are hence excluded from the life-cycle inventory .
- Gases reported in a volume unit are updated into a mass unit (kg) based on the density (Table S13).
- Fuels reported in a mass unit are updated into an energy unit (MJ) based on the lower calorific values (LHV) (Table S14). If system expansion is used, when fuels are co-produced with a main product, it is assumed that heat is produced from the fuel co-product with an efficiency of 90 %. In addition, emissions from fuel combustion are considered based on the established European and international guidelines (Table S15 and Table S16).
- For steam, a heat content of 3.2 MJ kg^{-1} is assumed based on PEP.
- Process emissions are added according to Table S17.
- For raw materials with a purity less than 100 % (typically in solution), only the non-water mass is considered. When the purity is not specified in percentage (e.g. "nitric acid, dilute" is used as raw material), it is estimated based on the price (e.g. the price of "nitric acid, dilute" / the price of pure nitric acid is assumed to be the purity of "nitric acid, dilute").
- When butadiene is used as raw material, sometimes mixed C4 flows from the steam cracker is reported as raw material, and butadiene raffinate is reported as co-products. As butadiene is assumed to be produced from ethanol in the alternative-feedstock pathway, only pure butadiene is assumed to be used in the model (consumption amount = mixed C4 flows − butadiene raffinate).

Table S13: Gas density under normal temperature and pressure

Gas	Density (kg m^{-3})[ix][78]
Air	1.205
Inert gas[x]	1.165
Hydrogen	0.08375

Continued on next page

[ix]Gas density under normal temperature and pressure (defined as 20°C and 1 atm)
[x]Assume nitrogen

Table S13 continued

Gas	Density ($kg\,m^{-3}$)[ix][78]
Hydrogen (99 vol%)	0.09496[xi]
Nitrogen	1.165
Carbon monoxide	1.165
Synthesis gas (2:1)	0.4434[xi]

Table S14: Lower Calorific Values for fuels

Fuel name as in IHSMarkit	LHV ($MJ\,kg^{-1}$)[79]
Fuel, liquid (credit)	39.8[xii]
Methane	50.0
Light and heavy ends	39.8[xii]
Fuel oil, low sulfur	39.8[xii]
Light ends credit	40.6[xiii]

Table S15: GHG emissions of fuel combustion

Fuel name as in IHSMarkit	GHG emission ($kg\,CO_2$-eq MJ^{-1})[80]
All liquid or unspecified fuel (fuel; fuel, heavy liquids; fuel, liquid; fuel oil; fuel oil, low sulfur; fuel oil, residue; light ends credit)	0.0733
All gas fuel (fuel gas; natural gas)	0.0561

Table S16: PM-related emissions of fuel combustion

Fuel name as in IHSMarkit	Pollutant	Amount (kg pollutant MJ^{-1})[73]
All liquid or unspecified fuel (fuel; fuel, heavy liquids; fuel, liquid; fuel oil; fuel oil, low sulfur; fuel oil, residue; light ends credit)	NO_X	6.50E-05
	PM2.5	8.00E-07
	SO_2	4.65E-05
All gas fuel (fuel gas; natural gas)	NO_X	8.90E-05
	PM2.5	1.40E-07
	SO_2	2.44E-07

[xi] Calculated based on volume ratio: hydrogen (99 vol%): 99% hydrogen + 1% air; synthesis gas (2:1): 66.7% hydrogen + 33.3% carbon monoxide

[xii] Average of heavy fuel oil and light fuel oil

[xiii] Assume light fuel oil

Table S17: Additional emissions added to PEP-derived processes

Product	Process	Pollutant	Amount ($kg\,kg^{-1}$ product)
Adipic acid	From phenol via cyclohexanol	N_2O	0.01191[77][xiv]
Ethylene oxide	From ethylene by oxygen oxidation	CO_2	0.251[xv]
PVC	By suspension polymerization	PM2.5	5.00E-06

S1.4.6 Gate-to-gate inventories based on life-cycle inventories

Each ecoinvent/agrifootprint-derived process listed in Table S5, Table S6, Table S7 and Table S9 is matched with its corresponding activity and product name from ecoinvent 3.10 and agrifootprint 6 in Table S18. When multiple locations are available for a single activity, we prioritize European activity locations in the following order: RER (Europe), Europe without Switzerland, RER (Europe) / RER w/o RU (Europe without Russia), and DE (Germany). This choice is based on ecoinvent's more comprehensive data coverage for Europe compared to other regions. For many processes, data for non-European regions may be limited or absent, and in some cases, data for other regions are extrapolated from European data.

We employ the "allocation cut-off by classification" system model, which pre-allocates all inventories to co-products based on their economic values when an activity yields multiple products.[81] To simplify the processes in the model, we apply the following criteria:

- Technosphere inputs (raw materials and utilities): We set a cut-off value of $0.025\,kg\,kg^{-1}$ of main product. Inputs below this threshold are excluded from the life cycle assessment, assuming their impacts to be negligible.
- Emissions: We focus exclusively on GHG and PM related emissions.

Table S18: ecoinvent-derived processes

Model process name	Model product name	ecoinvent product	ecoinvent activity	ecoinvent location
ammonia, anhydrous, liquid, from ammonia production, steam reforming, liquid	ammonia	ammonia, anhydrous, liquid	ammonia production, steam reforming, liquid	RER w/o RU

Continued on next page

[xiv] Based on Tier 1 emission factors in the IPCC guideline, with thermal destruction abatement (98.5% N_2O destruction factor and 97% N_2O abatement technology utilization factor

[xv] Calculated based on the carbon loss from carbon in ethylene as raw material and carbon in ethylene oxide as product

Table S18 continued

Model process name	Model product name	ecoinvent product	ecoinvent activity	ecoinvent location
ammonium sulfate, from ammonia and sulfuric acid	ammonium _sulfate	ammonium sulfate	ammonium sulfate production	RER
benzene, from BTX production, from pyrolysis gas, average	benzene	benzene	BTX production, from reformate, average	RER
benzene, from BTX production, from reformate, average	benzene	benzene	BTX production, from pyrolysis gas, average	RER
butadiene, crude, from unsaturated hydrocarbons production, steam cracking operation, average	butadiene _crude	butadiene, crude	unsaturated hydrocarbons production, steam cracking operation, average	RER
butadiene, from butadiene purification, extractive distillation of crude butadiene	butadiene	butadiene	butadiene purification, extractive distillation of crude butadiene	RER
butane, from natural gas liquids fractionation	butane	butane	natural gas liquids fractionation	GLO
butene, mixed, from unsaturated hydrocarbons production, steam cracking operation, average	butene_1	butene, mixed	unsaturated hydrocarbons production, steam cracking operation, average	RER
carbon monoxide, from carbon monoxide production	carbon _monoxide	carbon monoxide	carbon monoxide production	RER
diesel, from diesel production, petroleum refinery operation	diesel	diesel	diesel production, petroleum refinery operation	Europe without Switzerland
enzyme, from potato starch	enzyme	enzymes	enzymes production	RER
ethane, from natural gas liquids fractionation	ethane	ethane	natural gas liquids fractionation	GLO
ethylene, from unsaturated hydrocarbons production, steam cracking operation, average	ethylene	ethylene	unsaturated hydrocarbons production, steam cracking operation, average	RER

Continued on next page

Table S18 continued

Model process name	Model product name	ecoinvent product	ecoinvent activity	ecoinvent location
heat, district or industrial, natural gas, from heat production, natural gas, at industrial furnace low-NOx >100 kW	heat_high	heat, district or industrial, natural gas	heat production, natural gas, at industrial furnace low-NOx >100 kW	Europe without Switzerland
heavy fuel oil, from heavy fuel oil production, petroleum refinery operation	heavy_fuel_oil	heavy fuel oil	heavy fuel oil production, petroleum refinery operation	Europe without Switzerland
hydrogen, gaseous, low pressure, from hydrogen production, steam methane reforming	hydrogen	hydrogen, gaseous, low pressure	hydrogen production, steam methane reforming	RER
isobutane, from natural gas liquids fractionation	isobutane	isobutane	natural gas liquids fractionation	GLO
methanol, from methanol production, natural gas reforming	methanol	methanol	methanol production, natural gas reforming	RER
naphtha, from naphtha production, petroleum refinery operation	naphtha	naphtha	naphtha production, petroleum refinery operation	Europe without Switzerland
natural gas liquids, from natural gas liquids production	natural_gas _liquids	natural gas liquids	natural gas liquids production	GLO
nitric_acid, from ammonia	nitric_acid	nitric acid, without water, in 50% solution state	nitric acid production, product in 50% solution state	RER w/o RU
pentane, from natural gas liquids fractionation	pentane	pentane	natural gas liquids fractionation	GLO
potato starch, from potato	potato_starch	potato starch	potato starch production	DE
propane, from natural gas liquids fractionation	propane	propane	natural gas liquids fractionation	GLO
propylene, from potroleum refinery openration	propylene	C3 hydrocarbon mixture	C3 hydrocarbon production, mixture, petroleum refinery operation	Europe without Switzerland

Continued on next page

Table S18 continued

Model process name	Model product name	ecoinvent product	ecoinvent activity	ecoinvent location
propylene, from unsaturated hydrocarbons production, steam cracking operation, average	propylene	propylene	unsaturated hydrocarbons production, steam cracking operation, average	RER
p-xylene, from BTX production, from pyrolysis gas, average	p-xylene	p-xylene	BTX production, from pyrolysis gas, average	RER
p-xylene, from BTX production, from reformate, average	p-xylene	p-xylene	BTX production, from reformate, average	RER
pyrolysis gas, containing benzene, toluene and xylene, from unsaturated hydrocarbons production, steam cracking operation, average	pyrolysis_gas	pyrolysis gas, containing benzene, toluene and xylene	unsaturated hydrocarbons production, steam cracking operation, average	RER
reformate, from reformate production, petroleum refinery operation	reformate	reformate	reformate production, petroleum refinery operation	Europe without Switzerland
steam, high temperature, from heat	steam	heat, from steam, in chemical industry	steam production, as energy carrier, in chemical industry	RER
sulfur dioxide, from sulfur	sulfur _dioxide	sulfur dioxide, liquid	sulfur dioxide production, liquid	RER
tetrahydrofuran, from 1,4-BDO dehydrogenation	tetrahydrofuran	tetrahydrofuran	tetrahydrofuran production	RER
toluene, liquid, from BTX production, from pyrolysis gas, average	toluene	toluene, liquid	BTX production, from pyrolysis gas, average	RER
toluene, liquid, from BTX production, from reformate, average	toluene	toluene, liquid	BTX production, from reformate, average	RER
incineration from plastic waste	–	waste plastic, mixture	treatment of waste plastic, mixture, municipal incineration	GLO

Table S19: Agrifootprint-derived processes

Model process name	Model product name	Agrifootprint product	Agrifootprint activity	Agrifootprint location
corn steep liquor, from corn[xvi]	corn_steep _liquor	maize steepwater wet	maize steepwater wet, at processing	RER

S1.4.7 Gate-to-gate inventories based on other sources

Table S20 lists the gate-to-gate inventories for processes derived from the literature listed in Table S5–S11. The following assumptions are taken:

- If agricultural residue / forest residue is used as raw material, CO_2 emissions are calculated based on the mass balance of carbon, assuming agricultural residues have a carbon content of 49.4 % and forest residues have a carbon content of 52.1 % based on Vassilev et al.[82]
- **1,4-BDO, from glucose fermentation:** during fermentation, 0.98 kg CO_2 is co-produced with 1 kg of butanediol by stoichiometry. This high-purity CO_2 is assumed to be captured with 0.17 kWh electricity per kg CO_2 for pumping and compression. The rest CO_2 emissions are calculated based on the mass balance of carbon.
- **ethanol, from liquid hot water pre-treatment of agricultural residue / forest residue, followed by glucose fermentation and carbon capture:** During the fermentation of glucose, 0.96 kg CO_2 is co-produced with 1 kg of ethanol by stoichiometry. This high-purity CO_2 is assumed to be captured with 0.17 kWh electricity kg^{-1} CO_2 for pumping and compression.
- **methanol, from lignocellulose residue gasification, with carbon capture:** The life cycle inventory of methanol production from biomass gasification is taken from Charalambous et al.[33] However, the authors assume a rather high biomass utilization rate of 1.7 kg biomass / kg methanol. This is adjusted to 1.99 kg, as the average (1.7–2.3 kg) across literature we have reviewed.[30–33] CO_2 emissions are re-calculated accordingly based on carbon mass balance.
- **methanol, from CO_2 hydrogenation:** The average raw material consumptions and emissions from the literature are considered.
- **electricity, from agricultural residue / forest residue:** electricity is modeled with the efficiency of best available technology of 38 %.[19] The average heating values for agricultural residues and forest residues are assumed to be 17.5 and 20 MJ kg^{-1} dry mass (DM), respectively.[83]

[xvi]Corn steep liquor is co-produced during the wet milling of corn and is used as a minor input for ethanol production from biomass fermentation.

- **heat, high temperature, from agricultural residue / forest residue:** 90 % thermal efficiency is assumed. The average heating values for agricultural residues and forest residues are assumed to be 17.5 and 20 MJ kg^{-1} DM, respectively.[83]

Table S20: Gate-to-gate life-cycle inventories of processes derived from other sources

Process	Flow names	Flow type	Unit	Value
1,4-BDO, from glucose fermentation	bdo_14	product	kg	1.00
	co2_emission	emission	kg	1.23
	co2_feedstock	by-product credits	kg	0.88
	electricity	utilities	kWh	-0.30
	glucose	raw materials	kg	-2.77
	phosphate_rock	raw materials	kg	-0.08
	sodium_hydroxide	raw materials	kg	-0.06
	steam	utilities	MJ	-1.73
	water	raw materials	kg	-2.93
	yeast	raw materials	kg	-0.02
ammonia, from Haber-Bosch process	ammonia	product	kg	1.00
	electricity	utilities	kWh	-0.60
	hydrogen	raw materials	kg	-0.18
	nh3_emission	emission	kg	1E-5
	nitrogen_liquid	raw materials	kg	-0.84
	nox_emission	emission	kg	1E-3
benzene, from MTA	benzene	product	kg	1.00
	cooling_water	utilities	MJ	-0.49
	electricity	utilities	kWh	-0.12
	methanol	raw materials	kg	-2.59
	steam	utilities	MJ	-4.94
butadiene, from ethanol to butadiene process	butadiene	product	kg	1.00
	co2_emission	emission	kg	0.31
	electricity	utilities	kWh	-0.80
	ethanol	raw materials	kg	-1.88
	heat	utilities	MJ	-9.56
	hydrogen_peroxide	raw materials	kg	-0.04
	steam	utilities	MJ	-21.92
	water	raw materials	kg	-18.36
carbon_monoxide, from co2	carbon_monoxide	product	kg	1.00
	co2_feedstock	raw materials	kg	-1.65
	electricity	utilities	kWh	-0.28
	heat	utilities	MJ	-2.00
	hydrogen	raw materials	kg	-0.08
	water	utilities	kg	-0.64

Continued on next page

Table S20 continued

Process	Flow name	Flow type	Unit	Value
carbon_monoxide, from partial condensation of syngas	carbon_monoxide	product	kg	1.00
	cooling_water	utilities	MJ	-0.65
	electricity	utilities	kWh	-0.32
	syngas_2_to_1	raw materials	kg	-0.67
CCS	co2_emission	emission	kg	0.00
	co2_feedstock	raw materials	kg	-1.00
	co2_storage	product	kg	1.00
	electricity	utilities	kWh	-0.01
	pipeline	raw materials	tkm	0.00
cellulose, from organosolv pre-treatment of agricultural residue	agricultural_residue	raw materials	kg	-1.21
	cellulose	product	kg	1.00
	co2_emission	emission	kg	0.90
	cooling_water	utilities	MJ	-0.51
	electricity	utilities	kWh	-0.29
	enzyme	raw materials	kg	0.00
	heat	utilities	MJ	9.40
	steam	utilities	MJ	-2.10
	water	raw materials	kg	-7.63
cellulose, from organosolv pre-treatment of forest residue	cellulose	product	kg	1.00
	co2_emission	emission	kg	1.02
	cooling_water	utilities	MJ	-0.51
	electricity	utilities	kWh	-0.29
	enzyme	raw materials	kg	0.00
	forest_residue	raw materials	kg	-1.21
	heat	utilities	MJ	9.40
	steam	utilities	MJ	-2.10
	water	raw materials	kg	-7.63
cooling water	cooling_water	product	MJ	1.00
	electricity	utilities	kWh	0.00
	water	raw materials	kg	-0.62
electricity_biogenic, from agricultural residue	agricultural_residue	raw materials	kg	-0.54
	co2_emission	emission	kg	0.98
	electricity	product	kWh	1.00
	nox_emission	emission	kg	5.40E-4
	pm25_emission	emission	kg	7.58E-5
	sox_emission	emission	kg	5.40E-4
	water	raw materials	kg	-0.01

Continued on next page

Table S20 continued

Process	Flow name	Flow type	Unit	Value
electricity_biogenic, from forest residue	co2_emission	emission	kg	0.90
	electricity	product	kWh	1.00
	forest_residue	raw materials	kg	-0.47
	nox_emission	emission	kg	5.40E-4
	pm25_emission	emission	kg	7.58E-5
	sox_emission	emission	kg	5.40E-4
	water	raw materials	kg	-0.01
ethanol, from liquid hot water pre-treatment of agricultural residue	agricultural_residue	raw materials	kg	-2.92
	ammonium_sulfate	raw materials	kg	-0.10
	co2_emission	emission	kg	2.85
	co2_feedstock	by-product credits	kg	0.84
	corn_steep_liquor	raw materials	kg	-0.10
	ethanol	product	kg	1.00
	glucose	raw materials	kg	-0.24
	sulfuric_acid	raw materials	kg	-0.10
	water	raw materials	kg	-14.43
ethanol, from liquid hot water pre-treatment of forest residue	ammonium_sulfate	raw materials	kg	-0.10
	co2_emission	emission	kg	3.17
	co2_feedstock	by-product credits	kg	0.84
	corn_steep_liquor	raw materials	kg	-0.10
	ethanol	product	kg	1.00
	forest_residue	raw materials	kg	-2.92
	glucose	raw materials	kg	-0.24
	sulfuric_acid	raw materials	kg	-0.10
	water	raw materials	kg	-14.43
ethylene, from methanol-to-olefins	electricity	utilities	kWh	-0.46
	ethylene	product	kg	1.00
	methanol	raw materials	kg	-2.57
	steam	utilities	MJ	-5.59
fdca, from cellulose via HMF	cellulose	raw materials	kg	-1.85
	cooling_water	utilities	MJ	-2.34
	electricity	utilities	kg	-0.12
	fdca	product	kg	1.00
	heat	utilities	MJ	-1.32
	lime	raw materials	kg	-0.04
	oxygen_liquid	raw materials	kg	-0.88
	sulfuric_acid	raw materials	kg	-0.13
	tetrahydrofuran	raw materials	kg	-0.03
glucose, from organosolv pre-treatment of agricultural residue	agricultural_residue	raw materials	kg	-1.44
	co2_emission	emission	kg	1.07

Continued on next page

Table S20 continued

Process	Flow name	Flow type	Unit	Value
	cooling_water	utilities	MJ	-0.61
	electricity	utilities	kWh	-0.34
	enzyme	raw materials	kg	0.00
	glucose	product	kg	1.00
	steam	utilities	MJ	-2.50
	water	raw materials	kg	-9.11
glucose, from organosolv pre-treatment of forest residue	co2_emission	emission	kg	1.21
	cooling_water	utilities	MJ	-0.61
	electricity	utilities	kWh	-0.34
	enzyme	raw materials	kg	0.00
	forest_residue	raw materials	kg	-1.44
	glucose	product	kg	1.00
	steam	utilities	MJ	-2.50
	water	raw materials	kg	-9.11
gpps_mr, from mechanical recycling	electricity	utilities	kWh	-0.83
	gpps_mr	product	kg	1.00
	gpps_waste	raw materials	kg	-1.00
	heat	utilities	MJ	-0.20
hdpe_mr, from mechanical recycling	electricity	utilities	kWh	-0.97
	hdpe_mr	product	kg	1.00
	hdpe_waste	raw materials	kg	-1.00
	heat	utilities	MJ	-0.28
heat, high temperature, from agricultural residue	agricultural_residue	raw materials	kg	-0.06
	co2_emission	emission	kg	0.12
	heat	product	MJ	1.00
	nox_emission	emission	kg	6.33E-5
	pm25_emission	emission	kg	8.89E-6
	sox_emission	emission	kg	6.33E-5
heat, high temperature, from forest residue	co2_emission	emission	kg	0.11
	forest_residue	raw materials	kg	-0.06
	heat	product	MJ	1.00
	nox_emission	emission	kg	6.33E-5
	pm25_emission	emission	kg	8.89E-6
	sox_emission	emission	kg	6.33E-5
hips_mr, from mechanical recycling	electricity	utilities	kWh	-1.00
	heat	utilities	MJ	-0.20
	hips_mr	product	kg	1.00
	hips_waste	raw materials	kg	-1.00
hydrogen, from PEM electrolysis	electricity	utilities	kWh	-54.00

Continued on next page

Table S20 continued

Process	Flow name	Flow type	Unit	Value
	hydrogen	product	kg	1.00
	water	raw materials	kg	-14.00
lactic acid, from steam explosion pre-treatment of agricultural residue	agricultural_residue	raw materials	kg	-2.01
	co2_emission	emission	kg	2.19
	cooling_water	utilities	MJ	-2.36
	corn_steep_liquor	raw materials	kg	-0.05
	lactic_acid	product	kg	1.00
	sodium_hydroxide	raw materials	kg	0.00
	sulfur_dioxide	raw materials	kg	-0.02
	trimethylamine	raw materials	kg	-0.02
	water	raw materials	kg	-16.24
lactic acid, from steam explosion pre-treatment of forest residue	co2_emission	emission	kg	2.39
	cooling_water	utilities	MJ	-2.36
	corn_steep_liquor	raw materials	kg	-0.05
	forest_residue	raw materials	kg	-2.01
	lactic_acid	product	kg	1.00
	sodium_hydroxide	raw materials	kg	0.00
	sulfur_dioxide	raw materials	kg	-0.02
	trimethylamine	raw materials	kg	-0.02
	water	raw materials	kg	-16.24
ldpe_mr, from mechanical recycling	electricity	utilities	kWh	-0.93
	heat	utilities	MJ	-0.26
	ldpe_mr	product	kg	1.00
	ldpe_waste	raw materials	kg	-1.00
methanol, from agricultural residue gasification, with co2 capture	agricultural_residue	raw materials	kg	-1.99
	co2_emission	emission	kg	1.00
	co2_feedstock	by-product credits	kg	1.36
	cooling_water	utilities	MJ	-4.35
	electricity	utilities	kWh	-0.15
	methanol	product	kg	1.00
methanol, from co2 hydrogenation	co2_emission	emission	kg	0.08
	co2_feedstock	raw materials	kg	-1.46
	electricity	utilities	kWh	-0.26
	hydrogen	raw materials	kg	-0.19
	methanol	product	kg	1.00
methanol, from forest residue gasification, with co2 capture	co2_emission	emission	kg	1.20
	co2_feedstock	by-product credits	kg	1.36
	cooling_water	utilities	MJ	-4.35
	electricity	utilities	kWh	-0.15
	forest_residue	raw materials	kg	-1.99

Continued on next page

Table S20 continued

Process	Flow name	Flow type	Unit	Value
	methanol	product	kg	1.00
methanol, from gpps_waste gasification	co2_emission	emission	kg	0.68
	co2_feedstock	by-product credits	kg	0.80
	electricity	utilities	kWh	-0.44
	gpps_waste	raw materials	kg	-0.81
	heat	utilities	MJ	-4.40
	methanol	product	kg	1.00
	oxygen_liquid	raw materials	kg	-0.64
	water	raw materials	kg	-0.80
methanol, from hdpe_waste gasification	co2_emission	emission	kg	0.68
	co2_feedstock	by-product credits	kg	0.80
	electricity	utilities	kWh	-0.44
	hdpe_waste	raw materials	kg	-0.81
	heat	utilities	MJ	-4.40
	methanol	product	kg	1.00
	oxygen_liquid	raw materials	kg	-0.64
	water	raw materials	kg	-0.80
methanol, from hips_waste gasification	co2_emission	emission	kg	0.68
	co2_feedstock	by-product credits	kg	0.80
	electricity	utilities	kWh	-0.44
	heat	utilities	MJ	-4.40
	hips_waste	raw materials	kg	-0.81
	methanol	product	kg	1.00
	oxygen_liquid	raw materials	kg	-0.64
	water	raw materials	kg	-0.80
methanol, from ldpe_waste gasification	co2_emission	emission	kg	0.68
	co2_feedstock	by-product credits	kg	0.80
	electricity	utilities	kWh	-0.44
	heat	utilities	MJ	-4.40
	ldpe_waste	raw materials	kg	-0.81
	methanol	product	kg	1.00
	oxygen_liquid	raw materials	kg	-0.64
	water	raw materials	kg	-0.80
methanol, from pp_waste gasification	co2_emission	emission	kg	0.68
	co2_feedstock	by-product credits	kg	0.80
	electricity	utilities	kWh	-0.44
	heat	utilities	MJ	-4.40
	methanol	product	kg	1.00
	oxygen_liquid	raw materials	kg	-0.64
	pp_waste	raw materials	kg	-0.81

Continued on next page

Table S20 continued

Process	Flow name	Flow type	Unit	Value
	water	raw materials	kg	-0.80
nitrogen, from crogenic air separation, improved	electricity	utilities	kWh	-0.26
	nitrogen_liquid	product	kg	1.00
oxygen, from cryogenic air separation, improved	electricity	utilities	kWh	-0.25
	oxygen_liquid	product	kg	1.00
pbs, from 1,4-BDO and succinic acid	bdo_14	raw materials	kg	-0.68
	cooling_water	utilities	MJ	-0.01
	electricity	utilities	kWh	-0.07
	pbs	product	kg	1.00
	steam	utilities	MJ	-8.25
	succinic_acid	raw materials	kg	-0.69
	water	raw materials	kg	-0.49
pet_mr, from mechanical recycling	electricity	utilities	kWh	-0.75
	heat	utilities	MJ	-0.16
	pet_mr	product	kg	1.00
	pet_waste	raw materials	kg	-1.00
phb, from glucose fermentation	ammonia	raw materials	kg	-0.05
	ammonium_sulfate	raw materials	kg	-0.01
	co2_emission	emission	kg	1.22
	co2_feedstock	by-product credits	kg	0.92
	cooling_water	utilities	MJ	-19.29
	electricity	utilities	kWh	-1.39
	glucose	raw materials	kg	-2.86
	magnesium_sulfate	raw materials	kg	-0.02
	phb	product	kg	1.00
	potassium_sulfate	raw materials	kg	-0.02
	sds	raw materials	kg	-0.29
	sodium_hypochlorite	raw materials	kg	-0.14
	steam	utilities	MJ	-10.89
	water	raw materials	kg	-2.63
pp_mr, from mechanical recycling	electricity	utilities	kWh	-0.96
	heat	utilities	MJ	-0.27
	pp_mr	product	kg	1.00
	pp_waste	raw materials	kg	-1.00
propylene, from methanol-to-olefins	electricity	utilities	kWh	-0.46
	methanol	raw materials	kg	-2.57
	propylene	product	kg	1.00
	steam	utilities	MJ	-5.59
pvc_mr, from mechanical recycling	electricity	utilities	kWh	-0.72

Continued on next page

Table S20 continued

Process	Flow name	Flow type	Unit	Value
	heat	utilities	MJ	-0.14
	pvc_mr	product	kg	1.00
	pvc_waste	raw materials	kg	-1.00
p-xylene, from MTA	cooling_water	utilities	MJ	-0.49
	electricity	utilities	kWh	-0.12
	methanol	raw materials	kg	-2.59
	p-xylene	product	kg	1.00
	steam	utilities	MJ	-4.94
steam, high temperature, from heat	electricity	utilities	kWh	-0.01
	heat	utilities	MJ	-1.28
	steam	product	MJ	1.00
	water	raw materials	kg	-0.06
succinic acid, from glucose	co2_feedstock	raw materials	kg	-0.26
	electricity	utilities	kWh	-1.76
	glucose	raw materials	kg	-1.28
	hydrochloric_acid	raw materials	kg	-0.40
	magnesium_oxide	raw materials	kg	-0.24
	sodium_hydroxide	raw materials	kg	-0.20
	succinic_acid	product	kg	1.00
	water	raw materials	kg	-3.49
	yeast	raw materials	kg	-0.09
syngas, from agricultural residue gasification	agricultural_residue	raw materials	kg	-1.56
	co2_emission	emission	kg	1.65
	electricity	utilities	kWh	-0.18
	oxygen_liquid	raw materials	kg	-0.72
	syngas_2_to_1	product	kg	1.00
	water	raw materials	kg	-1.06
syngas, from forest residue gasification	co2_emission	emission	kg	1.81
	electricity	utilities	kWh	-0.18
	forest_residue	raw materials	kg	-1.56
	oxygen_liquid	raw materials	kg	-0.72
	syngas_2_to_1	product	kg	1.00
	water	raw materials	kg	-1.06
toluene, from MTA	cooling_water	utilities	MJ	-0.49
	electricity	utilities	kWh	-0.12
	methanol	raw materials	kg	-2.59
	steam	utilities	MJ	-4.94
	toluene	product	kg	1.00

S1.5 Impact assessment

S1.5.1 Climate change impacts of biogenic CO_2 emissions

For biogenicCO_2 emissions not covered in the IPCC report, GWP100 values from Cherubini et al.[84] were used. In particular, for agricultural residues with a 1-year rotation period, GWP100bio is 0, while for forest residues, assuming a 90-year global average forest rotation period,[85] GWP100bio is 0.38.

S1.5.2 Land use–related biodiversity loss impacts

Lignocellulose residues and other bio-based feedstock Lignocellulose residues are used as carbon feedstock in PolyLOP. The full list can be found in Table S12. Their land use–related biodiversity loss impacts were quantified on the country level. A short methodology summary is provided here, while more details can be found in Huo et al.[20]

The estimates used data from the GLOBIOM model at 200 km × 200 km spatial resolution covering ten-year intervals from 2000 to 2050 under the SSP2 framework with the RCP1.9 climate scenario. This dataset included spatially explicit crop production volumes and fertilizer application rates for agricultural residues, stem wood and roundwood production volumes for calculating logging residue potential, process residues from sawmills, land use areas and change patterns, and regional prices for economic allocation. Both land occupation (ongoing impacts from current land use) and land transformation (impacts from converting between land types such as forest to cropland; i.e., land-use change) were considered, while accounting for management intensification effects such as increased harvesting intensity in managed forests for the 2050 scenarios. These data were coupled with the characterization factors (CFs) developed under the Global Life Cycle Impact Assessment Method (GLAM) Initiative applied at country-specific levels for regionalized assessments.[86] This method quantifies global species extinction risks from habitat destruction, degradation and fragmentation (measured as PDF, or potentially disappeared fraction of species).

The impact assessment employs economic allocation based on the regional prices from the GLOBIOM outputs,[20]) distributing impacts among all coproducts (including residues) according to their economic value—for example, allocating impacts between between main crops and agricultural residues. Land-use change impacts follow IPCC guidelines with a 20-year transitional period after land conversion, comparing GLOBIOM land use data between the assessment year and 20 years prior, with impacts distributed evenly over this period and allocated only to products from areas with increased harvest.

Petroleum and natural gas A regionalized assessment of land use–related biodiversity loss impact for petroleum and natural gas is not feasible, as the consolidation of all the exact locations for fossil fuel extraction are beyond the scope of this study. However, we conducted a simplified impact assessment using global average land use for petroleum and natural gas extraction, as well as the global average CFs of land use–related biodiversity loss impacts from Scherer et al.[86] Specifically, the land occupation data per unit petroleum and natural gas production were based on ecoinvent 3.10 (cut-off system model), for the

datasets "market for petroleum | RoW" and "market for natural gas, high pressure | RoW", respectively, where RoW stands for rest of the world. For CF from Scherer et al.[86], the study utilized the average approach for global relative species loss across the domain of Eukaryota, with original data quality and land use weighting for urban intense habitat. To account for spatial aggregation uncertainties in the global average CF, we conducted a sensitivity analysis. CF was varied between upper and lower bounds calculated as $\mathrm{CF} \times \exp\left(\pm \ln \sqrt{1+\mathrm{RSD}^2}\right)$, with the relative standard deviation (RSD) of global CF = 2.05.[86]. The log-normal distribution was considered for the CF uncertainty to consider only positive values.

S1.6 Linear optimization model: mathematical formulation

S1.6.1 Objective Function

PolyLOP can perform both single-objective and multi-objective optimization. The model also generates Pareto curves (see Fig. 1 in the Main Text for example) to explore trade-offs between impact categories by normalizing two impacts to a −1 to 1 scale and varying their relative weights in the optimization (i.e., multi-objective optimization).

This study primarily focuses on single-objective optimization minimizing climate change impacts, as achieving net-zero emissions is the main driver for the ongoing industry transition.

Single-objective optimization The mathematical formulation of the objective function is:

Minimize total environmental impacts [xvii]:

$$\min \sum_{p \in P} \Big(\sum_{r \in R_p} -\mathrm{Flow}_{p,r} \cdot \mathrm{Impact}_r + \sum_{b \in B_p} \mathrm{Flow}_{p,b} \cdot \mathrm{CF}_b \Big) \cdot X_p \tag{S1}$$

where

- P: Set of all processes
- R: Set of all raw materials added in the system (listed in Table S12
- B: Set of all biosphere flows
- $\mathrm{Flow}_{p,r}$: Technosphere flow for raw material $r \in R_p$ per unit of X_p, negative for input, positive for output/co-product (*parameters*)
- Impact_r: Cradle-to-gate impact for raw material $r \in R$ (*parameters*)
- $\mathrm{Flow}_{p,b}$: Biosphere flow $b \in B_p$ per unit of X_p, positive for emissions, negative for resource extractions (*parameters*)

[xvii] Environmental impacts can be climate change impacts, land use-related biodiversity loss, and/or PM-related health impacts

- CF_b: Characterization factor for biosphere flow $b \in B$ (*parameters*)
- X_p: Production unit for $p \in P$ (*decision variables*)

Multi-objective optimization When performing multi-objective optimization, impacts are the normalized weighted combinations of multiple impact categories. For example, to derive the Pareto curve in Fig. 1 in the Main Text, both climate change impacts (GHG) and land use–related biodiversity impacts (BDV) are normalized to a [-1,1] scale using their respective minimum and maximum values from single-objective optimizations:

$$\text{GHG}_{\text{norm}} = \frac{\text{GHG}_{\text{total}} - \text{GHG}_{\text{min}}}{\text{GHG}_{\text{max}} - \text{GHG}_{\text{min}}} \tag{S2}$$

$$\text{BDV}_{\text{norm}} = \frac{\text{BDV}_{\text{total}} - \text{BDV}_{\text{min}}}{\text{BDV}_{\text{max}} - \text{BDV}_{\text{min}}} \tag{S3}$$

where

- $\text{GHG}_{\text{total}}$ and $\text{BDV}_{\text{total}}$ are calculated as per Equation S1
- GHG_{min}: the minimum climate impact achieved when optimizing solely for climate objectives
- GHG_{max}: the climate impact when optimizing solely for biodiversity objectives
- BDV_{min}: 0, representing scenarios with no biomass use
- BDV_{max}: the land use–related biodiversity loss impact when all biomass is utilized

The Pareto frontier is generated by solving a series of optimization problems with varying weights:

$$\text{Minimize: } \omega \cdot \text{GHG}_{\text{norm}} + (1 - \omega) \cdot \text{BDV}_{\text{norm}} \tag{S4}$$

where ω varies from 0 to 1 in incremental steps. Each weight combination generates a point on the Pareto curve, representing different trade-offs between the two impact categories. When $\omega = 1$, the optimization prioritizes climate impacts exclusively, while $\omega = 0$ prioritizes biodiversity impacts exclusively.

S1.6.2 Constraints

1. **Exogenous plastics demand:**

$$\sum_{p \in P} \text{Flow}_{p,f} \cdot X_p = \text{demand}_f \quad \forall f \in F \tag{S5}$$

where

- F: Set of final products
- $\text{Flow}_{p,f}$: Technosphere flow $f \in F$ per unit of X_p, positive for output (*parameters*)
- demand_f: Exogenous demand for final product $f \in F$ (*parameters*)

2. **Supply limits for raw materials:**

$$\sum_{p \in P} -\text{Flow}_{p,r} \cdot X_p \leq \text{supply}_r \quad \forall r \in R \tag{S6}$$

where

- supply_r: Supply limit for raw material $r \in R$ (*parameters*)

3. **Mass balance for intermediate products:**

$$\sum_{p \in P} \text{Flow}_{p,i} \cdot X_p = 0 \quad \forall i \in I \tag{S7}$$

where

- I: Set of intermediate products
- $\text{Flow}_{p,i}$: Technosphere flow for intermediate $i \in I_p$ per unit of X_p, negative for input, positive for output (*parameters*)

4. **Waste treatment:**

$$\sum_{p \in P} \text{Flow}_{p,w} \cdot X_p = 0 \quad \forall w \in W \tag{S8}$$

where

- W: Set of plastic waste
- $\text{Flow}_{p,w}$: Technosphere flow for plastic waste $w \in W_p$ per unit of X_p, negative for input, positive for output (*parameters*)

5. **Ethylene and propylene ratio constraints from MTO process:** In the MTO process, the ratio of ethylene to propylene is adjustable within a defined range, specifically from 0.6 to 1.3.[87] This range sets the minimum and maximum limits for the relative quantities of ethylene and propylene produced from the process.

$$\begin{aligned} 0.6 \cdot X_{\text{MTO, propylene}} &\leq X_{\text{MTO, ethylene}} \\ X_{\text{MTO, propylene}} &\leq 1.3 \cdot X_{\text{MTO, ethylene}} \end{aligned} \tag{S9}$$

where

- $X_{\text{MTO, ethylene}}$: Production of ethylene from MTO process (*decision variable*)
- $X_{\text{MTO, propylene}}$: Production of propylene from MTO process (*decision variable*)

6. **Maximum mechanical recycling rate:**

For each mechanically recycled plastic type fmr:

$$\sum_{p \in P} \text{Flow}_{p,fmr} \cdot X_p \leq \text{RR}_{\text{mech},w} \cdot \sum_{p \in P} \text{Flow}_{p,w} \cdot X_p \quad \forall fmr \in F \tag{S10}$$

$$\sum_{p \in P} \text{Flow}_{p,fmr} \cdot X_p \leq \text{RC}_f \cdot \sum_{p \in P} \text{Flow}_{p,f} \cdot X_p \quad \forall fmr \in F \tag{S11}$$

where

- $\text{Flow}_{p,fmr}$: Technosphere flow for mechanically recycled plastic product $fmr \in F$ per unit of X_p, positive for output (*parameters*)
- $\text{RR}_{\text{mech},w}$: Maximum mechanical recycling rate for waste type $w \in W$ (*parameters*)
- RC_f: Maximum recycled content for final product $f \in F$ (*parameters*)

Table S21: Maximum end-of-life recycling rate and recycled content for mechanical recycling

Plastic type	End-of-life recycling rate	Recycled content
HDPE	0.44	0.42
LDPE	0.35	0.35
PET	0.45	0.45
PP	0.35	0.34
GPPS	0.24	0.22
PVC	0.31	0.27
HIPS	0.35	0.34

7. **Maximum chemical recycling rate:**

$$\sum_{p \in P_{\text{chem}}} -\text{Flow}_{p,w} \cdot X_p \leq \text{RR}_{\text{chem},w} \cdot \sum_{p \in P} \text{Flow}_{p,w} \cdot X_p \quad \forall w \in W \tag{S12}$$

where

- P_{chem} is the set of chemical recycling (gasification) processes (subset of P)
- $\text{RR}_{\text{chem},w}$: Maximum chemical recycling rate for waste type $w \in W$ (*parameters*)

Table S22: Maximum end-of-life recycling rate for chemical recycling

Plastic type	End-of-life recycling rate
HDPE	0.46

Continued on next page

Table S22 continued

Plastic type	End-of-life recycling rate
LDPE	0.57
PP	0.50
GPPS	0.64
HIPS	0.57

8. **Maximum substitution rate of non-drop-in plastics:** For each pair of non-drop-in plastic f and conventional plastic f':

$$\sum_{p \in P} \text{Flow}_{p,f} \cdot X_p \leq \text{sub\_rate}_{f,f'} \cdot \sum_{p \in P} \text{Flow}_{p,f'} \cdot X_p \tag{S13}$$

where

- $\text{sub\_rate}_{f,f'}$: Maximum substitution rate of non-drop-in plastic f for conventional plastic f' (*parameters*)

Table S23: Maximum substitution rate of non-drop-in plastics

Conventional plastics	Non-drop-in plastics				
	PHB	PLA	PBS[xviii]	PEF[xviii]	PBAT[xviii]
GPPS	0.2	0.1	0.2	0.2	0.2
HDPE	0.2	0.1	0.2	0.2	0.2
LDPE	0.2	0.1	0.2	0.2	0.2
PET	0.1	0.2	0.2	0.2	0.2
PP	0.1	0.1	0.2	0.2	0.2
PUR	0.05		0.2	0.2	0.2
PVC	0.1		0.2	0.2	0.2

9. **Non-negativity constraint for decision variables:**

$$X_p \geq 0 \quad \forall p \in P \tag{S14}$$

S1.7 Sensitivity analysis

S1.7.1 Biomass availability

In the net-zero scenario, the availability of lignocellulose residues was assumed to be 2.3 Gt[20] (=100 %). To account for potential competition for biomass from other sectors as well as higher estimates of availablity,[20] a biomass availability factor was introduced in the sensitivity analysis. This factor adjusted the maximum supply of lignocellulose residues available to the plastic industry. Assuming 2.3 Gt represented 100 % biomass availability, this constraint was varied between 0 % and 200 % in the sensitivity analysis. While Huo et al.[20] suggested a higher maximum potential availability of 5.2 Gt for lignocellulose residues, we set our upper bound at 200 % (4.6 Gt) as our test runs showed the system did not utilize biomass availability beyond this level, making it a practical limit for our analysis.

S1.7.2 Lock-in of fossil facilities

If no new capacity for fossil-based plastic production were to be added beyond current plans, a minimum of 265 MT fossil-based plastic production capacity would still be operational in 2050 (Figure S4). This projection is based on historical annual capacity additions and the assumption that fossil process equipment has an average lifespan of about 50 years. This sensitivity analysis excludes bio-based steam cracking feedstock (e.g., bio-naphtha, bio-ethane), which might be compatible with existing equipment due to the lack of reliable data regarding their future relevance and adoption rates in the industry. Specifically, the following methodology was followed:

[xviii] PBS, PEF and PBAT is not included by Spierling et al.[88], a maximum substitution rate of 0.2 is assumed for all conventional plastic types.

1. **Data Collection and Preparation:** Plastic production data from 1950 to 2019 was compiled and supplemented with actual plant-level data on capacity additions and facility shutdowns from 2020 to 2024.

2. **Historical Capacity Change Analysis:** Year-over-year changes in production capacity were calculated for each year from 1950 onwards. These historical changes serve as the basis for projecting future capacity reductions, assuming a 50-year facility lifespan.

3. **Future Capacity Projection:** Starting with the most recent known total capacity, we applied a 50-year turnover rule: for each future year, we subtracted the capacity increase observed 50 years prior. For instance, a capacity increase of 3.4 MT from 1980 to 1981 translates to a projected 3.4 MT capacity decrease from 2030 to 2031.

This methodology provides a simplified model of capacity turnover in the fossil-based plastics industry. It assumes a fixed 50-year operational lifespan for production facilities and does not account for potential technological advancements, policy changes, or market dynamics that could influence facility lifespans or capacity decisions. Regional variations in facility operations are also not considered in this model.

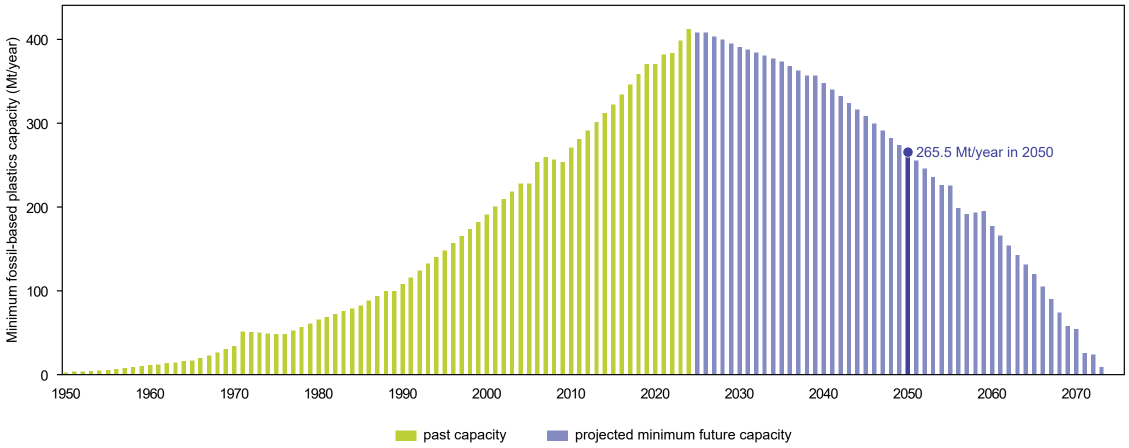

Figure S4: Minimum fossil-based plastic production capacity projection

S2 Supplementary results

S2.1 Supplementary results

S2.1.1 Carbon flow for the plastic industry

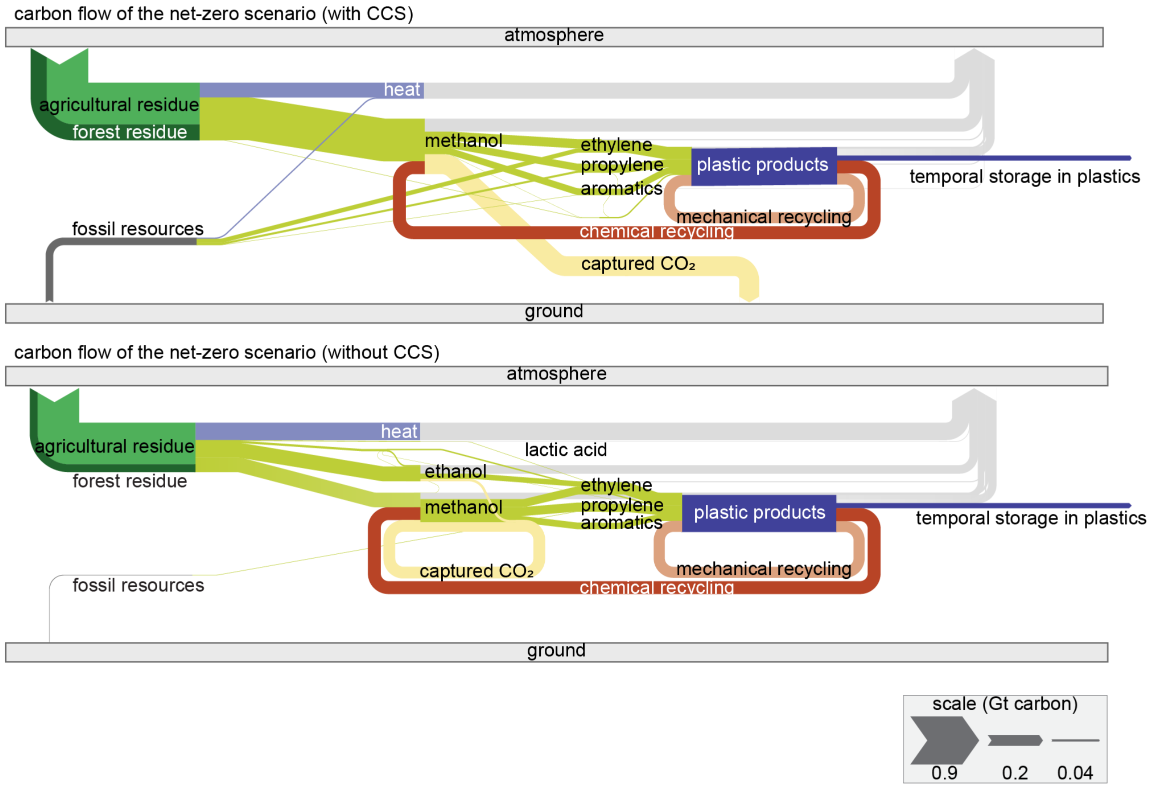

Figure S5: Carbon flow for the plastic industry in 2050 with minimum climate change impacts for the scenarios with and without CCS.

S2.1.2 Ammonia emissions from agricultural residues

Ammonia emissions from agricultural activities represent a primary driver of PM-related health impacts in the net-zero scenario, as these emissions contribute to secondary particulate matter formation in the atmosphere. Huo et al.[20] quantified ammonia emissions from nitrogen fertilizer application using IPCC tier 1 emission factors, with spatially explicit fertilizer application rates obtained from the GLOBIOM model. The life cycle inventory of agricultural activities were allocated between the main crops and their corresponding lignocellulose residues based on their market values obtained from GLOBIOM.

The results reveal substantial heterogeneity in ammonia emissions across crop residue types and geographic regions, ranging from 0.07 to 2.99×10^{-3} kg ammonia/kg residue dry mass (Figure S6). Sorghum straws and barley straws exhibit the highest ammonia emissions across most regions, reflecting the typically lower crop yields and correspondingly higher fertilizer intensity required per unit of biomass residue produced. Regional patterns indicate large geographic variability, with Western Africa generally showing lower ammonia emissions across all crop types due to limited fertilizer application, while intensive farming systems in China and the United States exhibit higher emissions, particularly for wheat straw.

These emission patterns have important implications for the environmental sustainability of lignocellulose residue utilization in net-zero plastic industry transitions. Effective mitigation of ammonia emissions requires implementation of precision fertilizer application technologies and improved nitrogen use efficiency through advanced crop breeding and management practices.

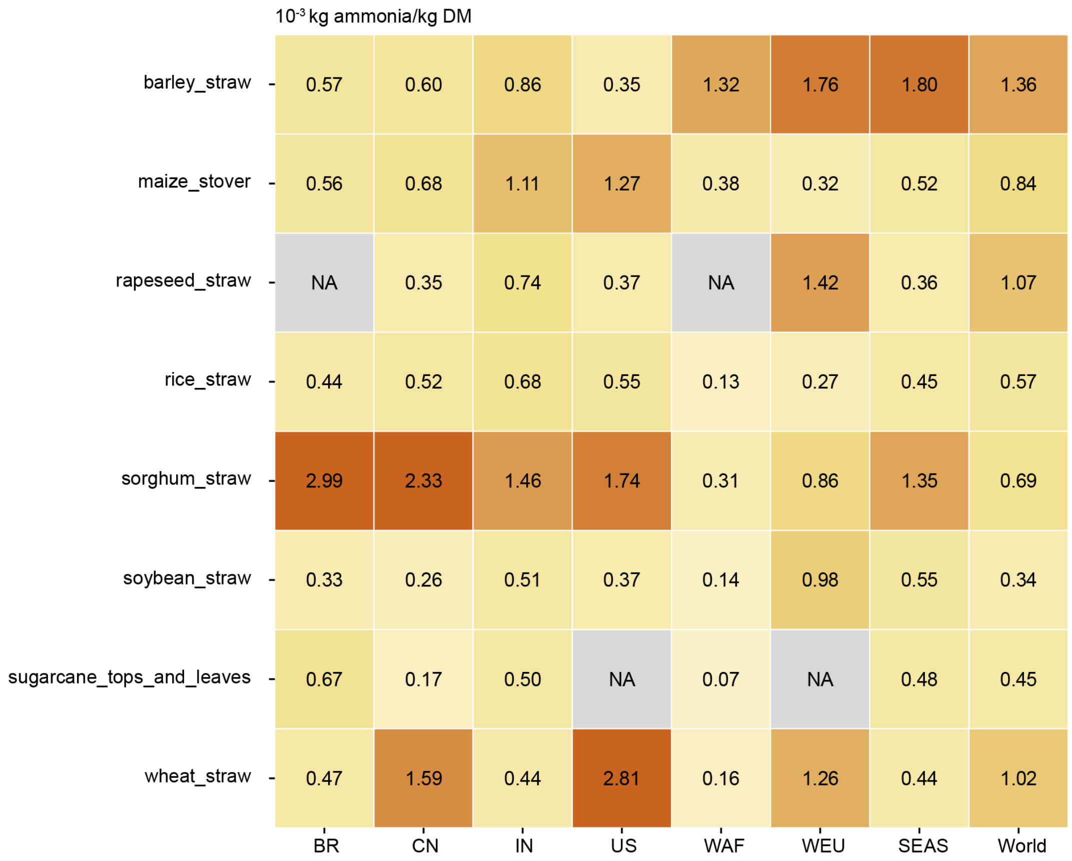

Figure S6: Cradle-to-gate ammonia emissions of lignocellulose residues under the RCP1.9 scenario in 2050. Abbreviations: DM, dry mass; BR, Brazil; CN, China; IN, India; US, the United States of America; WAF, Western Africa; WEU, Western Europe; SEAS, Southeast Asia. For region definitions, see Figure S1.

S2.1.3 Resource constraints

We conducted a sensitivity analysis by varying biomass availability from 0% to 200% and the carbon footprint of the electricity mix from 0 to 0.4 kg CO_2-eq/kWh. Without CCS, 1 Gt of CO_2 would be utilized to produce methanol when electricity has a zero carbon footprint. As a result, the electricity consumption would be 9 PWh (Figure S7b). This amount would decrease with increasing carbon footprint of the electricity mix, and when the electricity mix would have a carbon footprint above 0.12 kg CO_2-eq/kWh, it would become more climate-beneficial to re-emit the captured CO_2 from fermentation and gasification processes back to the atmosphere than its utilization as a feedstock. For scenarios with CCS, the captured CO_2 from fermentation and gasification would be preferred for storage. Only when electricity would have a carbon footprint below 0.03 kg CO_2-eq/kWh would CO_2-based production routes be chosen, utilizing CO_2 from point sources. Although the scope differs, Meys et al.[22] found the tipping point where CO_2-based routes are selected occurs with an electricity carbon footprint below 0.086 kg CO_2-eq/kWh. This threshold is higher than in our study because they considered CO_2 from direct air capture, which has a negative cradle-to-gate climate change impact, but is more energy-intensive and expensive, while we consider point sources that would still generate CO_2 even under the net-zero scenario in 2050.

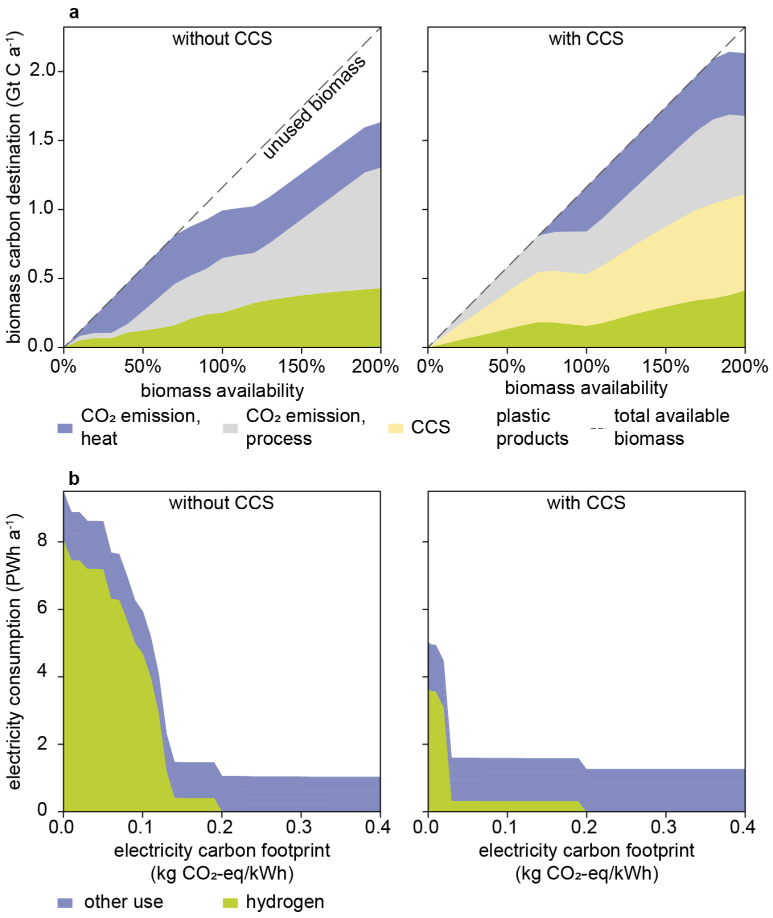

Figure S7: Impact of biomass availability and electricity carbon footprint on the climate change impacts of the optimized future plastic industry. a) destinations of the carbon in lignocellulose residues as a function of biomass availability for the scenarios without and with CCS. b) electricity consumption as a function of the electricity carbon footprint for the scenarios without and with CCS.

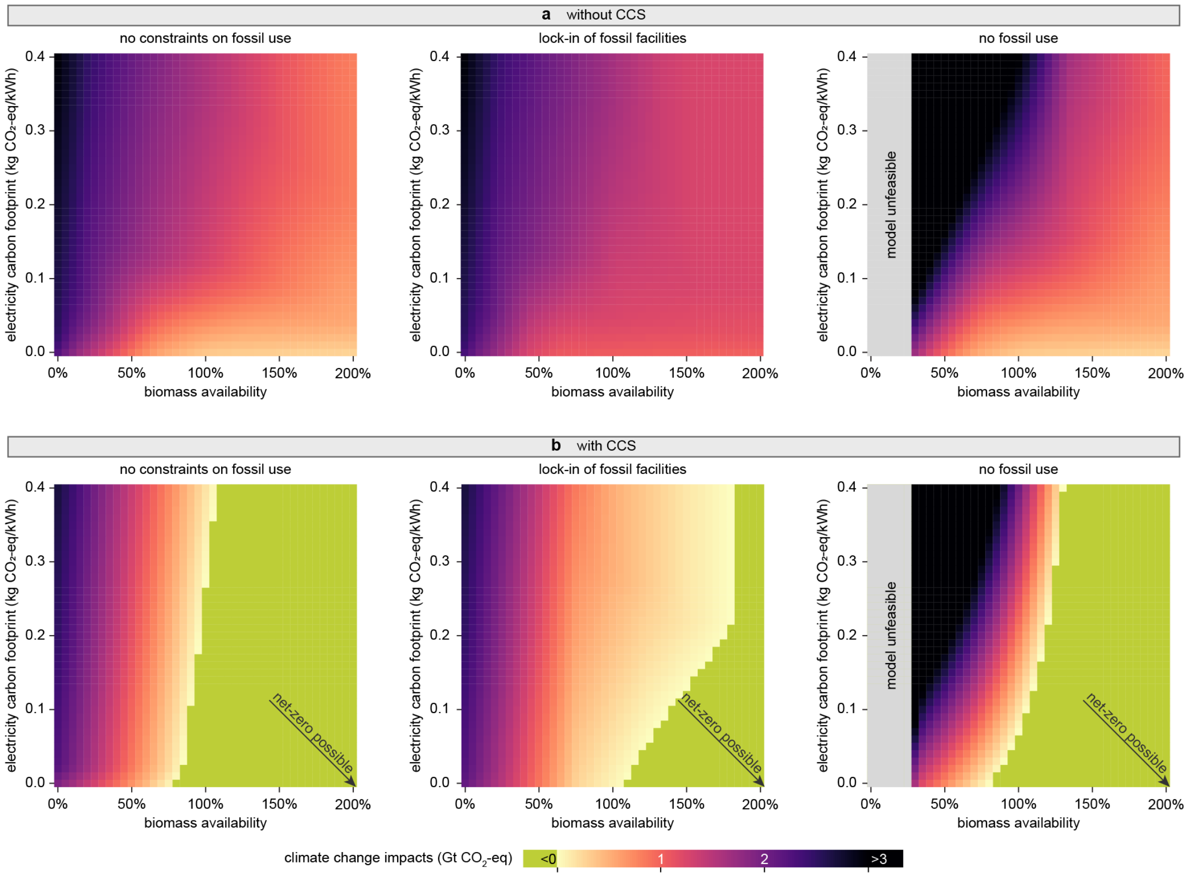

Figure S8: Minimum climate change impacts of the global plastic industry in 2050 as a function of electricity carbon footprint and biomass availability under different constraints regarding fossil use. a) scenarios with CCS; b) scenarios without CCS.

Table S24: Environmental impacts of regionally optimized plastic industry, by region and impact source. For region definitions, see Figure S2.

Country	Impact source	Climate change (Mt CO_2-eq)	Biodiversity loss ($\times 10^{-6}$ PDF)	Health (DALY)
CAN	Feedstock Fossil	6.33E-05	0.00	4.36E-03
	Feedstock Biomass	6.20	28.10	3575.78
	Feedstock Other	0.12	3.16E-05	25.14
	Electricity Grid	0.38	0.00	46.99
	Onsite Heat	1.32E-03	0.00	304.09
	Waste Treatment	1.65	0.00	229.06
	CCS	-20.49	0.00	0.00
	Onsite Process	4.22	0.00	20.15
CHN	Feedstock Fossil	71.88	0.00	23105.69
	Feedstock Biomass	20.98	147.59	91393.17
	Feedstock Other	3.24	0.00	2534.68
	Electricity Grid	20.19	0.00	8999.82
	Onsite Heat	135.86	0.00	2743.72
	Waste Treatment	91.45	0.00	17731.46
	CCS	-215.36	0.00	0.00
	Onsite Process	56.32	0.00	8387.45
KOR	Feedstock Fossil	7.75	0.00	2486.80
	Feedstock Biomass	0.23	22.78	556.82
	Feedstock Other	0.28	0.00	198.66
	Electricity Grid	0.80	0.00	566.25
	Onsite Heat	10.92	0.00	204.50
	Waste Treatment	16.14	0.00	1797.81
	CCS	-7.29	0.00	0.00
	Onsite Process	6.21	0.00	926.30
USA	Feedstock Fossil	6.39E-05	0.00	7.07E-03
	Feedstock Biomass	27.70	391.46	23857.27
	Feedstock Other	1.21	6.38E-04	333.15
	Electricity Grid	7.77	0.00	1353.68
	Onsite Heat	0.01	0.00	4468.57
	Waste Treatment	15.54	0.00	3324.68
	CCS	-195.99	0.00	0.00
	Onsite Process	40.26	0.00	262.75
JPN	Feedstock Fossil	4.15	0.00	2771.36
	Feedstock Biomass	0.45	33.14	972.94
	Feedstock Other	0.30	0.00	340.84
	Electricity Grid	2.01	0.00	4970.33
	Onsite Heat	14.49	0.00	558.95
	Waste Treatment	14.78	0.00	4537.04

Continued on next page

Table S24 continued

Country	Impact source	Climate change (Mt CO_2-eq)	Biodiversity loss ($\times10^{-6}$ PDF)	Health (DALY)
	CCS	-19.19	0.00	0.00
	Onsite Process	6.09	0.00	503.45
BRA	Feedstock Fossil	6.44E-05	0.00	1.16E-02
	Feedstock Biomass	11.58	300.04	8124.41
	Feedstock Other	0.35	1.84E-03	158.00
	Electricity Grid	2.27	0.00	187.58
	Onsite Heat	3.42E-03	0.00	2074.11
	Waste Treatment	5.06E-03	0.00	3133.24
	CCS	-64.71	0.00	0.00
	Onsite Process	6.61	0.00	148.98
IND	Feedstock Fossil	29.11	0.00	9869.43
	Feedstock Biomass	39.25	326.42	43312.89
	Feedstock Other	2.42	0.00	2196.85
	Electricity Grid	14.57	0.00	4937.30
	Onsite Heat	0.02	0.00	20189.83
	Waste Treatment	28.81	0.00	6756.23
	CCS	-164.69	0.00	0.00
	Onsite Process	46.04	0.00	6113.98
IDN	Feedstock Fossil	5.93	0.00	3150.26
	Feedstock Biomass	4.59	284.15	25145.93
	Feedstock Other	0.50	0.00	894.15
	Electricity Grid	3.04	0.00	1367.33
	Onsite Heat	3.59E-03	0.00	6505.65
	Waste Treatment	9.18	0.00	3512.80
	CCS	-33.16	0.00	0.00
	Onsite Process	11.76	0.00	3207.95
ZAF	Feedstock Fossil	0.11	0.00	9.13
	Feedstock Biomass	1.54	69.11	959.88
	Feedstock Other	0.10	3.41E-04	24.27
	Electricity Grid	0.42	0.00	58.87
	Onsite Heat	8.69E-04	0.00	248.09
	Waste Treatment	0.12	0.00	106.90
	CCS	-12.57	0.00	0.00
	Onsite Process	1.58	0.00	20.23
RUS	Feedstock Fossil	6.54E-05	0.00	7.25E-03
	Feedstock Biomass	5.53	23.52	2416.66
	Feedstock Other	0.31	2.32E-04	76.47
	Electricity Grid	6.58	0.00	888.83
	Onsite Heat	3.10E-03	0.00	1156.88

Continued on next page

Table S24 continued

Country	Impact source	Climate change (Mt CO_2-eq)	Biodiversity loss ($\times 10^{-6}$ PDF)	Health (DALY)
	Waste Treatment	8.96	0.00	1005.60
	CCS	-51.04	0.00	0.00
	Onsite Process	16.10	0.00	91.45
TUR	Feedstock Fossil	4.14	0.00	916.89
	Feedstock Biomass	3.82	57.91	5714.21
	Feedstock Other	0.20	0.00	110.53
	Electricity Grid	2.95	0.00	2757.65
	Onsite Heat	8.66	0.00	104.23
	Waste Treatment	3.83	0.00	708.34
	CCS	-15.92	0.00	0.00
	Onsite Process	3.29	0.00	271.28
MEX	Feedstock Fossil	4.67	0.00	992.23
	Feedstock Biomass	2.35	137.26	4439.85
	Feedstock Other	0.29	8.22E-05	173.95
	Electricity Grid	1.77	0.00	436.54
	Onsite Heat	10.72	0.00	394.12
	Waste Treatment	3.51	0.00	827.01
	CCS	-27.37	0.00	0.00
	Onsite Process	5.62	0.00	282.85
UKR	Feedstock Fossil	6.50E-05	0.00	1.04E-02
	Feedstock Biomass	3.65	28.97	3667.60
	Feedstock Other	0.08	7.83E-05	24.96
	Electricity Grid	0.98	0.00	408.16
	Onsite Heat	8.36E-04	0.00	452.86
	Waste Treatment	0.16	0.00	310.60
	CCS	-13.12	0.00	0.00
	Onsite Process	1.77	0.00	29.64
World	Feedstock Fossil	132.65	0.00	40310.42
	Feedstock Biomass	230.84	4233.66	415178.60
	Feedstock Other	15.65	2.90E-03	10244.83
	Electricity Grid	111.72	0.00	46739.80
	Onsite Heat	78.84	0.00	114394.58
	Waste Treatment	225.95	0.00	64647.14
	CCS	-1412.27	0.00	0.00
	Onsite Process	361.81	9.09E-13	21309.25
WEU	Feedstock Fossil	11.38	0.00	2957.83
	Feedstock Biomass	16.01	390.43	58723.70
	Feedstock Other	1.21	3.64E-04	698.17
	Electricity Grid	7.04	0.00	3273.56

Continued on next page

Table S24 continued

Country	Impact source	Climate change (Mt CO_2-eq)	Biodiversity loss ($\times 10^{-6}$ PDF)	Health (DALY)
	Onsite Heat	28.33	0.00	5144.63
	Waste Treatment	21.06	0.00	5828.02
	CCS	-133.29	0.00	0.00
	Onsite Process	35.74	5.68E-14	1388.06
OCE	Feedstock Fossil	6.26E-05	0.00	1.27E-03
	Feedstock Biomass	1.05	67.11	60.13
	Feedstock Other	0.11	5.22E-04	6.72
	Electricity Grid	0.19	0.00	8.61
	Onsite Heat	9.94E-04	0.00	68.58
	Waste Treatment	2.06	0.00	37.57
	CCS	-14.72	0.00	0.00
	Onsite Process	4.38	0.00	4.11
RSAF	Feedstock Fossil	0.34	0.00	36.48
	Feedstock Biomass	2.72	68.44	1629.40
	Feedstock Other	0.11	1.48E-03	35.33
	Electricity Grid	1.38	0.00	69.78
	Onsite Heat	8.72E-04	0.00	315.54
	Waste Treatment	0.08	0.00	132.85
	CCS	-12.45	0.00	0.00
	Onsite Process	2.12	1.42E-14	39.03
RSAM	Feedstock Fossil	6.47E-05	0.00	7.42E-03
	Feedstock Biomass	6.59	288.91	3107.29
	Feedstock Other	0.42	2.84E-03	130.18
	Electricity Grid	2.57	0.00	239.70
	Onsite Heat	4.47E-03	0.00	1737.63
	Waste Treatment	4.18	0.00	1169.66
	CCS	-70.31	0.00	0.00
	Onsite Process	14.86	0.00	91.40
WAF	Feedstock Fossil	7.12E-05	0.00	1.57E-02
	Feedstock Biomass	8.69	147.49	3802.23
	Feedstock Other	0.32	7.31E-03	202.43
	Electricity Grid	0.91	0.00	341.42
	Onsite Heat	2.95E-03	0.00	2190.42
	Waste Treatment	0.13	0.00	1790.87
	CCS	-54.22	0.00	0.00
	Onsite Process	8.07	0.00	144.21
EAF	Feedstock Fossil	22.37	0.00	4835.81
	Feedstock Biomass	2.86	82.36	2337.23
	Feedstock Other	0.86	0.00	464.83

Continued on next page

Table S24 continued

Country	Impact source	Climate change (Mt CO_2-eq)	Biodiversity loss ($\times 10^{-6}$ PDF)	Health (DALY)
	Electricity Grid	4.00	0.00	1149.31
	Onsite Heat	30.57	0.00	394.39
	Waste Treatment	19.76	0.00	1743.28
	CCS	-33.64	0.00	0.00
	Onsite Process	17.74	0.00	1815.27
CEU	Feedstock Fossil	6.38E-05	0.00	1.15E-02
	Feedstock Biomass	7.67	139.94	16179.70
	Feedstock Other	0.31	2.16E-04	125.35
	Electricity Grid	3.26	0.00	685.69
	Onsite Heat	3.40E-03	0.00	2074.97
	Waste Treatment	5.02	0.00	1630.18
	CCS	-52.85	0.00	0.00
	Onsite Process	12.22	0.00	163.31
RSAS	Feedstock Fossil	6.88E-05	0.00	4.31E-02
	Feedstock Biomass	16.14	95.61	30745.51
	Feedstock Other	0.30	4.93E-04	333.72
	Electricity Grid	1.20	0.00	936.77
	Onsite Heat	2.78E-03	0.00	5915.03
	Waste Treatment	0.04	0.00	4788.29
	CCS	-50.95	0.00	0.00
	Onsite Process	6.21	0.00	170.28
STAN	Feedstock Fossil	1.24	0.00	66.97
	Feedstock Biomass	3.11	16.53	1649.26
	Feedstock Other	0.12	0.00	21.43
	Electricity Grid	1.25	0.00	115.70
	Onsite Heat	8.90E-04	0.00	161.43
	Waste Treatment	1.82	0.00	89.23
	CCS	-8.85	0.00	0.00
	Onsite Process	2.37	0.00	59.93
RME	Feedstock Fossil	30.20	0.00	5167.27
	Feedstock Biomass	3.33	14.08	3697.07
	Feedstock Other	1.07	0.00	336.25
	Electricity Grid	7.50	0.00	1079.27
	Onsite Heat	38.88	0.00	167.28
	Waste Treatment	48.19	0.00	2203.96
	CCS	-25.39	0.00	0.00
	Onsite Process	21.66	0.00	1344.97
SEAS	Feedstock Fossil	7.16	0.00	2396.19
	Feedstock Biomass	26.28	567.34	25043.60

Continued on next page

Table S24 continued

Country	Impact source	Climate change (Mt CO_2-eq)	Biodiversity loss ($\times 10^{-6}$ PDF)	Health (DALY)
	Feedstock Other	0.88	0.00	721.41
	Electricity Grid	7.18	0.00	2192.41
	Onsite Heat	6.78E-03	0.00	7758.49
	Waste Treatment	6.61	0.00	4011.78
	CCS	-76.54	0.00	0.00
	Onsite Process	14.61	0.00	1796.71
RCAM	Feedstock Fossil	3.64	0.00	632.10
	Feedstock Biomass	2.05	109.46	3369.45
	Feedstock Other	0.20	0.00	94.25
	Electricity Grid	1.63	0.00	353.61
	Onsite Heat	7.99	0.00	130.33
	Waste Treatment	2.01	0.00	465.91
	CCS	-16.95	0.00	0.00
	Onsite Process	3.24	0.00	216.71
NAF	Feedstock Fossil	5.30	0.00	1495.01
	Feedstock Biomass	2.10	19.55	4208.36
	Feedstock Other	0.32	0.00	246.68
	Electricity Grid	2.74	0.00	499.71
	Onsite Heat	5.64	0.00	1226.84
	Waste Treatment	6.03	0.00	849.22
	CCS	-20.46	0.00	0.00
	Onsite Process	6.56	0.00	603.94

S2.2 Comparison with previous studies: climate change impacts of plastics

The plastics industry is responsible for 2 Gt CO_2-eq climate change impacts[89] for the production of 380 Mt of plastics in 2015.[13] If this emission profile is linearly extrapolated to the 1 Gt project plastics production in 2050, the business-as-usual (BAU) climate change impacts of the plastics industry would be 5.3 Gt. In Figure 1a in the Main Text, we conclude a fossil–linear plastics industry would cause at least 4.5 Gt CO_2-eq climate change impacts. Our model differs from the BAU projection in several key aspects:

1. Net-zero scenarios for background energy systems are employed (e.g., electricity carbon footprint) (see Section S1.2.1).

2. Coal-based production processes are excluded.

3. 100 % incineration of plastic waste is assumed, aligning with policies designed to reduce landfill usage[65], whereas current practices involve less than 20 % incineration and over 70 % landfilling or mismanagement.

Factors 1 and 2 result in lower emissions estimates compared to BAU projections. Factor 3 increases climate change impacts as all carbon within the plastic materials is released to air as CO_2 emissions. However, risk of toxic leaching from landfills (e.g., additives in plastics leached) is prevented.

S2.3 Comparison with previous studies: net-zero transition of the plastics industry

Our model assumptions and results with existing literature that deals with the transition of plastics / chemical industry are compared.

Table S25: Comparison with previous studies

	This study	Zheng et al.[90]	Meys et al.[22]	Stegmann et al.[12]	Meng et al.[91]	Bachmann et al.[25]	Gabrielli et al.[92]
Scope	Plastics	Plastics	Plastics	Plastics	Base chemicals	Plastics	Plastics & chemicals
**Strategies covered**							
Reduce demand	Yes	Yes	No	No	Yes	No	No
Continued fossil-based production	Yes	No	No	Yes	Yes	No	No
CO_2-based production	Yes	No	Yes	No	Yes	Yes	Yes (no combined routes)
Bio-based drop-in production	Yes	Yes	Yes	Yes	Yes	Yes	Yes
Non-drop-in plastics	Yes	Yes	No	No	No	No	No
Mechanical recycling	Yes	Yes	Yes	Yes	Yes	Yes	Yes
Chemical recycling	gasification	No	pyrolysis	pyrolysis	No	pyrolysis	No
Landfill as carbon sink	No	No	No	Yes	Yes	No	No
CCS	Yes	No	No	No	Yes	No	Yes
**Critical assumptions**							
Plastics demand in 2050 (Mt)	1007 (200-1400 as sensitivity)	1606 / 814 (two scenarios)	1418	1091 - 1137	1100	-	-

Continued on next page

Table S25 continued

	This study	Zheng et al.[90]	Meys et al.[22]	Stegmann et al.[12]	Meng et al.[91]	Bachmann et al.[25]	Gabrielli et al.[92]
Max. mechanical recycling rate	31% (0-45%, varying by plastic type)	44% (100% as sensitivity)	94% (mechanical and chemical recycling)[xix]	69%[xx]	41%	94%[xix] (74-94% as sensitivity, mechanical and chemical recycling)	75% (mechanical and chemical recycling)
Max. chemical recycling rate	31% (0-64%, varying by plastic type)	-		12%[xx]	-		
Electricity carbon footprint (g CO_2-eq/kWh)	7 (0-400 as sensitivity)	12 (544 as sensitivity)	7 (0-200 as sensitivity)	-13[xx]	0	7	0 (0-600 as sensitivity)
Biomass availability constraint (EJ)	37	-	-	NA (from TIMER model)	10[xxi]	-	-
**Analysis**							
Region-tailored strategies	Yes	No	No	Yes	No	No	No
Impacts beyond climate change	Burden on triple planetary crisis	No	No	No	No	Planetary boundary indicators	Land / water scarcity
Regional impacts	Yes	No	No	No	No	No	Yes
Biomass consumption (EJ)	37[xxii]	-	19.3/ 42.6[xxiii]	5.8[xx]	6[xxiv]	3.9	101[xxv]
Electricity consumption (PWh)	1.6[xxii]	-	9.8/2.7[xxiii]	3.5[xx]	10[xxiv]	0.78	32[xxv]

[xix] considering losses during sorting and recycling processes, the authors considered an effective recycling of 70%. The authors consider mechanical recycling for waste plastic packaging and chemical recycling for all plastic waste.

[xx] in scenario “circular bioeconomy”

[xxi] 20% of global availability

[xxii] net-zero scenario

[xxiii] (biomass, electricity) = (19.3 EJ, 9.8 PWh) and (42.6 EJ, 2.7 PWh) both could work

[xxiv] HC-NFAX scenario.

[xxv] single route only, biomass consumption: bio-based production route; electricity consumption: CO_2-based production route.

S2.4 Sensitivity analysis: gasification vs pyrolysis for chemical recycling

The net-zero scenario as presented in the main text chooses gasification as the chemical recycling method, and pyrolysis is not chosen by the optimization model at all. To understand the impacts of pyrolysis, we did a sensitivity analysis by disabling the gasification routes in the model. In such a case, the model would choose pyrolysis as the method for chemical recycling rather than direct incineration. Such a system with pyrolysis but not gasification would lead to a net climate change impact of $-0.20\,\mathrm{Gt\,CO_2}$-eq, which is slightly higher than the net-zero scenario of $-0.26\,\mathrm{Gt\,CO_2}$-eq, but still in the comparable range. This result is in line with Salah et al.[93], who found ethylene from plastic waste gasification route has lower, yet still comparable impacts in comparison to ethylene from plastic waste pyrolysis.

In addition, pyrolysis offers advantages as a retrofittable solution for existing petrochemical infrastructure, making it a potentially attractive short- to mid-term option for the current plastic industry. However, pyrolysis faces various technical and market challenges.[94,95] For example, high contaminant levels in pyrolysis oil products limit their application, with steam crackers typically accepting non-upgraded pyrolysis oil blends with naphtha at maximum levels of only 5% due to inconsistent feedstock quality.[96]

S2.5 Sensitivity analysis: without constaints on biodiversity loss

To avoid even higher biodiversity loss impacts, we have constrained the model from selecting biomass feedstock with biodiversity impacts exceeding 10^{-14} PDF kg^{-1} DM. Without this constraint, the biodiversity loss impacts would be doubled (Figure S9). But this change only bring marginal improvement in further reducing climate change impacts. This finding highlights the importance of considering regional variations in biomass sourcing to minimize environmental trade-offs of the net-zero transition of the plastic industry.

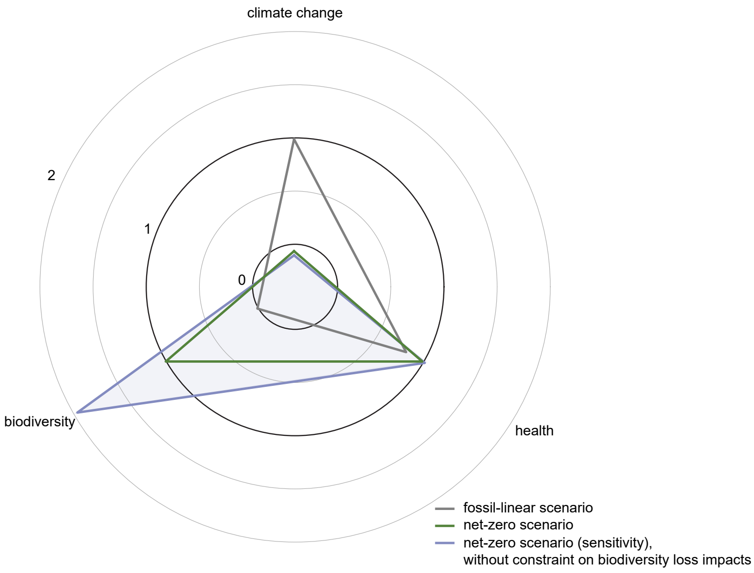

Figure S9: Sensitivity analysis of relative impacts, for the net-zero scenario without constraint on biodiversity loss impacts. Relative impacts on climate change, land use–related biodiversity loss, and PM-related health impacts of the fossil linear and the net-zero plastics industry (as in Figure 1 in the Main Text), and the impacts change of the net-zero scenario without the constraints on biodiversity loss impacts.

S2.6 Sensitivity analysis: impact of strategy implementation sequence

In Figure 1 of the Main Text, we illustrate the climate change impacts of transitioning from a fossil linear plastics industry to a net-zero scenario. We present the strategies in the following order: using alternative feedstock, implementing non-drop-in plastics, applying mechanical recycling, introducing chemical recycling (gasification), and implementing CCS. Each step adds one more strategy, and we rerun the model to determine the minimum climate change impacts of the plastics industry at each stage.

The sequence of applied strategies can influence the relative contribution of each added strategy, although the total achievement of strategy combinations is independent of the sequence. This is because the model selects different production routes as each new strategy is introduced. For instance, when mechanical recycling is applied directly to the fossil linear scenario, it decreases the impact by 1.36 Gt CO_2-eq (30 % of the fossil linear scenario). In contrast, as shown in Figure 1 of the Main Text, when mechanical recycling is implemented after alternative feedstock and non-drop-in plastics, it further reduces the impact by 0.98 Gt CO_2-eq (22 % of the fossil linear scenario).

Despite these variations, the relative importance of strategies remains largely consistent. Mechanical recycling, alternative feedstock and CCS maintain their predominant role in reducing GHG emissions from the fossil linear scenario.

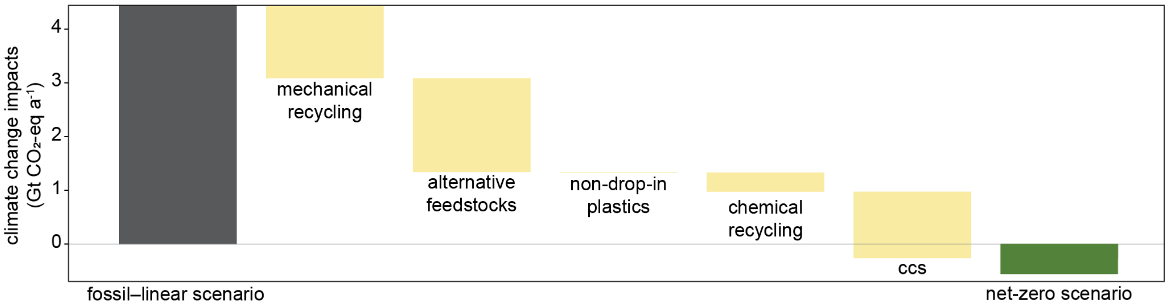

Figure S10: The climate change impacts of a fossil linear plastics industry and its transition into a net-zero one, with a different sequence of applying strategies.

S2.7 Sensitivity analysis: climate change impact of biogenic CO_2

Even if biomass is replanted, biogenic carbon emissions may contribute to climate change if there is a time gap between emission and resequestration by biomass.[84] Given the absence of standardized methods, we consider the climate change impacts of released biogenic CO_2 emissions using a worst-case scenario in the Main Text, assuming clear-cut of forests. The climate change impact of biogenic CO_2 is a function of the rotation period of the biomass.[84] Here, we conduct a sensitivity analysis assuming biogenic CO_2 released from forest residues has no impact on climate change (Figure S11). Under this sensitivity analysis, the climate change impacts of the net-zero scenario is -0.57 Gt CO_2-eq, compared to the baseline case of -0.26 Gt CO_2-eq. This difference stems not only from the varied contributions of

biogenic CO_2 (0.2 Gt CO_2-eq in the baseline case versus 0 in the sensitivity case) but also from the resulting difference in biomass utilization choices. In the sensitivity case, more biomass is used for heat production than for feedstock, reducing the need for natural gas and consequently lowering fossil CO_2 emissions.

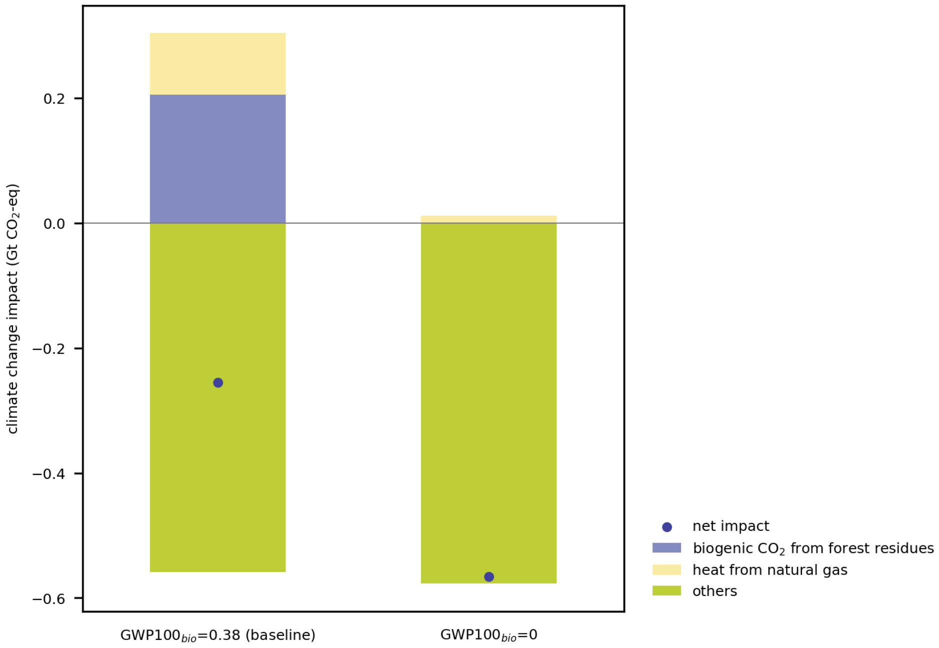

Figure S11: Climate change impacts of the net-zero plastics industry with two different accountings of the $GWP100_{bio}$.

S2.8 Sensitivity analysis: allocation method

The choice of allocation methods has limited impact on the climate change effects of the net-zero plastics industry, with the most significant differences arising when heat is co-produced. With system expansion by substitution, co-produced heat is assumed to be utilized within the system boundary, effectively reducing the need for primary heat production from biomass or natural gas by an equivalent amount. In contrast, economic allocation distributes the inventory among co-products based on their economic values. Since heat is relatively low-value compared to the main products, the majority of inventory flows—and consequently, impacts—are attributed to the primary product rather than the heat co-product. Consequently, system expansion results in lower climate change impacts from heat production compared to economic allocation, as less heat needs to be generated from natural gas. Impacts from other sources remain largely consistent regardless of the allocation method used.

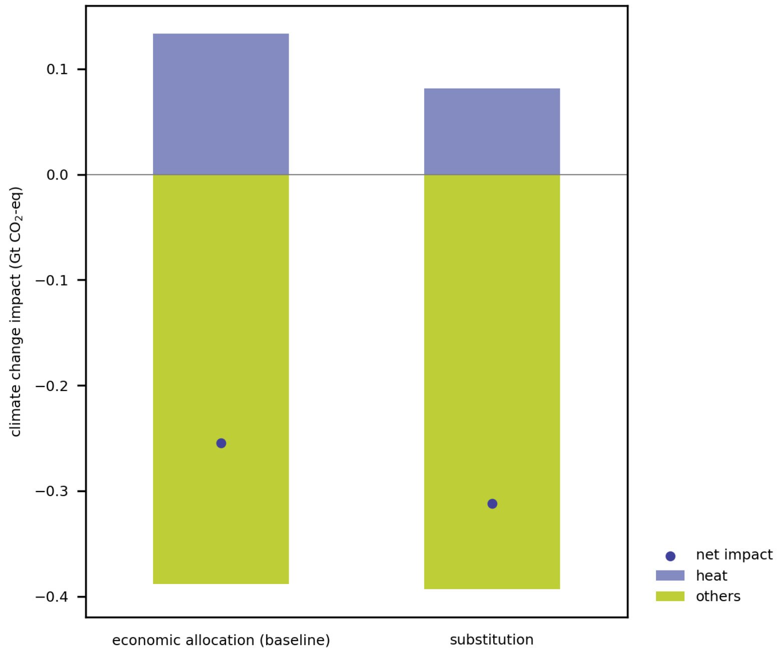

Figure S12: Climate change impacts of the net-zero plastics industry with two allocation methods.

S3 Study limitations

This study has several limitations:

- A key limitation of the study is the lack of an economic analysis. We optimize the plastics production routes by minimizing total climate change impacts because we want to assess whether and how net-zero is possible for plastic production and disposal. Our approach could lead the model to select very expensive mitigation routes, potentially raising the price of plastics and triggering subsequent effects on demand and market dynamics. Future studies should address this by incorporating an economic analysis to balance environmental goals with economic feasibility.

- While we consider the impacts from the production of plastics, the impact of plastic additives often goes unexamined. These additives are excluded from the gate-to-gate inventory due to their diversity, limited understanding, and small quantities used[97], typically below the cut-off we applied. However, their contribution to climate change remains uncertain. Moreover, plastic additives and chemicals pose threats to human health and biodiversity. For example, of over 16,000 identified chemicals in plastics, over 4,200 are “of concern” due to their persistence, bioaccumulation, mobility, or toxicity[98]. Widespread human exposure to these chemicals has been associated with increased risks of reproductive, neurodevelopmental, metabolic, and cardiovascular disorders, and cancers, among other adverse effects. The health costs by biaphenol A (BPA), di(2-ethylhexyl)phthalate (DEPH) and polybrominated biphenyl ethers (PBDEs) alone are estimated to be over 1.5 trillion US dollars annually globally[99]. Environmentally, plastics and plastic chemicals contaminate worldwide, harming marine and terrestrial species, degrading ecosystems, and promoting antimicrobial resistance[99].

- Furthermore, the release of micro- and nano-plastics through various pathways—such as manufacturing and processing, tire wear, textile washing, and landfill leaching—presents considerable risks to both human health and wildlife ecosystems.[66,67] While these impacts would be similar for both fossil-based and alternative feedstock-based drop-in plastics for the same type, they might differ for non-drop-in plastics. Piao et al.[100] revealed that highly biodegradable microplastics exhibit lower aquatic ecotoxicity but higher greenhouse gas emissions. However, we excluded these impacts from our analysis due to their diverse applications and the substantial uncertainty this would introduce at a global scale. Additionally, our assumption that all non-recyclable plastic waste will be incinerated by 2050 is ambitious. Recent methodological advances of quantifying the impact of microplastics in LCA show promise—including the development of characterization factors for assessing aquatic microplastic emissions across eleven polymers, three shapes, and five sizes.[101] However, it is still challenging to include microplastic impacts in our global-scale analysis. Data gaps exist regarding leakage pathways and microplastic generation quantities during the use phase, while fate factors for mixed plastic waste streams remain particularly difficult to define due to their dependence on variable particle size, density, polymer type, and degradation and fragmentation behavior across heterogeneous waste compositions. Given the high

variability in both leakage rates and degradation rates across different environmental conditions for mixed plastic waste, future research is warranted to develop robust methodological frameworks that can adequately capture these complex microplastic dynamics in comprehensive LCA studies.

- Our biomass consideration is limited to the availability and impacts of lignocellulose residues. While the potential use of biogas, particularly as a heat source, could reduce PM-related health impacts, it is not included in the model due to the lack of information on regional availability and impacts for 2050.

- While we consider the substitution of non-drop-in plastics with conventional plastic types, the substitution between different conventional plastics (e.g., replacing polyurethane with polyethylene in certain applications) is not considered. As some plastic types have lower impacts than others, such substitution could further reduce the overall impacts. In addition, there might be other entirely new production routes and plastic types that could substitute the existing ones that offer the possibility to further lower the climate change impacts.

- Our model uses pre-allocated unit processes for most multi-output operations, except for methanol-to-olefins (MTO) process (see Methods in the Main Text, for details). This approach allows the model to select individual products (e.g., only ethylene from steam cracking) without considering physical relationships between co-products (e.g. co-production of propylene). While constraints are implemented for the crucial MTO process, they are not applied to others due to data limitations and configuration versatility.

- While there is limited room for efficiency improvements in fossil-based or drop-in processes, many other processes in our model, such as non-drop-in bio-based plastics production, are still under development and their efficiency may improve over time. Hence, these processes may play a more significant role in the future than anticipated by our current model. In addition, if biodegradable plastics are properly sorted, collected, and sent to industrial anaerobic digestion facilities where biogas can be recovered and utilized, this could represent a viable end-of-life plastic waste treatment option that can be included in the future version of our optimization model.

- Our global optimization model assumes an ideal global supply chain, where resources can be freely transported across borders to meet high demand. However, this overlooks the logistical challenges of transporting widespread, low-density biomass residues. Long-distance transportation may prove costly and impractical, and it could potentially increase GHG and PM emissions if implemented.[19] A simplified sensitivity analysis shows that transporting all available 2.25 Gt lignocellulose residues for 100 km results in approximately 22 Mt CO_2-eq emissions (compared to -260 Mt CO_2-eq for the net-zero scenario). One solution could be to establish new biorefineries near biomass sources, processing raw materials into marketable intermediate products locally. While this approach may address logistical issues, it could also constrain the scale of biorefinery operations.

- Our study focuses solely on capturing CO_2 emissions from high-purity sources, such as fermentation and gasification processes. These sources are easier to capture and have already been commercialized, particularly in ethanol production where CO_2 is often upgraded to food-grade quality.[102] However, we recognize the potential to capture CO_2 from other emission sources, including combustion processes for heat production. Although these sources typically have lower CO_2 concentrations (usually below 30 %) and thus offer less GHG savings at higher costs[19,103], they represent additional opportunities for the plastic industry to achieve net-zero emissions.

S4 References

1. Stehfest, E. *et al. Integrated assessment of global environmental change with IMAGE 3.0. Model description and policy applications* Report (PBL Netherlands Environmental Assessment Agency, 2014).
2. PBL Netherlands Environmental Assessment Agency. *Region classification map* Web Page. `https://models.pbl.nl/image/Region_classification_map`. Accessed on 2024-07-26. 2018.
3. Khanam, P. N. & AlMaadeed, M. A. A. Processing and characterization of polyethylene-based composites. *Advanced Manufacturing-Polymer & Composites Science* **1,** 63–79. doi:`10.1179/2055035915y.0000000002` (2015).
4. Plastics Europe. *Polyolefins* Web Page. `https://plasticseurope.org/plastics-explained/a-large-family/polyolefins/`. Accessed on 2024-06-27. 2024.
5. British Plastics Federation. *Plastipedia–The Web's Largest Plastics Encyclopedia* Web Page. `https://www.bpf.co.uk/Plastipedia/`. Accessed on 2024-06-27. 2024.
6. Leejarkpai, T., Mungcharoen, T. & Suwanmanee, U. Comparative assessment of global warming impact and eco-efficiency of PS (polystyrene), PET (polyethylene terephthalate) and PLA (polylactic acid) boxes. *Journal of Cleaner Production* **125,** 95–107. doi:`10.1016/j.jclepro.2016.03.029` (2016).
7. Matmatch. *Polyurethane: Properties, Processing, and Applications* Web Page. `https://matmatch.com/learn/material/polyurethane`. Accessed on 2024-06-27.
8. Su, S. Compatibilization, processing and characterization of poly(butylene adipate terephthalate) / polylactide (PBAT/PLA) blends. *Materials Research Express* **9.** doi:`10.1088/2053-1591/ac55c7` (2022).
9. Aliotta, L., Seggiani, M., Lazzeri, A., Gigante, V. & Cinelli, P. A Brief Review of Poly (Butylene Succinate) (PBS) and Its Main Copolymers: Synthesis, Blends, Composites, Biodegradability, and Applications. *Polymers* **14.** doi:`10.3390/polym14040844` (2022).
10. De Jong, E., Dam, M. A., Sipos, L. & Gruter, G. J. M. Furandicarboxylic Acid (FDCA), A Versatile Building Block for a Very Interesting Class of Polyesters. *Biobased Monomers, Polymers, and Materials* **1105,** 1–13 (2012).
11. Al, G., Aydemir, D., Kaygin, B., Ayrilmis, N. & Gunduz, G. Preparation and characterization of biopolymer nanocomposites from cellulose nanofibrils and nanoclays. *Journal of Composite Materials* **52,** 689–700. doi:`10.1177/0021998317713589` (2018).
12. Stegmann, P., Daioglou, V., Londo, M., van Vuuren, D. P. & Junginger, M. Plastic futures and their CO_2 emissions. *Nature* **612,** 272–+. doi:`10.1038/s41586-022-05422-5` (2022).
13. Geyer, R., Jambeck, J. R. & Law, K. L. Production, use, and fate of all plastics ever made. *Science Advances* **3.** doi:`10.1126/sciadv.1700782` (2017).

14. Klotz, M., Haupt, M. & Hellweg, S. Potentials and limits of mechanical plastic recycling. *Journal of Industrial Ecology* **27,** 1043–1059. doi:10.1111/jiec.13393 (2023).

15. Klotz, M., Haupt, M. & Hellweg, S. Limited utilization options for secondary plastics may restrict their circularity. *Waste Management* **141,** 251–270. doi:https://doi.org/10.1016/j.wasman.2022.01.002 (2022).

16. IHS Markit. *Petrochemical capacity in China and Middle East: Growth amid challenges* Web Page. https://ihsmarkit.com/research-analysis/petrochemical-capacity-growth-in-china-middle-east.html. Accessed on 2021-10-01. 2021.

17. LexisNexis Risk Solutions. *ICIS Chemical Business* Report (2020).

18. Allport, D. C., Gilbert, D. S. & Outterside, S. M. in *MDI and TDI: Safety, Health and the Environment* 11–23 (2003). doi:https://doi.org/10.1002/0470865687.ch1.

19. Huo, J., Wang, Z. Y., Oberschelp, C., Guillen-Gosalbez, G. & Hellweg, S. Net-zero transition of the global chemical industry with CO_2-feedstock by 2050: feasible yet challenging. *Green Chemistry* **25,** 415–430. doi:10.1039/d2gc03047k (2023).

20. Huo, J. *et al.* Region-specific sourcing of lignocellulose residues as renewable feedstocks for a net-zero chemical industry. *Environmental Science & Technology* **58,** 13748–13759. doi:10.1021/acs.est.4c03005 (2024).

21. Bazzanella, A. M. & Ausfelder, F. *Low carbon energy and feedstock for the European chemical industry* Report (DECHEMA, 2017).

22. Meys, R. *et al.* Achieving net-zero greenhouse gas emission plastics by a circular carbon economy. *Science* **374,** 71–76. doi:10.1126/science.abg9853 (2021).

23. IHS Markit. *Process Economics Program (PEP) Yearbook* Report (2021).

24. Wu, Y. Q., Xiang, Y. L., Cai, L., Liu, H. T. & Liang, Y. Optimization of a novel cryogenic air separation process based on cold energy recovery of LNG with exergoeconomic analysis. *Journal of Cleaner Production* **275.** doi:10.1016/j.jclepro.2020.123027 (2020).

25. Bachmann, M. *et al.* Towards circular plastics within planetary boundaries. *Nature Sustainability* **6,** 599–610. doi:10.1038/s41893-022-01054-9 (2023).

26. Liu, F., Guo, X., Wang, Y., Chen, G. Y. & Hou, L. A. Process simulation and economic and environmental evaluation of a corncob-based biorefinery system. *Journal of Cleaner Production* **329.** doi:10.1016/j.jclepro.2021.129707 (2021).

27. Hoppe, W., Thonemann, N. & Bringezu, S. Life Cycle Assessment of Carbon Dioxide-Based Production of Methane and Methanol and Derived Polymers. *Journal of Industrial Ecology* **22,** 327–340. doi:10.1111/jiec.12583 (2018).

28. Nitzsche, R., Budzinski, M. & Gröngröft, A. Techno-economic assessment of a wood-based biorefinery concept for the production of polymer-grade ethylene, organosolv lignin and fuel. *Bioresource Technology* **200,** 928–939. doi:10.1016/j.biortech.2015.11.008 (2016).

29. Gerloff, N. Comparative Life-Cycle-Assessment analysis of three major water electrolysis technologies while applying various energy scenarios for a greener hydrogen production. *Journal of Energy Storage* **43.** doi:10.1016/j.est.2021.102759 (2021).

30. Bai, Z., Liu, Q. B., Gong, L. & Lei, J. Investigation of a solar-biomass gasification system with the production of methanol and electricity: Thermodynamic, economic and off-design operation. *Applied Energy* **243,** 91–101. doi:10.1016/j.apenergy.2019.03.132 (2019).

31. Harris, K., Grim, R. G., Huang, Z. & Tao, L. A comparative techno-economic analysis of renewable methanol synthesis from biomass and CO_2: Opportunities and barriers to commercialization. *Applied Energy* **303.** doi:10.1016/j.apenergy.2021.117637 (2021).

32. Poluzzi, A., Guandalini, G. & Romano, M. C. Flexible methanol and hydrogen production from biomass gasification with negative emissions. *Sustainable Energy & Fuels* **6,** 3830–3851. doi:10.1039/d2se00661h (2022).

33. Charalambous, M. A., Tulus, V., Ryberg, M. W., Pérez-Ramírez, J. & Guillén-Gosálbez, G. Absolute environmental sustainability assessment of renewable dimethyl ether fuelled heavy-duty trucks. *Sustainable Energy & Fuels* **7,** 1930–1941. doi:10.1039/d2se01409b (2023).

34. González-Garay, A. *et al.* Plant-to-planet analysis of CO_2-based methanol processes. *Energy & Environmental Science* **12,** 3425–3436. doi:10.1039/c9ee01673b (2019).

35. Khojasteh-Salkuyeh, Y., Ashrafi, O., Mostafavi, E. & Navarri, P. CO_2 utilization for methanol production; Part I: Process design and life cycle GHG assessment of different pathways. *Journal of CO_2 Utilization* **50,** 101608. doi:10.1016/j.jcou.2021.101608 (2021).

36. Nizami, M., Slamet & Purwanto, W. W. Solar PV based power-to-methanol via direct CO_2 hydrogenation and H2O electrolysis: Techno-economic and environmental assessment. *Journal of CO_2 Utilization* **65.** doi:10.1016/j.jcou.2022.102253 (2022).

37. Rebolledo-Leiva, R. *et al.* Pursuing single or combined wheat straw based poly(butylene succinate) production routes: A life cycle approach of first- and second-generation feedstocks. *Sustainable Materials and Technologies* **37.** doi:10.1016/j.susmat.2023.e00683 (2023).

38. Kim, H., Baek, S. & Won, W. Integrative technical, economic, and environmental sustainability analysis for the development process of biomass-derived 2,5 -furandicarboxylic acid. *Renewable & Sustainable Energy Reviews* **157.** doi:10.1016/j.rser.2021.112059 (2022).

39. Mandegari, M. A., Farzad, S., van Rensburg, E. & Görgens, J. F. Multi-criteria analysis of a biorefinery for co-production of lactic acid and ethanol from sugarcane lignocellulose. *Biofuels Bioproducts & Biorefining-Biofpr* **11,** 971–990. doi:10.1002/bbb.1801 (2017).

40. Akiyama, M., Tsuge, T. & Doi, Y. Environmental life cycle comparison of polyhydroxyalkanoates produced from renewable carbon resources by bacterial fermentation. *Polymer Degradation and Stability* **80,** 183–194. doi:10.1016/S0141-3910(02)00400-7 (2003).

41. Cappiello, G., Aversa, C., Genovesi, A. & Barletta, M. Life cycle assessment (LCA) of bio-based packaging solutions for extended shelf-life (ESL) milk. *Environmental Science and Pollution Research* **29,** 18617–18628. doi:10.1007/s11356-021-17094-1 (2022).

42. Nessi, S. *et al. Life Cycle Assessment of alternative feedstocks for plastics production – Part 2: illustrative case studies* Report (Joint Research Centre (JRC), 2022). doi:10.2760/655230.

43. Gironi, F. & Piemonte, V. Life Cycle Assessment of Polylactic Acid and Polyethylene Terephthalate Bottles for Drinking Water. *Environmental Progress & Sustainable Energy* **30,** 459–468. doi:10.1002/ep.10490 (2011).

44. Papong, S. *et al.* Comparative assessment of the environmental profile of PLA and PET drinking water bottles from a life cycle perspective. *Journal of Cleaner Production* **65,** 539–550. doi:10.1016/j.jclepro.2013.09.030 (2014).

45. Baldowska-Witos, P. *et al.* Life Cycle Assessment of Two Alternative Plastics for Bottle Production. *Materials* **14.** doi:10.3390/ma14164552 (2021).

46. Tamburini, E. *et al.* Plastic (PET) vs bioplastic (PLA) or refillable aluminium bottles—What is the most sustainable choice for drinking water? A life-cycle (LCA) analysis. *Environmental Research* **196.** doi:10.1016/j.envres.2021.110974 (2021).

47. Desole, M. P., Aversa, C., Barletta, M., Gisario, A. & Vosooghnia, A. Life cycle assessment (LCA) of PET and PLA bottles for the packaging of fresh pasteurised milk: The role of the manufacturing process and the disposal scenario. *Packaging Technology and Science* **35,** 135–152. doi:10.1002/pts.2615 (2022).

48. Rattana, S. & Gheewala, S. H. Environment impacts assessment of petroleum plastic and bioplastic carrier bags in Thailand. *Journal of Sustainable Energy & Environment* **10,** 9–17 (2019).

49. Madival, S., Auras, R., Singh, S. P. & Narayan, R. Assessment of the environmental profile of PLA, PET and PS clamshell containers using LCA methodology. *Journal of Cleaner Production* **17,** 1183–1194. doi:10.1016/j.jclepro.2009.03.015 (2009).

50. UNEP. *Single-use plastic take-away food packaging and its alternatives - Recommendations from Life Cycle Assessments* Report (United Nations Environment Programme, 2020).

51. Lorite, G. S. *et al.* Evaluation of physicochemical/microbial properties and life cycle assessment (LCA) of PLA-based nanocomposite active packaging. *Lwt-Food Science and Technology* **75,** 305–315. doi:10.1016/j.lwt.2016.09.004 (2017).

52. Uihlein, A., Ehrenberger, S. & Schebek, L. Utilisation options of renewable resources: a life cycle assessment of selected products. *Journal of Cleaner Production* **16,** 1306–1320. doi:10.1016/j.jclepro.2007.06.009 (2008).

53. Potting, J. & van der Harst, E. Facility arrangements and the environmental performance of disposable and reusable cups. *International Journal of Life Cycle Assessment* **20,** 1143–1154. doi:10.1007/s11367-015-0914-7 (2015).

54. Fieschi, M. & Pretato, U. Role of compostable tableware in food service and waste management. A life cycle assessment study. *Waste Management* **73,** 14–25. doi:10.1016/j.wasman.2017.11.036 (2018).

55. For Research, D.-G. & Innovation. *Environmental impact assessments of innovative bio-based product* Report (European Commission, 2019). doi:https://data.europa.eu/doi/10.2777/251887.

56. Americas, P. *Comparative Life Cycle Assessment Ingeo™ biopolymer, PET, and PP Drinking Cups* Report (2009).

57. Moretti, C. *et al.* Cradle-to-grave life cycle assessment of single-use cups made from PLA, PP and PET. *Resources Conservation and Recycling* **169.** doi:10.1016/j.resconrec.2021.105508 (2021).

58. Bohlmann, G. M. Biodegradable packaging life-cycle assessment. *Environmental Progress* **23,** 342–346. doi:10.1002/ep.10053 (2004).

59. Deng, Y. L., Achten, W. M. J., Van Acker, K. & Duflou, J. R. Life cycle assessment of wheat gluten powder and derived packaging film. *Biofuels Bioproducts & Biorefining-Biofpr* **7,** 429–458. doi:10.1002/bbb.1406 (2013).

60. Hakala, S., Virtanen, Y., Meinander, K. & Tanner, T. *Life-cycle assessment, comparison of biopolymer and traditional diaper systems* Report (Technical Research Centre of Finland, VTT Tiedotteita, 1997).

61. Schrijvers, D. L., Leroux, F., Verney, V. & Patel, M. K. life cycle assessment of polymer nanocomposites using organo-modified layered double hydroxides for potential application in agricultural films. *Green Chemistry* **16,** 4969–4984. doi:10.1039/c4gc00830h (2014).

62. Tan, Q. Y., Yang, L. Y., Wei, F., Chen, Y. & Li, J. H. Comparative life cycle assessment of polyethylene agricultural mulching film and alternative options including different end-of-life routes. *Renewable & Sustainable Energy Reviews* **178.** doi:10.1016/j.rser.2023.113239 (2023).

63. Patel, M. K. *et al.* Second-generation bio-based plastics are becoming a reality - Non-renewable energy and greenhouse gas (GHG) balance of succinic acid-based plastic end products made from lignocellulosic biomass. *Biofuels Bioproducts & Biorefining-Biofpr* **12,** 426–441. doi:10.1002/bbb.1849 (2018).

64. Thrän, J. *et al.* Environmental and economic assessment of biodegradable and compostable alternatives for plastic materials in greenhouses. *Waste Management* **175,** 92–100. doi:10.1016/j.wasman.2023.12.049 (2024).

65. the European Parliament and the Council of the European Union. *Directive (EU) 2018/850 of the European Parliament and of the Council of 30 May 2018 amending Directive 1999/31/EC on the landfill of waste (Text with EEA relevance)* Directive (2018).

66. Kwan, C. S. & Takada, H. in *Hazardous Chemicals Associated with Plastics in the Marine Environment* (eds Takada, H. & Karapanagioti, H. K.) 51–70 (Springer, Cham, 2017). doi:https://doi.org/10.1007/698_2016_122.

67. Bridson, J. H., Gaugler, E. C., Smith, D. A., Northcott, G. L. & Gaw, S. Leaching and extraction of additives from plastic pollution to inform environmental risk: A multidisciplinary review of analytical approaches. *Journal of Hazardous Materials* **414.** doi:10.1016/j.jhazmat.2021.125571 (2021).

68. Budin, S., Maideen, N. C., Koay, M. H., Ibrahim, D. & Yusoff, H. A comparison study on mechanical properties of virgin and recycled polylactic acid (PLA). *Journal of Physics: Conference Series* **1349,** 012002. doi:10.1088/1742-6596/1349/1/012002 (Nov. 2019).

69. Prifti, K., Galeazzi, A. & Manenti, F. Design and Simulation of a Plastic Waste to Methanol Process: Yields and Economics. *Industrial & Engineering Chemistry Research* **62,** 5083–5096. doi:10.1021/acs.iecr.2c03929 (2023).

70. Kumar, R., Sadeghi, K., Jang, J. & Seo, J. Mechanical, chemical, and bio-recycling of biodegradable plastics: A review. *Science of The Total Environment* **882,** 163446. doi:https://doi.org/10.1016/j.scitotenv.2023.163446 (2023).

71. FOEN. *Plastics in the environment: biodegradable plastics* Report (Swiss Federal Office for the Environment, 2020).

72. Sacchi, R. *et al.* PRospective EnvironMental Impact asSEment (premise): A streamlined approach to producing databases for prospective life cycle assessment using integrated assessment models. *Renewable & Sustainable Energy Reviews* **160.** doi:10.1016/j.rser.2022.112311 (2022).

73. Nielsen, O. *et al.* in *EMEP/EEA air pollutant emission inventory guidebook* chap. 1.A.1 (Publications Office of the European Union, Copenhagen, Denmark, 2023).

74. Olsson, O. & Schipfer, F. *Decarbonizing industrial process heat: the role of biomass* Report (IEA Bioenergy, 2021).

75. FitzGerald, D. *et al. Documentation of changes implemented in the ecoinvent database v3.10* Report (ecoinvent, 2023).

76. the European Parliament and the Council of the European Union. *DIRECTIVE 2010/75/EU OF THE EUROPEAN PARLIAMENT AND OF THE COUNCIL of 24 November 2010 on industrial emissions (integrated pollution prevention and control) (Recast) (Text with EEA relevance)* Directive (2010).

77. IPCC. *2006 IPCC Guidelines for National Greenhouse Gas Inventories - Volume 3 - Industrial Processes and Product Use* Report (Intergovernmental Panel on Climate Change, 2006).

78. The Engineering Toolbox. *Gases—Densities* Web Page. https://www.engineeringtoolbox.com/gas-density-d_158.html. Accessed on 2024-07-29. 2003.

79. The Engineering Toolbox. *Fuels—Higher and Lower Calorific Values* Web Page. https://www.engineeringtoolbox.com/fuels-higher-calorific-values-d_169.html. Accessed on 2024-07-29. 2003.

80. IPCC. *2006 IPCC Guidelines for National Greenhouse Gas Inventories - Volume 2 - Energy* Report (Intergovernmental Panel on Climate Change, 2006).

81. ecoinvent. *System Models* Web Page. https://support.ecoinvent.org/system-models#Allocation_classification. Accessed on 2024-07-29. 2024.

82. Vassilev, S. V., Baxter, D., Andersen, L. K. & Vassileva, C. G. An overview of the chemical composition of biomass. *Fuel* **89,** 913–933. doi:10.1016/j.fuel.2009.10.022 (2010).

83. Searle, S. & Malins, C. A reassessment of global bioenergy potential in 2050. *Global Change Biology Bioenergy* **7,** 328–336. doi:10.1111/gcbb.12141 (2015).

84. Cherubini, F., Peters, G. P., Berntsen, T., Stromman, A. H. & Hertwich, E. CO_2 emissions from biomass combustion for bioenergy: atmospheric decay and contribution to global warming. *Global Change Biology Bioenergy* **3,** 413–426. doi:10.1111/j.1757-1707.2011.01102.x (2011).

85. Pittau, F., Krause, F., Lumia, G. & Habert, G. Fast-growing bio-based materials as an opportunity for storing carbon in exterior walls. *Building and Environment* **129,** 117–129. doi:10.1016/j.buildenv.2017.12.006 (2018).

86. Scherer, L. *et al.* Biodiversity Impact Assessment Considering Land Use Intensities and Fragmentation. *Environmental Science & Technology* **57,** 19612–19623. doi:10.1021/acs.est.3c04191 (2023).

87. Gogate, M. R. Methanol-to-olefins process technology: current status and future prospects. *Petroleum Science and Technology* **37,** 559–565. doi:10.1080/10916466.2018.1555589 (2019).

88. Spierling, S. *et al.* Bio-based plastics - A review of environmental, social and economic impact assessments. *Journal of Cleaner Production* **185,** 476–491. doi:10.1016/j.jclepro.2018.03.014 (2018).

89. Cabernard, L., Pfister, S., Oberschelp, C. & Hellweg, S. Growing environmental footprint of plastics driven by coal combustion. *Nature Sustainability* **5,** 139–148. doi:10.1038/s41893-021-00807-2 (2022).

90. Zheng, J. J. & Suh, S. Strategies to reduce the global carbon footprint of plastics. *Nature Climate Change* **9,** 374–+. doi:10.1038/s41558-019-0459-z (2019).

91. Meng, F. R. *et al.* Planet-compatible pathways for transitioning the chemical industry. *Proceedings of the National Academy of Sciences of the United States of America* **120.** doi:10.1073/pnas.2218294120 (2023).

92. Gabrielli, P. *et al.* Net-zero emissions chemical industry in a world of limited resources. *One Earth* **6,** 682–704. doi:10.1016/j.oneear.2023.05.006 (2023).

93. Salah, C. *et al.* Environmental Benefits of Circular Ethylene Production from Polymer Waste. *ACS Sustainable Chemistry & Engineering* **12,** 13897–13906. doi:10.1021/acssuschemeng.4c04241. eprint: https://doi.org/10.1021/acssuschemeng.4c04241 (2024).

94. Qureshi, M. S. *et al.* Pyrolysis of plastic waste: Opportunities and challenges. *Journal of Analytical and Applied Pyrolysis* **152.** doi:10.1016/j.jaap.2020.104804 (2020).

95. Kusenberg, M. *et al.* Opportunities and challenges for the application of post-consumer plastic waste pyrolysis oils as steam cracker feedstocks: To decontaminate or not to decontaminate? *Waste Management* **138,** 83–115. doi:10.1016/j.wasman.2021.11.009 (2022).

96. LexisNexis Risk Solutions. *Mixed Plastic Waste and Pyrolysis Oil Methodology* Report (2023).

97. Wiesinger, H., Wang, Z. Y. & Hellweg, S. Deep Dive into Plastic Monomers, Additives, and Processing Aids. *Environmental Science & Technology* **55,** 9339–9351. doi:10.1021/acs.est.1c00976 (2021).

98. Monclús, L. *et al.* Mapping the chemical complexity of plastics. *Nature* **643,** 349–355. doi:10.1038/s41586-025-09184-8 (2025).

99. Landrigan, P. J. *et al.* The Lancet Countdown on health and plastics. *The Lancet.* doi:https://doi.org/10.1016/S0140-6736(25)01447-3 (2025).

100. Piao, Z., Agyei Boakye, A. A. & Yao, Y. Environmental impacts of biodegradable microplastics. *Nature Chemical Engineering* **1,** 661–669. doi:10.1038/s44286-024-00127-0 (2024).

101. Corella-Puertas, E., Hajjar, C., Lavoie, J. & Boulay, A.-M. MarILCA characterization factors for microplastic impacts in life cycle assessment: Physical effects on biota from emissions to aquatic environments. *Journal of Cleaner Production* **418,** 138197. doi:https://doi.org/10.1016/j.jclepro.2023.138197 (2023).

102. Ramdon. *Production Process of Beverage Grade CO_2: Ensuring Purity and Compliance* Web Page. https://ramdon.com/production-process-of-beverage-grade-co2/. Accessed on 2024-08-15. 2024.

103. Von der Assen, N., Müller, L. J., Steingrube, A., Voll, P. & Bardow, A. Selecting CO_2 Sources for CO_2 Utilization by Environmental-Merit-Order Curves. *Environmental Science & Technology* **50,** 1093–1101. doi:10.1021/acs.est.5b03474 (2016).
